# A giant tyrannosaur from the Campanian–Maastrichtian of southern North America and the evolution of tyrannosaurid gigantism

**DOI:** 10.1038/s41598-023-47011-0

**Published:** 2024-01-11

**Authors:** Sebastian G. Dalman, Mark A. Loewen, R. Alexander Pyron, Steven E. Jasinski, D. Edward Malinzak, Spencer G. Lucas, Anthony R. Fiorillo, Philip J. Currie, Nicholas R. Longrich

**Affiliations:** 1 New Mexico Museum of Natural History and Science, 1801 Mountain Road N.W., Albuquerque, NM 87104 USA; 2 Department of Geology and Geophysics, University of Utah, Salt Lake City, UT USA; 3 Natural History Museum of Utah, University of Utah, Salt Lake City, UT USA; 4 Department of Biological Sciences, The George Washington University, 2023 G St. NW, Washington, DC 20052 USA; 5 Department of Environmental Science and Sustainability, Harrisburg University, 326 Market Street, Harrisburg, PA 17101 USA; 6 Pennsylvania State University, Lehigh Valley, Center Valley, PA 18034 USA; 7 Department of Biological Sciences, University of Alberta, Edmonton, AB T6G 2E9 Canada; 8 Department of Biology and Biochemistry, University of Bath, Bath, BA2 7AY UK

**Keywords:** Palaeontology, Biogeography, Ecology, Evolution

## Abstract

Tyrannosaurid dinosaurs dominated as predators in the Late Cretaceous of Laurasia, culminating in the evolution of the giant *Tyrannosaurus rex*, both the last and largest tyrannosaurid. Where and when Tyrannosaurini (*T. rex* and kin) originated remains unclear. Competing hypotheses place tyrannosaurin origins in Asia, or western North America (Laramidia). We report a new tyrannosaurin, *Tyrannosaurus mcraeensis*, from the Campanian–Maastrichtian Hall Lake Formation of New Mexico, based on a fossil previously referred to *T. rex*. *T. mcraeensis* predates *T. rex* by ~ 6–7 million years, yet rivaled it in size. Phylogenetic analysis recovers *T. mcraeensis* as sister to *T. rex* and suggests Tyrannosaurini originated in southern Laramidia. Evolution of giant tyrannosaurs in southern North America, alongside giant ceratopsians, hadrosaurs, and titanosaurs suggests large-bodied dinosaurs evolved at low latitudes in North America.

## Introduction

Tyrannosaurids were the dominant predators in North America and Asia during the Late Cretaceous^1–3^. Evolving from small-bodied ancestors in the mid-Cretaceous, tyrannosaurids became apex predators in the latest Cretaceous, and finally saw the appearance of *Tyrannosaurus rex*^4,5^. *T. rex*, characterized by a robust skeleton and powerful, bone-crushing jaws^6^, was the dominant carnivore in the late Maastrichtian of western North America^3,7^. Growing to 12 m long and ~ 10 tons in weight^8^, *T. rex* was the largest terrestrial predator of its time, and perhaps of all time.

**Figure 1:**
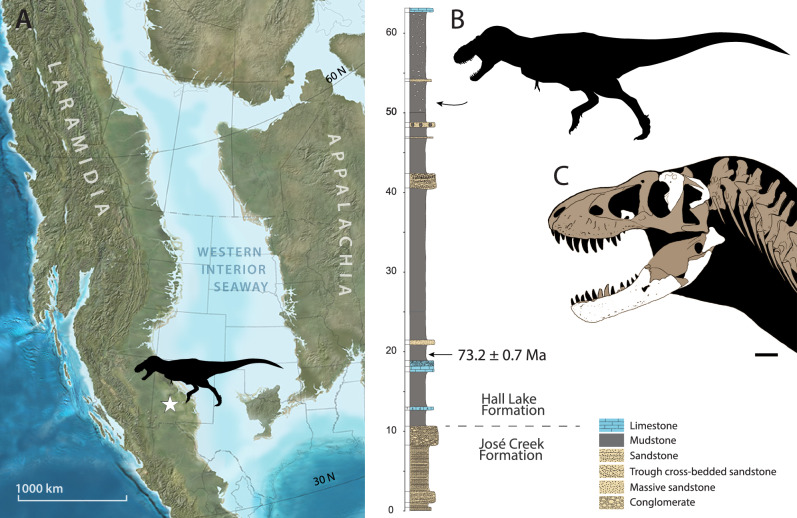
Locality and stratigraphy of *Tyrannosaurus mcraeensis* gen. et sp. nov., NMMNH P-3698. (**A**), type locality in Sierra County, New Mexico; (**B**), stratigraphy of fossil and the Hall Lake Formation (**C**), recovered skull elements. Scale = 10 cm. Map by Ron Blakey.

**Figure 2:**
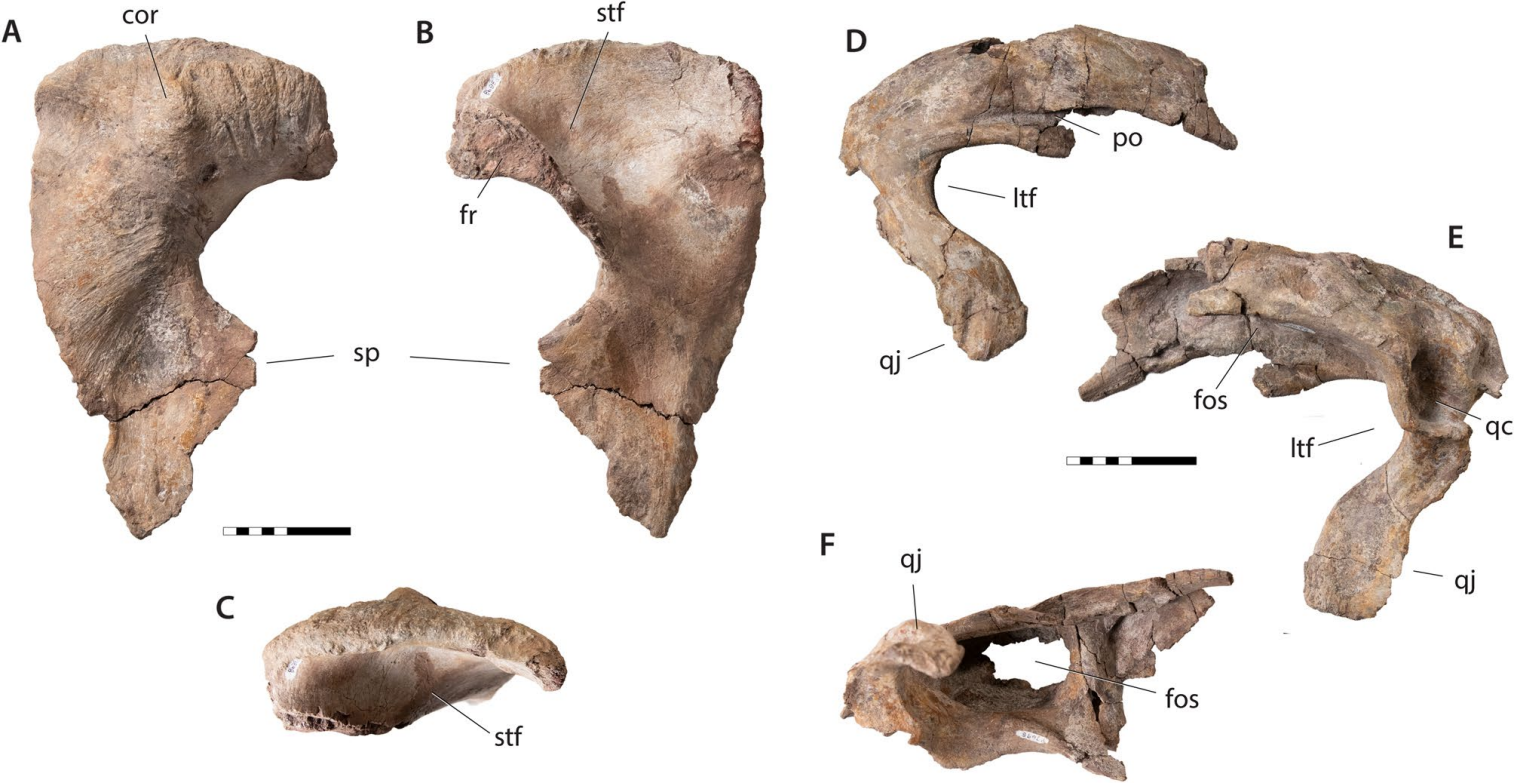
Cranial elements of *Tyrannosaurus mcraeensis* gen. et sp. nov. (NMMNH P-3698). Right postorbital in (**A**), lateral view; (**B**), medial view; (**C**), dorsal view. Right squamosal in (**D**), lateral view; (**E**), medial view; (**F**), ventral view. *cor* cornual boss, *fos* ventral fossa, *fr* frontal articulation, *ltf* lateral temporal fenestra, *po* postorbital articulation, *sp* suborbital process, *stf* supratemporal fossa, *qc* quadrate cotyle, *qj* quadratojugal process. Scale = 10 cm.

**Figure 3:**
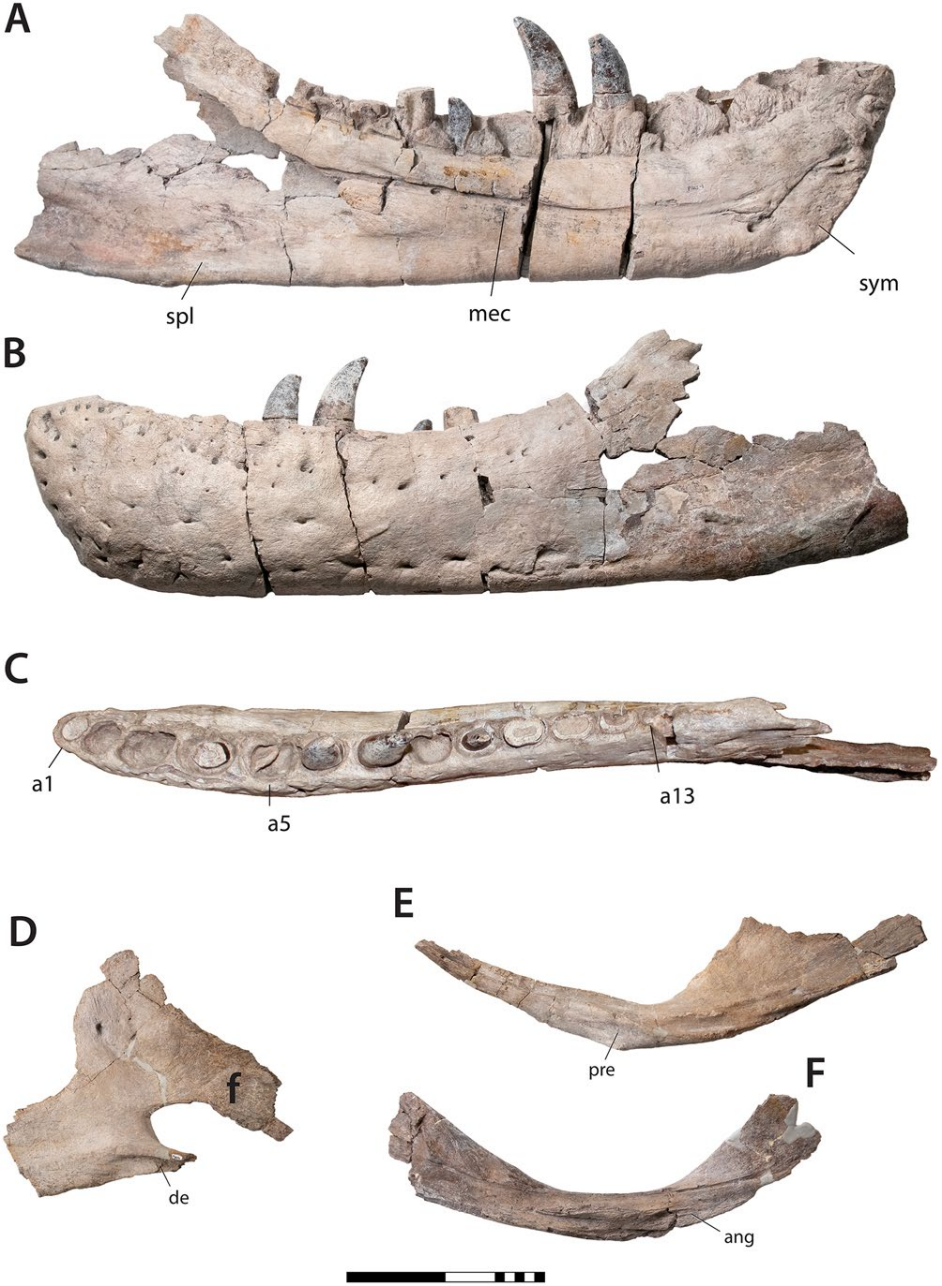
Mandibular elements of *Tyrannosaurus mcraeensis* gen. et sp. nov. (NMMNH P-3698). Left dentary in (**A**), medial view; (**B**), lateral view; (**C**), dorsal view; (**D**), right splenial, medial view; (**E**), right angular, medial view; (**F**), right prearticular, medial view. *ang* angular contact, *de* shelf for dentary, *mec* Meckelian canal, *pre* prearticular facet, *a1, a5, a15* alveoli 1, 5, and 15, *sp* splenial, *sym* symphysis. Scale = 20 cm.

The origins of the *Tyrannosaurus* lineage, Tyrannosaurini, are unknown. *Tyrannosaurus rex* appeared suddenly in the latest Maastrichtian^3,7^. No close relatives have been reported from North America prior to this time. Instead, the closest relatives of *T. rex* come from Mongolia. To explain this pattern, one hypothesis suggests tyrannosaurins dispersed into Asia via Beringia, followed by back-dispersal of Tyrannosaurini into Laramidia in the late Maastrichtian^9–11^. This hypothesis is based on the existence of stratigraphically earlier Asian taxa, *Tarbosaurus bataar*^12^ and *Zhuchengtyrannus magnus*^13^, which are close relatives of *T. rex*^9,10,14,15^. Alternatively, *Tyrannosaurus* has been hypothesized to represent an endemic, North American lineage^14^. Here, we describe NMMNH P-3698, a giant tyrannosaurid from the late Campanian—early Maastrichtian of New Mexico (Fig. 1). Although originally identified as *T. rex*^16–19^, this assignment has been questioned^20^. Restudy of the specimen, including newly recovered skeletal elements (Figs. 2, 3) confirm NMMNH P-3698 represents a distinct taxon, while recently published radiometric dates show that it predates *T*. *rex* by up to 7 million years. Phylogenetic analysis recovers NMMNH P-3698 as the closest known relative of *T. rex*, suggesting giant Tyrannosaurini evolved in southern Laramidia.

## Systematic paleontology

Dinosauria—Owen, 1842.

Theropoda—Marsh, 1881.

Tetanurae—Gauthier, 1986.

Coelurosauria—von Huene, 1914.

Tyrannosauridae—Osborn, 1905.

Tyrannosaurinae—Currie, 2003.

Tyrannosaurini—Olshevsky, 1995.

*Tyrannosaurus mcraeensis* sp. nov.

LSID:

urn:lsid:zoobank.org:pub:F1658ACA-60DB-442E-AA04-015C050205BD.

(Tyrannosaurini is here defined as the last common ancestor of *Tarbosaurus baatar* and *Tyrannosaurus rex* and all its descendants).

### Etymology

The species name, *mcraeensis*, refers to the McRae Group of western New Mexico.

### Horizon and locality

Uppermost Campanian or lower Maastrichtian of the Hall Lake Formation, McRae Group, NMMNH locality 343, near Kettle Top Butte, Sierra County, New Mexico^21^ (Fig. 1A). The site lies 43 m above the base of the Hall Lake Formation. A tuff 33 m below the tyrannosaur site has a U/Pb age of 73.2 ± 0.7 Ma^22^ (Fig. 1B).

### Diagnosis

**Figure 4:**
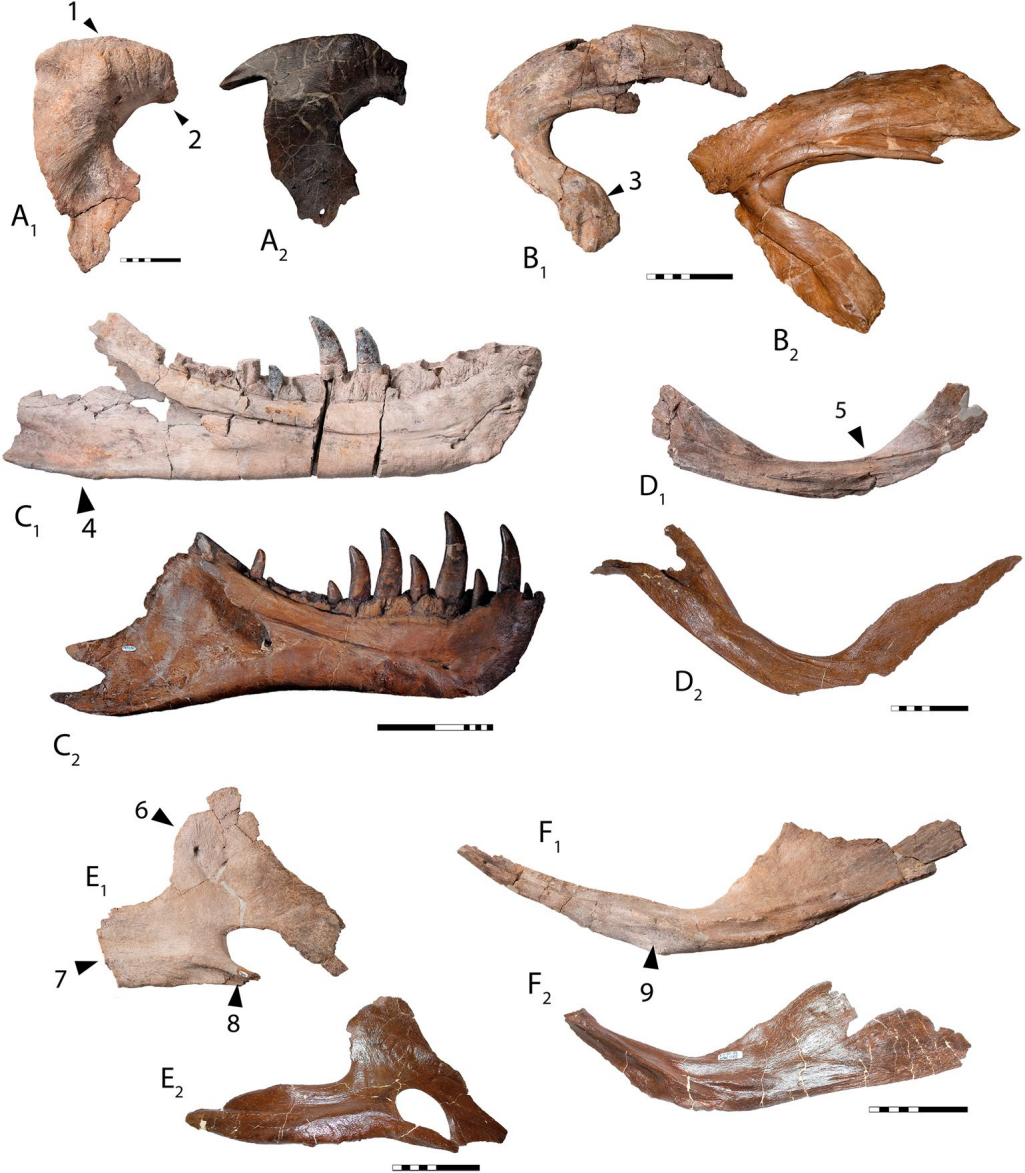
*Tyrannosaurus mcraeensis* compared with *Tyrannosaurus rex*. Postorbital of *Tyrannosaurus mcraeensis* (**A**_**1**_) and *Tyrannosaurus rex* (**A**_**2**_); Squamosal of (**B**_**1**_) *Tyrannosaurus mcraeensis* and (**B**_**2**_) *Tyrannosaurus mcraeensis*; Postorbital of (**C**_**1**_) *Tyrannosaurus mcraeensis* and (**C**_**2**_) *Tyrannosaurus rex*; prearticular of (**D**_**1**_) *Tyrannosaurus mcraeensis* and (**D**_**2**_) *Tyrannosaurus rex*; splenial of (**E**_**1**_) *Tyrannosaurus mcraeensis* and (**E**_**2**_) *Tyrannosaurus rex*; angular of (**F**_**1**_), *Tyrannosaurus mcraeensis* and (**F**_**2**_), *Tyrannosaurus rex*. Diagnostic characters: 1, low cornual process behind orbit; 2, anteriorly projected frontal/prefrontal articulation; 3, ventrally projected quadratojugal process; 4, upturned posteroventral margin of dentary; 5, weakly bowed prearticular; 6, anteriorly positioned splenial apex; 7, deep and posteriorly projected angular process; 8, shelf-like articulation overhanging dentary; 9, small splenial facet. Scale bars = 10 cm (**A**, **B**, **D**–**F**), 20 cm (**C**).

Large tyrannosaurin distinguished from *Tyrannosaurus rex* (Fig. 4; SI) by the following characters (*autapomorphies): postorbital with low, posteriorly positioned cornual process; postorbital with anteriorly projecting prefrontal/frontal articular surfaces; squamosal with ventrally projecting quadratojugal process; squamosal with a concave medial margin; strong ridge bounding the anterior margin of the squamosal ventral pneumatic fossa; dentary very shallow posteriorly and with a convex posteroventral margin*; splenial with anteriorly positioned apex*; splenial with shelf-like dentary overlap*, splenial with deep, posteriorly directed angular process; prearticular weakly bowed*; small ventral prearticular-angular contact; articular T-shaped in dorsal/ventral view; retroarticular process deep and quadrangular in posterior view.

### Holotype

NMMNH P-3698, partial skull including right postorbital and squamosal (Fig. 2), left palatine, fragment of maxilla, and lower jaws (Fig. 3) including left dentary, right splenial, prearticular, angular and articular, isolated teeth and associated chevrons.

### Description and comparisons

The postorbital of *Tyrannosaurus mcraeensis* (Fig. 2A–C) is typical of tyrannosaurines^5,12^ in bearing a massive cornual boss projecting above the postorbital dorsal margin. The cornual boss is C-shaped as in *Tarbosaurus*^12^ but lacks the strong anteroventral expansion or undercut margin seen in *Tarbosaurus* and *T. rex*. The cornual boss lacks the prominent, anteriorly positioned apex above the orbit seen in *T. rex*, instead being more posteriorly positioned. The postorbital’s dorsal margin is arched, as in *T. rex*^5^. In dorsal view the orbital margin and the body of the postorbital form a wide angle, indicating a posteriorly expanded skull as in *Lythronax*^14^, *Tarbosaurus*^12^, and *Tyrannosaurus rex*^5^. The frontal/prefrontal suture projects forward, whereas it is downturned in *T. rex* (Fig. 4A). The jugal process is anteroposteriorly expanded, with a convex jugal contact, as in *T. rex*^5^ and *T. bataar*^12^. A large suborbital process projects forward to constrict the orbit as in *Bistahieversor*^23^, *Teratophoneus*^14^, and especially *T. rex*^5^ and *Tarbosaurus*^12^.

The squamosal (Fig. 2D–F) recalls *Tyrannosaurus rex*^5^ in being elongate in dorsal/ventral view. It is shorter in *Tarbosaurus*^12^, and especially Albertosaurinae^24^. It differs from the *T. rex* holotype, where the squamosal is straight (Fig. 4B) in having a strongly downturned end in lateral view; however some specimens of *T. rex* show this feature (SI1). The quadratojugal process is downturned relative to *T. rex*^5^ or *Tarbosaurus*^12^ and strongly curved. Ventrally, the squamosal bears a deep pneumatic recess. The recess’ anterior margin is defined by a transverse bar as in *Tarbosaurus*; this bar is reduced to a low ridge in *T. rex*^12^.

The palatine (SI5) is broad, similar to *Tarbosaurus bataar*^12^ but not to the degree seen in *Tyrannosaurus rex*; the palatine of Albertosaurinae is narrower^24^. This wide palatine contributes to the formation of a broad rostrum as in *T. rex* and *Tarbosaurus*.

**Figure 5:**
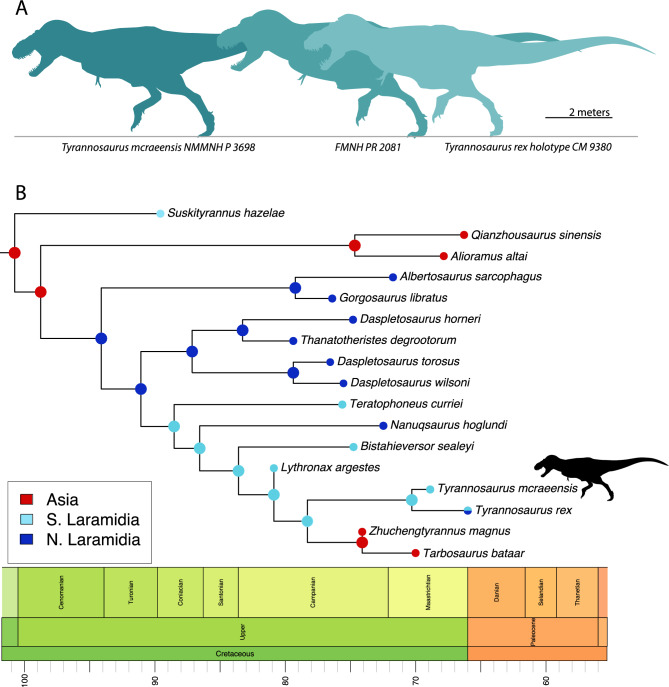
Size, relationships and biogeography of *Tyrannosaurus mcraeensis*. (**A**), relative sizes of *Tyrannosaurus mcraeensis* (NMMNH P-3698) and *Tyrannosaurus rex* (FMNH PR 2081 and CM 9380) (after^31^); (**B**), evolutionary tree based on Bayesian tip-dated phylogeny and biogeographic analysis (see SI for full results).

The dentary (Fig. 3A–C) measures 645 mm from the tip back to the level of the coronoid process; the bone measures 894 mm along the long axis as preserved, however the posteroventral end is broken; it may have measured around 900 mm when complete. By comparison, corresponding measurements for the holotype of *Tyrannosaurus rex* are 589 and 855 mm; RSM P2523.8, one of if not the largest known *T. rex* specimens^25^, measures ~ 650 from the tip of the dentary to the coronoid process. *Tyrannosaurus mcraeensis* therefore overlaps *T. rex* in size (Fig. 5A), although the holotype is smaller than the largest known *T. rex* individuals. The dentary has 13 alveoli, a low tooth count uniquely shared with *T. rex*^5^ which typically has 13 dentary alveoli, and rarely 14 or 15^26^ alveoli. *Zhuchengtyrannus* has 15 teeth, *Tarbosaurus* has 14–15^12,13^; *Daspletosaurus horneri*^10^ and *D*. *torosus* have 17. The first alveolus is smaller than the second, as in other tyrannosaurins. The symphysis is deep, being about 125% the depth of the dentary at mid-length, as in *T. rex*. The symphysis’ anteroventral margin rises up steeply, creating a squared-off chin, again as in *T. rex*. The dentary’s occlusal margin rises up steeply at the back forming a strongly concave dorsal margin, as in other tyrannosaurines^5,12–14^. Unusually, the dentary’s posterior end is shallow and upturned; it is deep and typically has a downturned ventral margin in *T. rex* and other tyrannosaurids^5,14,24^. This condition is unique to *T. mcraeensis* but approached in *Tarbosaurus*^5,12^ and *Zhuchengtyrannus*^13^. The angular process projects posteriorly, as in *Tarbosaurus* and *Zhuchengtyrannus*^13^ but unlike *T. rex* and other tyrannosaurids, where it projects posteroventrally^5,14,24^.

In dorsal view the tip of the dentary bows outward so that the symphysis forms a broad “U”. Similar bowing is present in *Tyrannosaurus rex*^5,14^ and *Tarbosaurus*, associated with transverse expansion of the rostrum. The dentary tip is straight in *Daspletosaurus*, *Lythronax*^14^, *Nanuqsaurus*^27^ and more basal taxa^28^. The dentary and toothrow curve outwards posteriorly, again suggesting transverse expansion of the skull’s postorbital region, as in *Lythronax*^14^, *Tarbosaurus*, and *T. rex*^5,12^.

Medially, interdental plates are large and rectangular to triangular, as in other tyrannosaurins^5,13^; they are smaller in Albertosaurinae^24^ and *Daspletosaurus*^24^. The lingual bar covers the first two alveoli. It is deep anteriorly then narrows posteriorly to half its anterior depth, a condition otherwise seen only in *Tyrannosaurus rex*^5^. The symphysis is rugose and covered with bumps and grooves, as in other tyrannosaurines. It extends back to the third alveolus, as in *Tarbosaurus*^12^. In *Zhuchengtyrannus*^13^ and some individuals of *T. rex*^5^ it ends under the fourth alveolus. The symphysis lies above the dentary ventral margin, as in *Tarbosaurus*^12^ and *Zhuchengtyrannus*^13^; in *T. rex* the symphysis projects below the dentary’s ventral margin^25^ in some, but not all individuals. The bone’s lateral surface bears pits and scars suggestive of intraspecific combat.

The triangular splenial (Fig. 3D) resembles other tyrannosaurids^5,12,24^. The tall dorsal process resembles *T. rex*^12^ and *Tarbosaurus*^12^; *Daspletosaurus*^24^ has a low dorsal process. The apex is triangular, like *Tarbosaurus*; in *T. rex* the apex is usually quadrangular (Fig. 4e). The apex is anteriorly displaced compared to other tyrannosaurids. The ventral margin is straight, and the angular process projects posteriorly, versus posteroventrally in *Tarbosaurus*^12^ and *Tyrannosaurus* *rex* (Fig. 4E). The angular process is deep, as in *Tarbosaurus*; it is shallow in *T. rex*^5^. The anteroventral process, beneath the mylohyoid foramen, has a shelf overlapping the dentary, in *T. rex* it abuts the dentary.

The anterior process of the angular (Fig. 3E) is longer and narrower than in *Tyrannosaurus rex* (Fig. 4F) and more similar to *Lythronax* and *Tarbosaurus*. It has a narrow ventral prearticular contact; *T. rex* has a broad anteroventral flange here. The ventral margin forms a sharp angle between the anterior and posterior processes.

The prearticular (Fig. 3F) is gently curved, versus strongly bent in *Tyrannosaurus rex*^5^ (Fig. 4D) and other tyrannosaurids^12,24^. The articular is T-shaped in dorsal view, versus more triangular in *T. rex*. In posterior view, the retroarticular process is deep and subrectangular; that of *T. rex* is wider, and semicircular (SI).

Teeth (Fig. 3; SI) resemble *Tyrannosaurus rex*^5^ in being large, robust, and labiolingually expanded. Tooth crowns have massive apices, as in *Tarbosaurus*^12^; *T. rex* teeth have more pointed, spike-like apices^5^. The apex of the sixth dentary tooth is worn; *T. rex* tooth wear is often heavy as a result of biting bone^6,29,30^. The labiolingual width of anterior teeth approaches their mesiodistal diameter, as in *T. rex*; posterior teeth are robust, but more laterally compressed. Both carinae bear serrations.

### Phylogenetic analysis

Phylogenetic analyses consistently recover *Tyrannosaurus mcraeensis* as sister to *T. rex* (Fig. 5) using either a parsimony-based or Bayesian tip-dated analysis (SI). The two share a reduced tooth count, deep dentary symphysis, posteriorly shallow lingual bar, and large size. *T. mcraeensis* is furthermore united with the *Tarbosaurus* + *Tyrannosaurus* clade by a postorbital with a broad ventral ramus, strongly convex posterior margin, and large suborbital flange, by the strongly concave dentary alveolar margin, and by the laterally bowed mandible.

## Discussion

### Systematics

**Figure 6:**
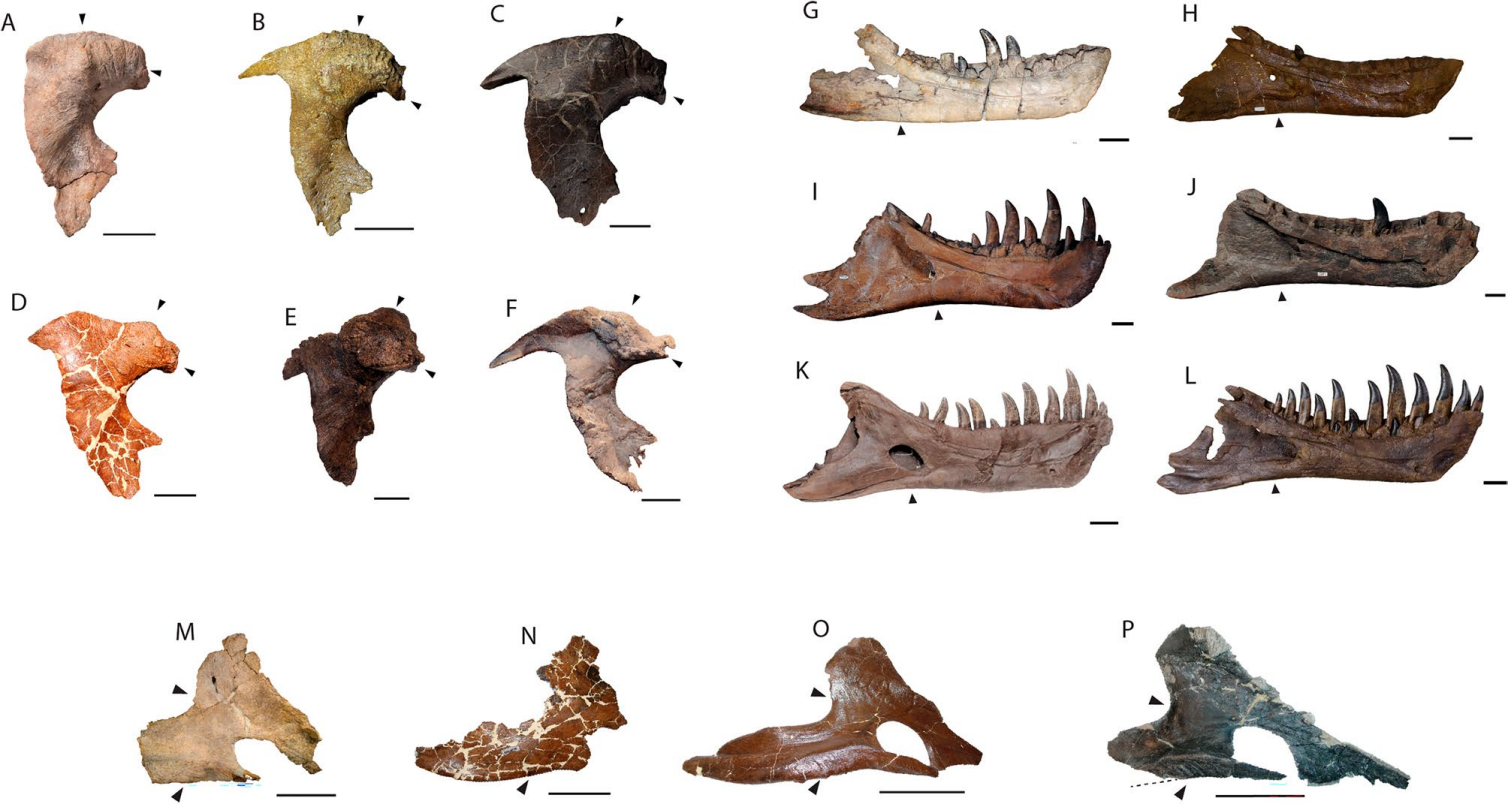
Variation in the postorbitals (**A**–**F**), dentaries (**G**–**K**) and splenials (**M**–**Q**) of *Tyrannosaurus mcraeensis* (**A**, **G**, **M**) and *Tyrannosaurus rex* (**B**–**F**, **H**–**L**, **N**–**Q**). See SI for specimen numbers. Scale bars = 10 cm.

NMMNH P-3698 was referred to *Tyrannosaurus rex*^17,19,21^ based on size and overall resemblance to *T. rex*, but this assignment has been questioned^20^. Newly collected material shows that *T. mcraeensis* differs from *T. rex* in the shape of the postorbital, squamosal, dentary, prearticular, angular, and articular. The characters that diagnose *T. mcraeensis* and differentiate it from *T. rex* are relatively subtle characters relating to the shape and articulation of the skull bones, but because *T. rex* is known from multiple individuals, it is possible to show that *T. mcraeensis* lies outside of the range of individual variation seen in *T. rex* (Fig. 6).

*Tyrannosaurus rex* exhibits a high degree of variation^31–33^ and specimens assigned to the genus may or may not represent multiple species^31,33,34^. However in each of the diagnostic characters identified here, NMMNH P-3698 is unlike any other specimen referred to *T. rex*; furthermore each bone has at least one diagnostic character, it is therefore an outlier from all other specimens referred to *T. rex*, in every element. Neither can the differences between *T*. *rex* and *T. mcraeensis* be explained as ontogenetic, given that the animal matched *T*. *rex* in size. It is worth noting that these differences are subtle, but the differences between species are often relatively subtle. Characters diagnosing different species of *Daspletosaurus*^10,15^ tend to be relatively subtle, as are those diagnosing different species of *Alioramus*^35^.

### Age of NMMNH P-3698

Referral of NMMNH P-3698 to *Tyrannosaurus rex* was also based on the assumption that the Hall Lake Formation is late Maastrichtian aged; but no geological or paleontological evidence supports this assignment. In fact, one of the main arguments for a late Maastrichtian age was the incorrect assumption that Hall Lake dinosaurs could be referred to the late Maastrichtian taxa *Torosaurus* and *Tyrannosaurus rex*^19^. The ceratopsid previously referred to *Torosaurus* is now identified as a distinct genus, *Sierraceratops*^16^; as shown here, NMMNH P-3698 is distinct from *T. rex*.

The dinosaur fauna suggests a latest Campanian or Maastrichtian age. *Sierraceratops* is similar to species known from the latest Campanian, including *Coahuilaceratops magnacuerna*^36^, and to *Bravoceratops polyphemus* from the Javelina Formation of Texas^37,^ which is probably either latest Campanian or early Maastrichtian. The existence of a titanosaurian sauropod in the Hall Lake Formation has been cited evidence of a late Maastrichtian or ‘Lancian’ age^19,38^. Titanosaurs appear suddenly in the latest Cretaceous of the American Southwest, suggesting a distinct biogeographic event resulting from their sudden dispersal into North America^39^; sauropods are therefore a useful biostratigraphic constraint. However, titanosaurs range from just beneath the K-Pg boundary^40^ to at least 69 ± 0.9 Ma, i.e. mid-Maastrichtian^40^. Sauropods are unknown from the De-Na-Zin Member of the Kirtland Formation of New Mexico^39^, the top of which is dated to 73.5 Ma^41^, or the Cerro Del Pueblo Formation of Mexico, which is latest Campanian, ~ 73.5–73 Ma^42^. Sauropods therefore appear to immigrate into North America between 73 and 69 Ma. The existence of titanosaurs in the assemblage, therefore, does not reject a latest Campanian or early Maastrichtian age, and given that the sauropod appears higher in section, NMMNH P-3698 could predate the arrival of titanosaurians in North America. Finally, a large hadrosaur femur resembles that of the Campanian-Maastrichtian^42^ *Edmontosaurus*^43^ and a large edmontosaurin from the latest Campanian Cerro Del Pueblo Formation^44^ in terms of its large size and strongly inturned proximal femoral shaft. The presence of an edmontosaurin is consistent with an age range of latest Campanian to latest Maastrichtian. The dinosaur fauna as a whole is consistent with and suggests either a Campanian to Maastrichtian age. The ceratopsid *Sierraceratops* also tends to suggest a latest Campanian to early Maastrichtian age. Strikingly, no diagnostic late Maastrichtian dinosaur species are known.

**Figure 7:**
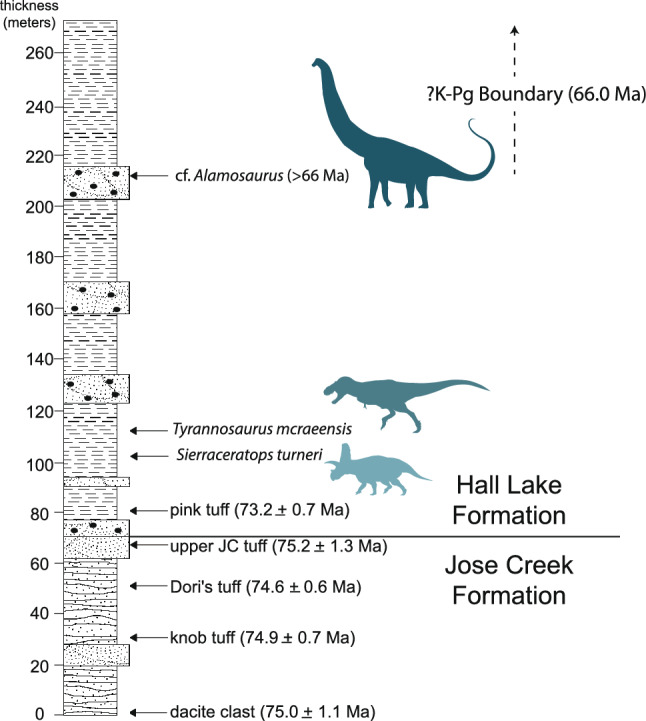
Stratigraphy and radiometric dates of the basal Hall Lake Formation and top of the underlying Jose Creek Formation.

Morphology also suggests *Tyrannosaurus mcraeensis* is older than *T*. *rex*, since it consistently lacks many derived characters characterizing *T. rex*. Tip-dated phylogenetic analysis recovers NMMNH P-3698 at 69 Ma, slightly younger than implied by the radiometric dates, but still much older than any known *T. rex* (Fig. 7).

Radiometric dates also point towards a latest Campanian or early Maastrichtian age. U/Pb dating^22^ gives an age of 73.2 ± 0.7 Ma for a tuff 9 m above the base of the Hall Lake Formation, and a series of Late Campanian ages for the upper 70 m of the immediately underlying Jose Creek Formation, at 75.2 ± 1.3 Ma, 74.6 ± 0.6 Ma, 74.9 ± 0.7 Ma, and 75.0 ± 1.1 Ma. Although no radiometric dates are found above NMMNH P-3698, a titanosaur is found 108 m above the site, and can be no younger than 66 Ma, i.e. the K-Pg boundary. No hiatus, coal, or other evidence of the K-Pg boundary is visible in the exposed section, suggesting around 150 m of Cretaceous rock overlay the site. Depending on the estimated rate of sedimentation, *Tyrannosaurus mcraeensis* may therefore have lived between 72.7 and 70.9 Ma (SI), i.e., latest Campanian or earliest Maastrichtian, 5–7 million years before *Tyrannosaurus rex*.

Overall, three independent lines of evidence—(i) the dinosaur fauna, (ii) the morphology of NMMNH P-3698 itself, and (iii) the age constraints provided by underlying radiometric dates and overlying dinosaur bones suggest an age somewhat older than the latest Maastrichtian, and that the animal predates *Tyrannosaurus rex*. However, additional radiometric dates, palynostratigraphy, or vertebrate fossils are needed to provide more precise constraints on the age of NMMNH P-3698.

*Tyrannosaurus mcraeensis* shows several derived characters *T. rex* does not: an upturned dentary posteroventral margin, a weakly curved prearticular, and an anterior splenial apex (Fig. 4). These characters imply that *T. mcraeensis* was a side-branch in tyrannosaurin evolution, and would not have been directly ancestral to *Tyrannosaurus rex*. If so, then at least two giant tyrannosaurids existed concurrently in North America, with another species ultimately giving rise to *Tyrannosaurus rex*.

### Biogeography and the evolution of body size

**Figure 8:**
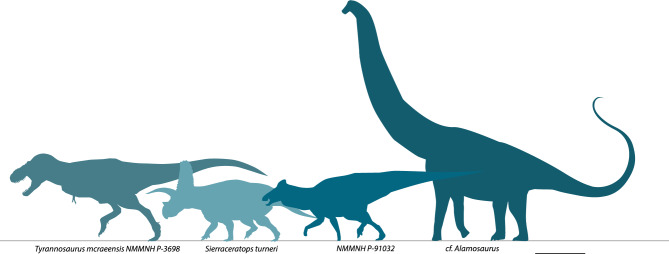
Dinosaurs of the Campanian–Maastrichtian Hall Lake Formation. Scale = 2 m.

*Tyrannosaurus mcraeensis* belonged to an endemic southern dinosaur community. The Hall Lake fauna (Fig. 8) included *T. mcraeensis*, the giant chasmosaur *Sierraceratops*^16^, the titanosaurian cf. *Alamosaurus*, and a giant hadrosaurid^19^. This fauna shows no overlap with penecontemporaneous faunas in the latest Campanian/early Maastrichtian of Canada. This northern fauna included the albertosaurine tyrannosaurid *Albertosaurus*, the centrosaurine *Pachyrhinosaurus*, the lambeosaur *Hypacrosaurus*, the hadrosaurs *Edmontosaurus* and *Saurolophus*, and the chasmosaurine *Anchiceratops*; sauropods are absent^45^. Discovery of *T. mcraeensis,* therefore, corroborates hypotheses that Laramidian faunas were characterized by high endemicity^16,46–48^, with the Southwest supporting distinct dinosaur species and clades (Fig. 8).

The presence of *Tyrannosaurus mcraeensis* in New Mexico and its phylogenetic position suggest that Tyrannosaurini^14^ originated as part of this southern fauna. A southern origin of Tyrannosaurini is further supported by the presence of cf. *Tyrannosaurus* in the Javelina Formation of Texas^49,50^ and *T. rex* in the latest Maastrichtian North Horn of Utah^7^. Following the appearance of tyrannosaurins in southern Laramidia during the Campanian, one lineage dispersed to Asia, giving rise to *Tarbosaurus* and *Zhuchengtyrannus*; another gave rise to *Tyrannosaurus*, eventually moving north to displace albertosaurines in the Late Maastrichtian.

In an alternative character-matrix^51^, *Tyrannosaurus mcraeensis* again emerges as sister to *T*. *rex* (SI). In this topology, *Tarbosaurus* and *Zhuchengtyrannus* are successive outgroups to the *T. mcraeensis*–*T. rex* clade. This topology is ambiguous about the origin of Tyrannosaurini, consistent with either two dispersals of Tyrannosaurinae into Asia, or dispersal into Asia, followed by back-dispersal into North America. The difference is due to the poorly known *Zhuchengtyrannus* acting as a wildcard taxon, clustering either with *Tarbosaurus*^14^ or as sister to the *T*. *rex*—*Tarbosaurus* clade^9,10,15^. The out-of-Asia scenario is dependent on the placement of this uncertain taxon. Resolving these ambiguities requires a better understanding of Asian tyrannosaurs. Similarly, placement of *Daspletosaurus* spp. along the line leading to Tyrannosaurini^15^ would tend to support a northern origin of the Tyrannosaurini. Further study of southern tyrannosaurs such as *Bistahieversor* and *Lythronax* can potentially resolve these issues, along with revisions of tyrannosaur phylogeny, but we argue that the appearance of *T*. *rex*-like dinosaurs in the Javelina Formation, which appear to be slightly older than *T*. *rex* itself, better fits the southern scenario^9,50^*.*

*Tyrannosaurus mcraeensis* shows that Tyrannosaurini achieved giant size near the end of the Campanian. It furthermore suggests that they did so in southern Laramidia (Fig. 5) but were initially restricted to southern Laramidia, and later dispersed north^7^. Chasmosaurinae show similar patterns, with giant Triceratopsini evolving in southern Laramidia in the Campanian, then moving into the northern Great Plains in the Maastrichtian^46^. Several other giant dinosaurs evolved in southern Laramidia, including large kritosaurin hadrosaurs^52^, the giant lambeosaurine *Magnapaulia laticaudus*^53^, and the titanosaurian *Alamosaurus sanjuanensis*^54^, but did not migrate north. Meanwhile, a tyrannosaurid from northernmost Laramidia, *Nanuqsaurus hoglandi*^27^, was smaller than tyrannosaurines from farther south. This, and the presence of giant ceratopsians and hadrosaurs in the Hall Lake Formation (SI) implies geographic patterns of body size evolution: large dinosaurs evolved in southern Laramidia.

Sea level changes have been hypothesized to drive tyrannosaurid diversification^14^, and among mammals, land area was found to be associated with body mass of top carnivores and herbivores^55^. However, the patterns raised here suggest that other processes are at work in driving tyrannosaurid size evolution. The evolution of *Tyrannosaurus*-sized tyrannosaurs appears to precede end-Maastrichtian marine regression by several million years; furthermore the giant tyrannosaurs appear to be initially restricted to a limted area of the relatively small Laramidian landmass. Gigantism therefore appears not to be driven by increases in land area, instead giant theropods evolved despite having relatively small geographic ranges.

Giant tyrannosaurins likely evolved to prey on the giant herbivores found in the south, although the reason for the evolution of large herbivorous dinosaurs—possibly including latitudinal variation in mean annual temperature, primary productivity, or seasonality—remains unknown. These patterns also suggest caution in relying on local dinosaur diversity patterns to study global dinosaur diversity changes preceding the K-Pg extinction.

## Materials and methods

We added *Tyrannosaurus mcraeensis* to an existing character-taxon matrix^14^, adding 10 new characters as well as additional taxa (SI2). Phylogenetic analysis was implemented using cladistic methods and total-evidence estimation of phylogeny and divergence times using the fossilized clock model and birth–death priors under diversified sampling as described by recent authors^56^, with tip-dated estimates of fossil ages from all sampled taxa chosen using a “diversity” strategy to span internal branches. We optimized biogeographic history across the summary tree by classifying species into six areas: Europe, South America, Asia, Appalachia, Northern Laramidia, and Southern Laramidia, the latter two separated by present-day Utah and Colorado as the demarcating boundary. We tested a standard suite of biogeographic models in RASP^57^, selecting the DEC + J model as the best fit, and present the most-likely estimates for ancestral range (Fig. 5B).

### Supplementary Information

Supplementary Information 1.

Supplementary Information 2.

Supplementary Information 3.

Supplementary Information 4.

## Data Availability

All data is available in the manuscript or Supporting Information.
