# Supplementary material for: A giant tyrannosaur from the Campanian–Maastrichtian of southern North America and the evolution of tyrannosaurid gigantism
Source: Sci Rep. 2024 Jan 11;14:22124. doi: 10.1038/s41598-023-47011-0 (PMC10784284; doi:10.1038/s41598-023-47011-0)
Supplement: Supplementary file 1 — Supplementary Information 1. [file 41598_2023_47011_MOESM1_ESM.docx]

**A giant tyrannosaur from the Campanian-Maastrichtian of southern North America and the evolution of tyrannosaurid gigantism**

**Sebastian G. Dalman^1^, Mark A. Loewen^2,3^, R. Alexander Pyron^4^, Steven E. Jasinski^5^, D. Edward Malinzak^6^, Spencer G. Lucas^1^, Anthony R. Fiorillo^1^, Philip J. Currie^7^, and Nicholas R. Longrich^8*^**

^1^New Mexico Museum of Natural History and Science, 1801 Mountain Road N.W., Albuquerque, NM, 87104, USA; ^2^Department of Geology and Geophysics, University of Utah, Salt Lake City, UT, USA; ^3^Natural History Museum of Utah, University of Utah, Salt Lake City, UT, USA.; ^4^Department of Biological Sciences, The George Washington University, 2023 G St. NW, Washington, DC 20052, USA; ^5^Department of Environmental Science and Sustainability, Harrisburg University, 326 Market Street, Harrisburg, PA, 17101, USA; ^6^Penn State Lehigh Valley, Center Valley, PA 18034, USA ^7^Department of Biological Sciences, University of Alberta, Edmonton, Alberta, T6G 2E9, Canada; ^8^Department of Biology and Biochemistry, University of Bath, BA2 7AY, United Kingdom.

**Contents**

1. **Chronostratigraphy**
2. **Extended Description**
3. **Extended Diagnosis**
4. **Phylogenetic Analysis**

**1. Chronostratigraphy**

*Locality*. NMMNH locality 343, Elephant Butte Reservoir, near Kettle Top Butte, Sierra County, New Mexico (Lucas et al., 2019).

*Stratigraphy*. NMMNH locality 343 lies near the base of the Late Cretaceous Hall Lake Formation, above the Jose Creek Formation, in the McRae Group. The McRae Group is more than 1 kilometer thick. The Hall Lake Formation (previously the Hall Lake Member of the McRae Formation) is a Late Cretaceous terrestrial section, consisting of at least 730 m of strata. Sediments are reddish brown to maroon mudstones and sandstone tongues, sometimes with conglomerates. NMMNH locality 343 lies 43 m above the base of the formation.


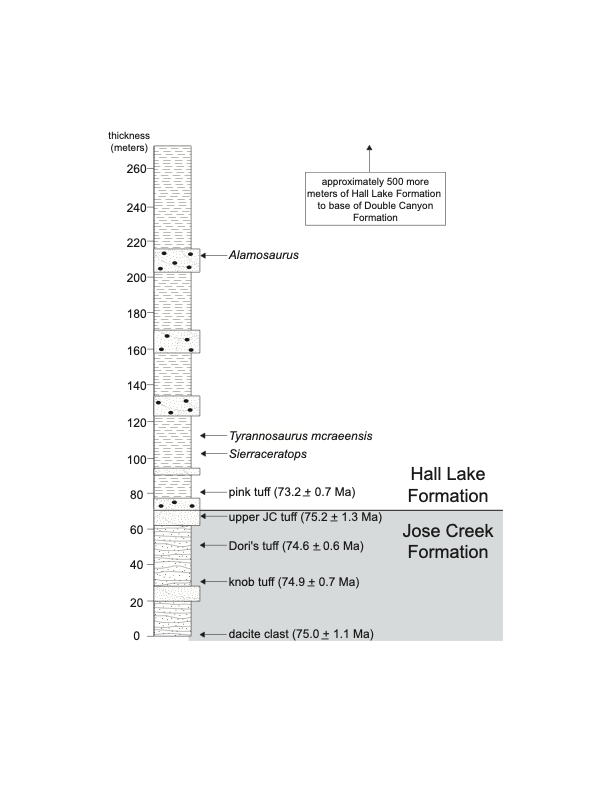


**Fig. S1.** Stratigraphy and radiometric dates of the basal Hall Lake Formation and top of the underlying Jose Creek Formation.

A tuff 10 meters above the base of the formation, and 33 m below the tyrannosaur site (NMMNH locality 343) itself has a U/Pb age of 73.2 ± 0.7 Ma (Amato et al., 2017), putting a maximum age of 73.9-72.5 Ma for *Tyrannosaurus mcraeensis* (Fig. S1). A sauropod femur lies 141 meters above the base of the formation, and 108 meters above NMMNH locality 343. Thus, the K/Pg boundary at 66 Ma lies at least 108 meters above NMMNH locality 343, and the 131 meters from the tuff to the femur span a maximum of 7.2 ± 0.7 million years.

1. Assuming a relatively constant rate of deposition, the 131 meter section here, spanning 7.2 ± 0.7 Ma, implies a depositional rate of 18.2 meters / Ma. This in turn equates to the 33 meters between the tuff and the site representing 1.81 Ma. This implies an age of **71.4 (71.9—70.9)** **Ma** for the tyrannosaur locality, or early Maastrichtian.
2. This age estimate could be biased downwards if the sandstones above the locality were deposited relatively quickly compared to the mudstones, as they would span relatively less time. On the other hand, the K-Pg boundary remains unidentified in the McRae Group and there is no evidence to believe that the highest dinosaur corresponds to the K-Pg boundary; that assumption likely biases this age estimate making it too young. The K-Pg boundary is often marked by an unconformity or change in deposition across the boundary, often recognized as a change in rock formations. For example, the late Maastrichtian Hell Creek Formation in Montana and the Lance Formation in Wyoming are capped by the basal Paleocene Fort Union Formation. In other areas of New Mexico, the Maastrichtian Naashoibito Member of the Ojo Alamo Formation is capped by the basal Paleocene Kimbeto Member of the Ojo Alamo Formation. There is no such transition observed in the Hall Lake Formation, implying that the entire section could be Cretaceous. Assuming the 730 m or so thickness is entirely Cretaceous in age, this would imply an average depositional rate of 100 m / Ma. If so, the tyrannosaur locality lies 0.33 Ma above the tuff, or an estimated **71.73 Ma**.
3. Several tuffs also lie in the 150 meters below the tyrannosaur locality. They are at 75.2 ±1.3 Ma, 74.6 ±0.6 Ma, 74.9 ± 0.7 Ma, and 75.0±1.1 Ma. Between the uppermost tuff and bottommost tuff there are 110 meters of sediment, and the dates span 1.8 Ma, implying a depositional rate of 61 meters / Ma. This suggests the tyrannosaur lies .54 Ma above the uppermost tuff, or at an estimated **72.66 Ma**.

These are not meant to be precise estimates of the age. Rather they suggest that using a variety of reasonable assumptions to estimate sedimentation rates *Tyrannosaurus mcraeensis*, it comes out between ~71 Ma and 73 Ma, suggesting a latest Campanian or earliest Maastrichtian age.

**SI Table 1.** Age approximations derived from different assumptions of sedimentation rates for the basal Hall Lake Formation.

| Assumed sedimentation Rate | *T. mcraeensis* age | Age |
| --- | --- | --- |
| 18.2 m / Ma | 71.4 Ma | Early Maastrichtian |
| 100 m / Ma | 71.73 Ma | Early Maastrichtian |
| 61 m / Ma | 72.66 Ma | Latest Campanian |

*Dinosaur Biostratigraphy and Associated Fauna*. The dinosaur fauna is consistent with either a late Campanian or early Maastrichtian age. The ceratopsid found in the fauna, *Sierraceratops turneri* (Dalman et al., 2022), is most similar to the species *Coahuilaceratops magnacuerna* from the Cerro del Puebla Formation, of Coahuila, Mexico (Loewen et al., 2010) and to *Bravoceratops polyphemus* from the Javelina Formation of Texas (Wick and Lehman, 2013). The Cerro Del Pueblo Formation is Campanian in age (Loewen et al., 2010). A tuff from 60 m above the base of the Javelina Formation is dated to 69 Ma, with *Bravoceratops* coming from 57 m below this tuff, putting it in the latest Campanian or early Maastrichtian (Wick and Lehman, 2013). Thus, *Bravoceratops,* at 76.28 ± 0.06 to 68.83 ± 0.13 Ma is consistent with either a late Campanian or early Maastrichtian age (Wick and Lehman, 2013; Lehman et al., 2017; Leslie et al., 2018).


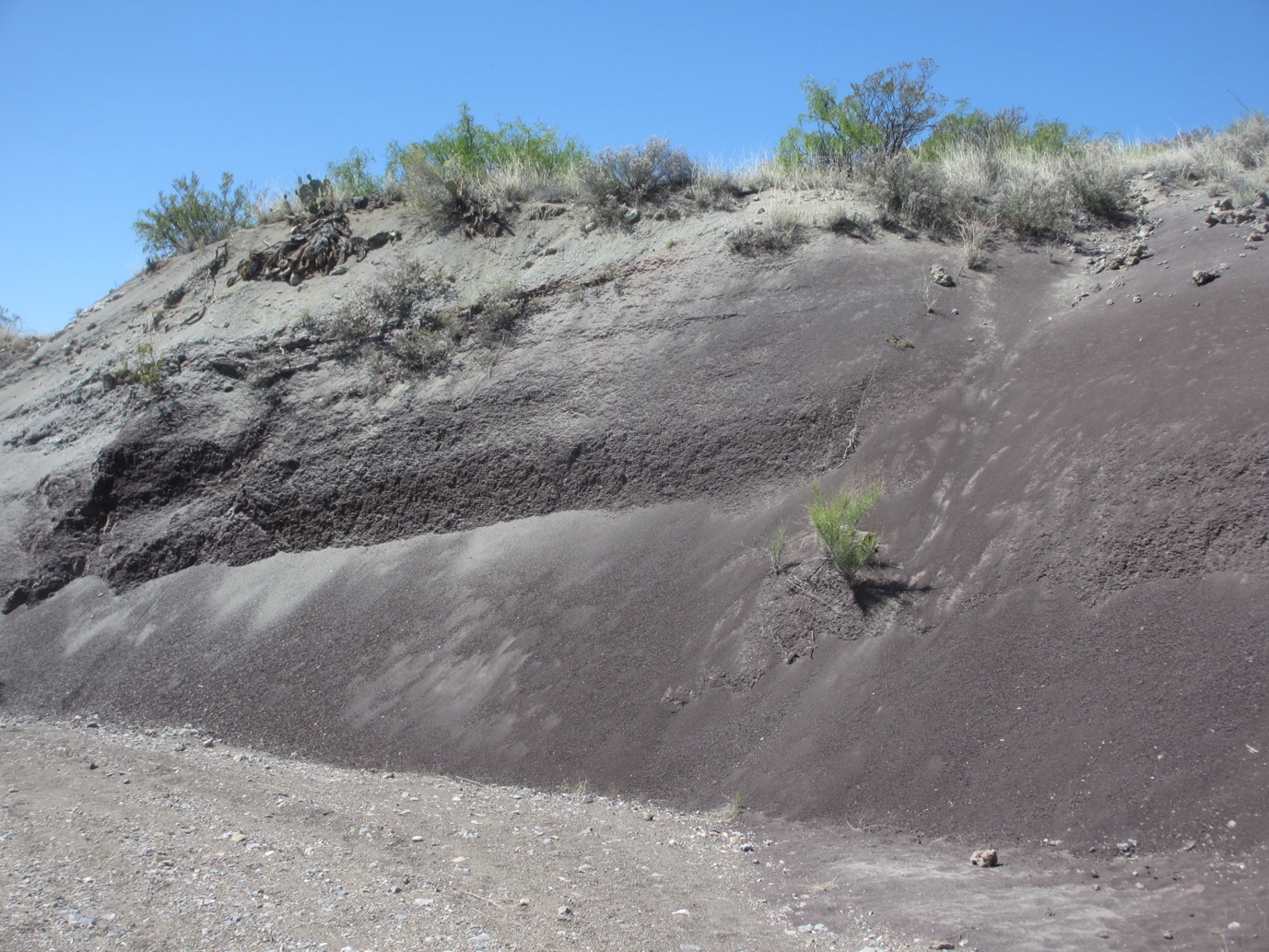


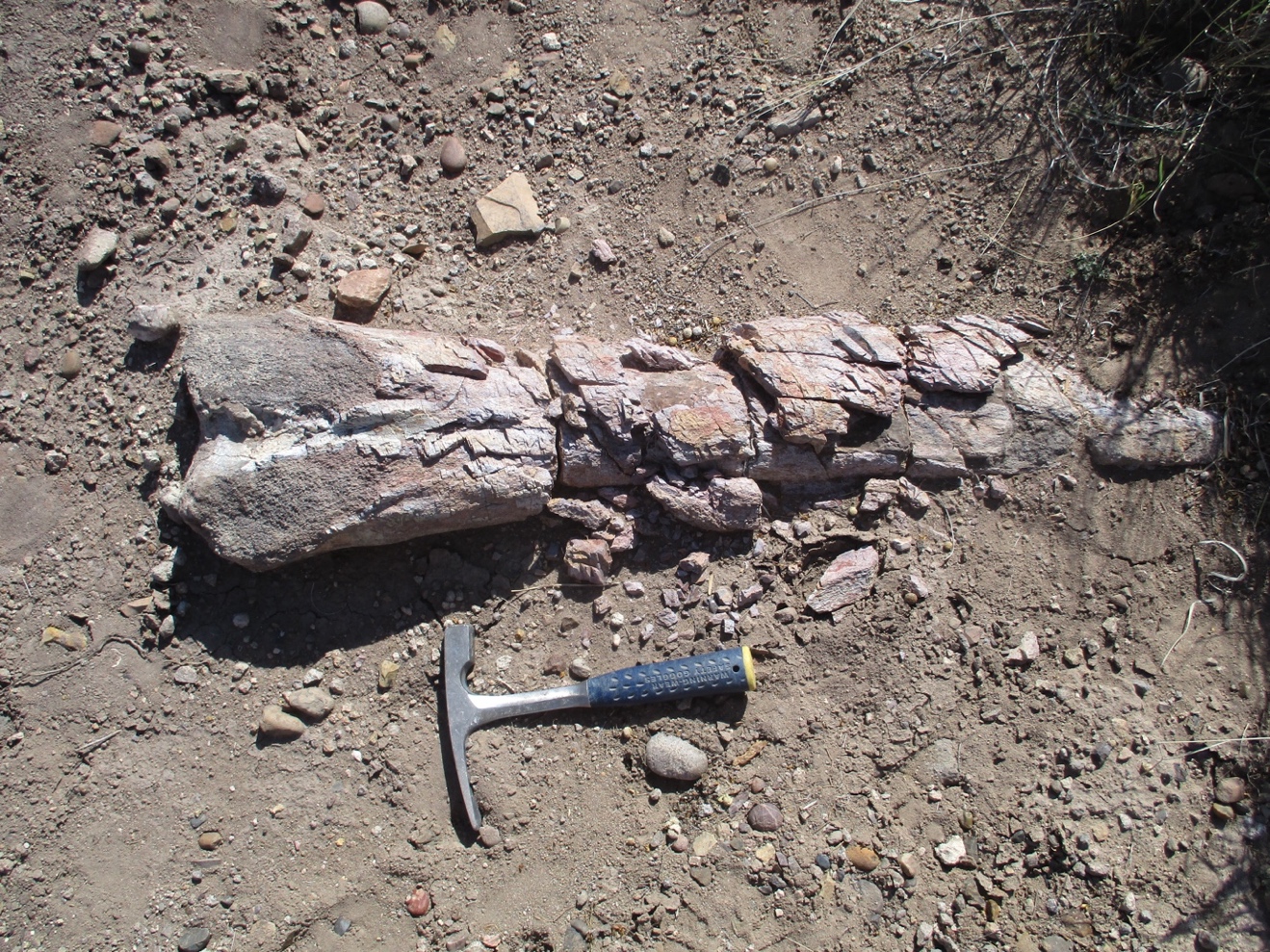


**SI Fig. 2.** Hall Lake Formation outcrops (top); hadrosaur femur in the Hall Lake (bottom)

A large hadrosaur is also present in the assemblage (Fig. S2), documented by the presence of a femur, NMMNH P-91032. The inturned shape of the femoral head resembles an unnamed large hadrosaur from the latest Campanian of the Cerro Del Pueblo Formation (Kirkland et al., 2006), which was originally thought to represent a kritosaurin, but has more recently been interpreted as a large edmontosaurin (Ramírez-Velasco, 2022). The New Mexico femur differs primarily in being more robust. Edmontosaurs are also known from the Maastrichtian (Campione and Evans, 2011). The hadrosaur fauna is therefore consistent with either a late Campanian or Maastrichtian age.

Finally, a titanosaurian sauropod is present in the Hall Lake Formation as well (Lucas et al., 2019). Titanosaurs appear in North America in the Maastrichtian. A titanosaurian comes from just under a tuff bed in the Javelina Formation that is dated to 68.83 ± 0.13 -66.0 Ma (Lehman et al., 2006; Leslie et al., 2018), and titanosaurians range up to the Cretaceous-Paleogene boundary in the Black Peaks Formation (Lehman et al., 2006; Leslie et al., 2018). The titanosaur found in the Hall Lake Formation is therefore suggestive of a mid-to-late Maastrichtian age. However, this specimen comes from ~ 100 meters above *Tyrannosaurus mcraeensis* (Lucas et al., 2019); so it is possible that the titanosaur is mid or late Maastrichtian, while the tyrannosaur is early Maastrichtian or even Late Campanian. The absence of titanosaurs lower stratigraphically is consistent with the idea that the lower Hall Lake predates their appearance in southwest Laramidia at 69 Ma, although further sampling could easily prove otherwise.

A mid-Maastrichtian or late Maastrichtian (i.e., ‘Lancian’) age cannot be definitively ruled out on the basis of the known dinosaurs. However, the absence of known ‘Lancian’ dinosaurs such as *Torosaurus*, *Triceratops*, *T*. *rex* and *Edmontosaurus* tends to suggest an older age.

The dinosaur fauna is overall most consistent with a latest Campanian to early Maastrichtian age for the Hall Lake fauna, possibly extending into the mid-Maastrichtian higher in section where sauropods appear, congruent with the evidence from radiometric dates.

The dorsal vertebrae of *Sierraceratops* are very large; the anterior faces of the three vertebrae associated with the type measure 157 mm wide by 170 mm tall, 162 mm wide by 189 mm tall, and 150 mm wide by 153 mm tall. A precise mass estimate of this animal is difficult given the absence of limb material, but the maximum diameter of these vertebrae approaches those of large *Triceratops* (189 mm versus 207 mm for *Triceratops* “*maximus*”) (Brown and Kaisen, 1933) suggesting that it approached the size of *Triceratops*; previous reconstructions of *Sierraceratops* (Dalman et al., 2022) underestimated its size.

The hadrosaur femur, NMMNH P-91032, measures 1235 mm in length but is extremely robust, with a circumference of 589 mm (545 without the fourth trochanter). Assuming a similar humerus circumference:femur circumference ratio to that of *Edmontosaurus regalis* (Benson et al., 2014) of 50.75%, the humeral circumference would be 299 mm. The combined humeral + femoral circumference of 888 mm implies a body mass of 10,025 kg using equation 2 of Campione and Evans (Campione and Evans, 2020) to 12,000 kg using equation 5 of Campione and Evans (Campione and Evans, 2020), making it one of the largest hadrosaurids known from North America.

The cf. *Alamosaurus* femur (NMMNH P-10789) measures 1300 mm long but is incomplete; complete it probably measured 1500 mm.

**2. Extended Description**

*Institutional Abbreviations*. AMNH, American Museum of Natural History, New York City; BHI, Black Hills Institute; CM, Carnegie Museum of Natural History, Pittsburgh; FMNH, Field Museum of Natural History, Chicago; LACM, Natural History Museum of Los Angeles, Los Angeles; MOR, Museum of the Rockies, Bozeman; NMMNH, New Mexico Museum of Natural History, Albuquerque; RSM, Royal Saskatchewan Museum, Eastend; TMP, Royal Tyrrell Museum of Paleontology, Drumheller.

**
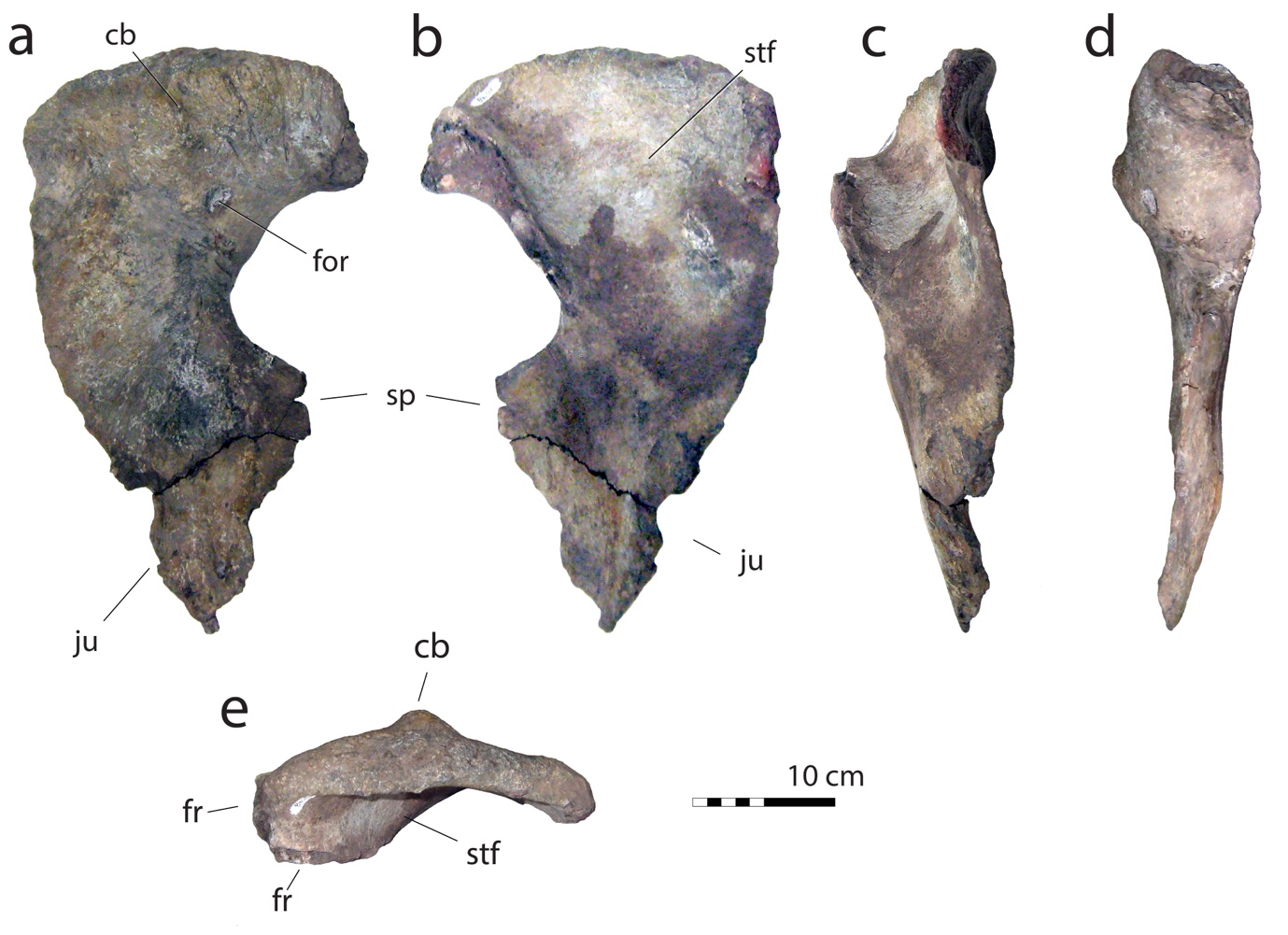
**

**Fig. S3.** Right postorbital of NMMNH P-3698 in **a**, lateral, **b** medial, **c**, posterior, **d**, anterior, **e**, dorsal views. Abbreviations: cb, cornual boss; fr, frontal articulation; for, foramen; ju, jugal contact. Scale = 10 cm.

**Postorbital.** The postorbital (Fig. S3) is typical of tyrannosaurines (Brochu, 2002; Hurum and Sabath, 2003) in being robust with a prominent bump-like cornual process. The dorsal margin is arched, as in *T. rex* (Brochu, 2002).

The anterior frontal contact projects anteriorly, rather than being downturned as in *T. rex*. In dorsal view, there is a very broad angle between the orbital margin for the frontal and the lateral margin, similar to *T. rex* and *Tarbosaurus bataar*, and suggesting a relatively broad posterior skull. This angle is more acute in more basally diverging taxa such as *Gorgosaurus libratus*, where the posterior skull is not expanded to the same degree (Currie, 2003).

**
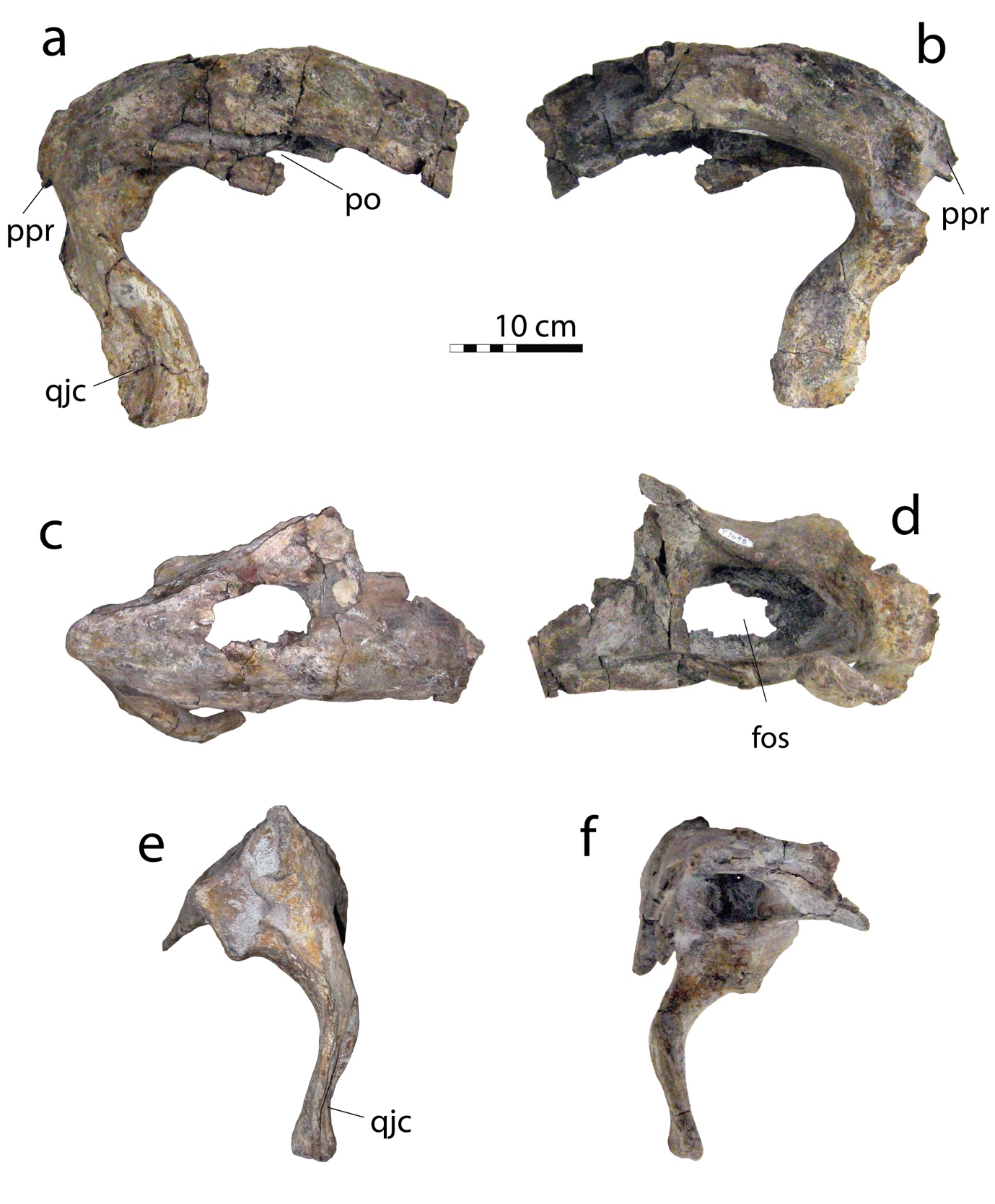
**

**Fig. S4.** Right squamosal of NMMNH P-3698 in **a**, lateral, **b** medial, **c**, dorsal, **d**, ventral, **e**, posterior; **f**, anterior views. Abbreviations: fos, ventral fossa / recess; po, articulation for postorbital; posterior, paroccipital process; qjc, quadratojugal contact. Scale = 10 cm.

The cornual boss is bump-like, similar to other tyrannosaurines (Currie, 2003), especially *Tarbosaurus*. The cornual boss is smaller than in *Tyrannosaurus rex* (Brochu, 2002) and lacks the prominent apex above the orbit seen in *T. rex* . The postorbital bears a large foramen posterodorsal to the orbit; this foramen is absent in *T. rex* (Brochu, 2002) and other tyrannosaurines. It may be a pneumatic opening, or a pathology. The postorbital ventral ramus is anteroposteriorly broad, with a strongly convex posteroventral margin where it contacted the jugal, as in *T. rex* (Brochu, 2002), *T. bataar* (Hurum and Sabath, 2003), and *Bistahieversor sealeyi* (Carr and Williamson, 2010). A large, anteriorly-projecting suborbital flange projects forward from the ventral process and bounds the orbit as in *T. rex* (Brochu, 2002) and *T.* *bataar* (Hurum and Sabath, 2003). The medial surface of the postorbital has a concave fossa where the anterodorsal ramus, posterior process, and ventral ramus meet. The postorbital has a broad, bipartite contact with the frontal formed by an anterior and posterior facet, as in other tyrannosaurids (Brusatte et al., 2012; Voris et al., 2019). The posterior contact is damaged.

**Squamosal.** The body of the squamosal (Fig. S4) resembles that of *Tyrannosaurus rex* (Brochu, 2002) in being long and narrow in dorsal/ventral view. It is shorter relative to its width in *Tarbosaurus bataar* (Hurum and Sabath, 2003), and especially Albertosaurinae (Currie, 2003). The squamosal’s ventral recess broadly excavates the entire ventral surface. The recess’s margins are well defined, with the anterior margin wider than the posterior margin. Anterior to the recess, a plate-like structure joins the anterior and medial processes; similar plate occurs in *T. bataar* (Hurum and Sabath, 2003) and *T. rex* (Brochu, 2002). In *T. mcraeensis* the plate’s lateral and medial expansions are greater, especially medially, and far greater than in *T. rex*.

The squamosal has four processes: an anterior, ventral, posterior, and a medial process. The anterior process is deep in lateral view, but shallower than in *Daspletosaurus horneri* (Carr et al., 2017), *Tarbosaurus bataar* (Hurum and Sabath, 2003) or *Tyrannosaurus rex* (Brochu, 2002). In lateral view anterior process is strongly bowed relative to the body of the squamosal. This feature differs from the holotype of *T*. *rex*, and a specimen of *T. rex* from C29r of the Frenchman Formation (Persons IV et al., 2020). However, in this feature *Tyrannosaurus mcraeensis* resembles *Tyrannosaurus* FMNH PR 2081. FMNH PR 2081 has been proposed to represent a distinct taxon, *T. imperator* (Paul et al., 2022), although this reclassification remains controversial (Carr et al., 2022).

The squamosal’s posterior process is mediolaterally narrow, small, and angled medially. In *Tyrannosaurus rex* (Brochu, 2002) the posterior process is narrower. The elongate ventral process is narrower and more strongly bowed than in *T. rex* (Brochu, 2002); *Tarbosaurus bataar* (Hurum and Sabath, 2003) also has a strongly bowed ventral process. The lateral surface has a ridge defining the quadratojugal articulation. The quadratojugal contact is plate-like as in other tyrannosaurids (Brochu, 2002; Currie, 2003). The ventral process is narrow with a squared-off tip; it is broad and has a pointed tip in *T. rex* (Brochu, 2002).

The squamosal’s medial process is a finger-like projection extending anteriorly, paralleling the anterior process. Posterior to the medial process and oriented dorsomedially lies the contact for the paroccipital process. The paroccipital contact is visible medially and dorsally. Ventral to it, visible in medial view, and facing slightly ventrally is the parietal articulation, which resembles that of *Tarbosaurus bataar* and *Tyrannosaurus rex* (Brochu, 2002) In ventral view, the squamosal’s medial process is sinuous; it is straight in *T. rex*.

**Maxilla.** Fragments that appear to come from the maxilla are preserved; little can be said of their morphology.


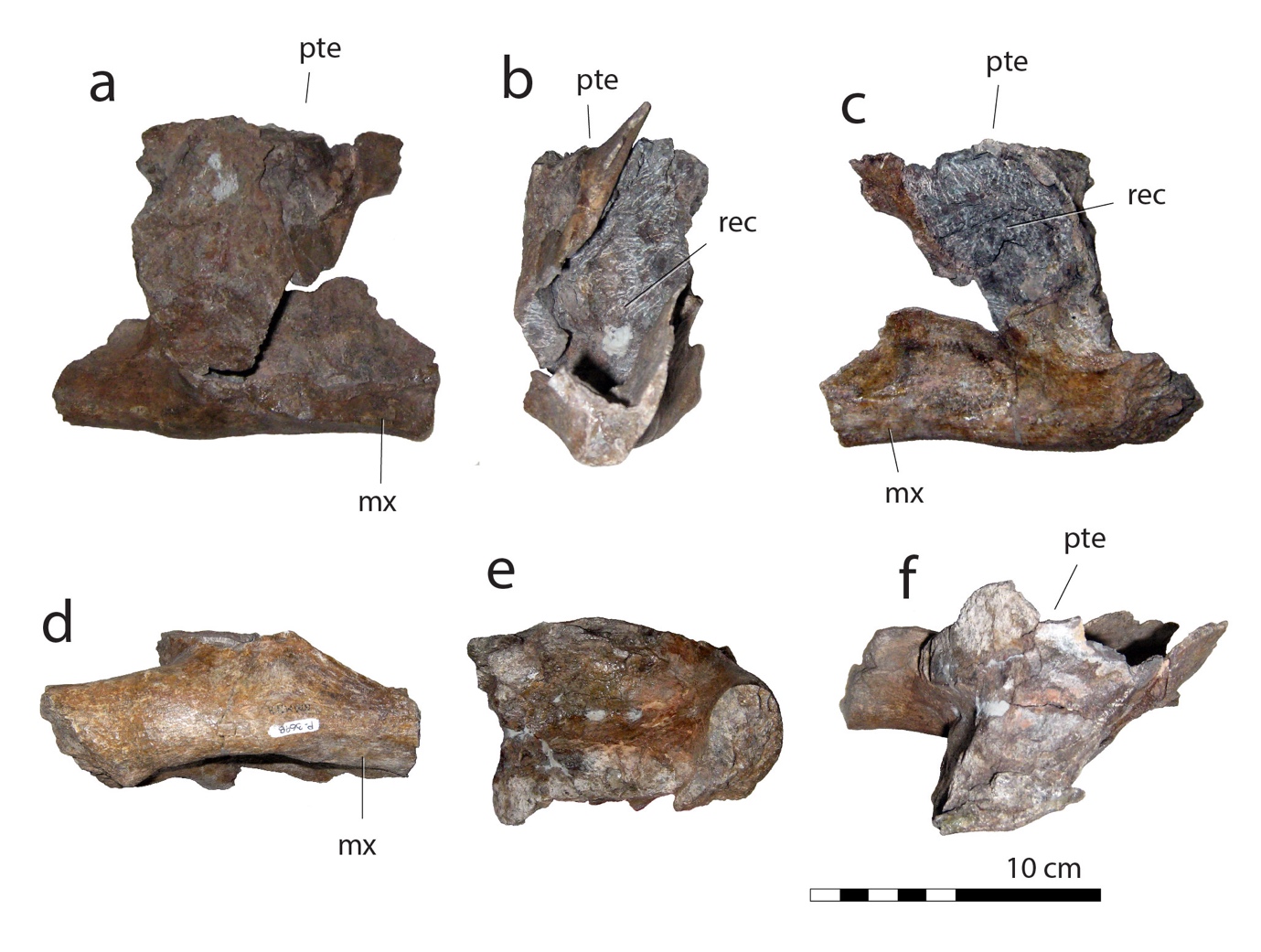


**Fig. S5** Right palatine of NMMNH P-3698 in **a**, lateral, **b** anterior, **c**, medial, **d**, ventral, **e**, posterior, **f**, dorsal views. Abbreviations: mx, maxillary contact; pte, pterygoid contact; rec, palatine recess. Scale = 10 cm.

**Palatine.** The right palatine (Fig. S5) is damaged anteriorly, dorsally and posterolaterally, making it difficult to assess its morphology. It appears to have been relatively broad, similar to *T. bataar* (Hurum and Sabath, 2003) but perhaps not quite to the degree as in *Tyrannosaurus rex*, and unlike that of Albertosaurinae (Currie, 2003). This wide palatine contributes to the formation of a broad muzzle in Tyrannosaurini (Hurum and Sabath, 2003).

As in other tyrannosaurids (Currie, 2003), the palatine is tetraradiate. The pterygoid process projects posteromedially from the medial surface of the palatine. In dorsal view the pterygoid and jugal processes diverge medially and laterally from each other. The anterior process projects anterolaterally and preserves a notch-like contact with the maxilla. Posterolaterally, the jugal process is broken and does not preserve the contacts with the jugal and lacrimal. The vomeropterygoid process projects dorsally to contact the pterygoids and vomers. The medial process projects posteromedially to contact the pterygoid. As in other tyrannosaurids (Brochu, 2002; Currie, 2003; Hurum and Sabath, 2003), the jugal and pterygoid form the anterior border of the suborbital fenestra. The contact with the maxilla along the lateral margin is laterally concave and forms a rugose groove along the suture with the maxilla. In *Tyrannosaurus rex* the contact for the maxilla is flat and sinuous with a rugose surface consisting of elongated grooves and ridges paralleling each other. As in other tyrannosaurids (Currie, 2003), the ventromedial surface is medially concave forming the lateral margin of the internal antorbital fossa. The maxillary contact forms a triangular, groove-like fossa that tapers posteriorly. Dorsal to the contact for the maxilla is a long ridge marking the dorsal surface of the contact, as in other derived tyrannosaurids. In *Tyrannosaurus mcraeensis* the jugal process extends posterodorsal to the maxillary process and in most other tyrannosaurids but contrasting with the condition in *Tarbosaurus* and *Tyrannosaurus rex* where the two processes lie at the same level (Brochu, 2002; Hurum and Sabath, 2003).


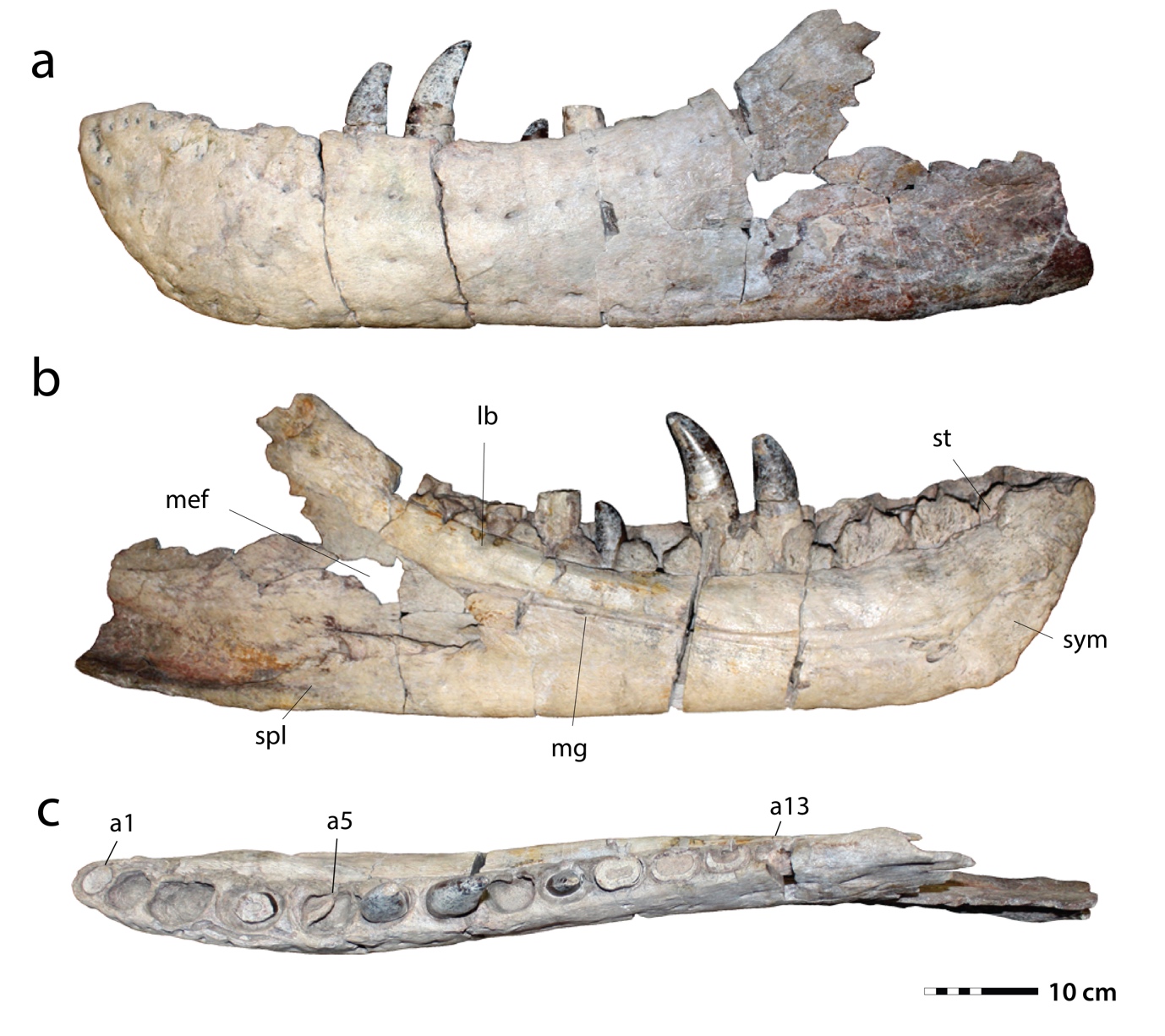


**Fig. S6.** Left dentary of *Tyrannosaurus mcraeensis*, NMMNH P-3698, in **a**, lateral, **b** medial, **c**, dorsal, views. Abbreviations: a1, a5, a13, alveoli 1, 5, and 13, respectively. Abbreviations: lb, lingual bar; mef, Meckelian fossa; mg, Meckelian groove; spl, splenial facet; st, step; sym, symphysis. Scale = 10 cm.

**Dentary.** The dentary (Fig. S6) has 13 alveoli, a derived condition uniquely shared with *Tyrannosaurus rex* (Brochu, 2002) among tyrannosaurids. *Zhuchengtyrannus magnus* has 15 teeth, accounting for damage at the tip of the jaw (Hone et al., 2011), while *Tarbosaurus bataar* has 14-15 teeth (Hurum and Sabath, 2003); *Daspletosaurus horneri* has 17 (Carr et al., 2017). The first alveolus is reduced relative to the second, as in other Tyrannosaurinae.

In lateral view, the dentary is robust. The occlusal margin rises up towards the tip of the jaw and steeply at the back creating a strongly concave dorsal margin, as in other Tyrannosaurini (Brochu, 2002; Hurum and Sabath, 2003; Hone et al., 2011; Loewen et al., 2013). Unusually, the ventral margin of the dentary is convex; it is concave beneath the back of the tooth row in all other tyrannosaurids (Brochu, 2002; Currie, 2003; Loewen et al., 2013). This condition is unique to *Tyrannosaurus mcraeensis* but approached in *Tarbosaurus bataar* (Brochu, 2002; Hurum and Sabath, 2003) and *Zhuchengtyrannus magnus* (Hone et al., 2011). The angular process and splenial facet are directed posteriorly, as in *Tarbosaurus bataar* and *Zhuchengtyrannus magnus* (Hone et al., 2011) but unlike *Tyrannosaurus rex* and all other tyrannosaurids, in which the angular process projects posteroventrally (Brochu, 2002; Currie, 2003; Loewen et al., 2013; Dalman and Lucas, 2017).

In dorsal view the anterior end of the dentary bows outward anteriorly so that the dentaries would form a broad “U” shape. A similar bowed dentary occurs in *Tyrannosaurus rex* (Brochu, 2002; Loewen et al., 2013) and *Tarbosaurus* compared to the straighter dentary in *Lythronax argestes* (Loewen et al., 2013); the dentary is straight in *Nanuqsaurus hoglundi* (Fiorillo and Tykoski, 2014) and more basal taxa (Brusatte et al., 2012; Nesbitt et al., 2019). The dentary and toothrow then curve outwards toward the back of the jaw. Together these characters suggest a posteriorly expanded skull, as in *Tarbosaurus rex, Tarbosaurus bataar* (Brochu, 2002; Hurum and Sabath, 2003), and *L. argestes* (Loewen et al., 2013), although *Lythronax* achieves the expansion through the angle of its symphysis.

Medially, the interdental plates are large and rectangular to triangular in shape, as in other tyrannosaurines (Brochu, 2002; Hone et al., 2011); interdental plates are smaller in basal tyrannosaurids (Currie, 2003; Brusatte et al., 2012). The lingual bar covers the first two interdental plates in medial view. The lingual bar is deep anteriorly but strongly narrows posteriorly, to half of its anterior depth, as in *Tyrannosaurus rex* (Brochu, 2002; Dalman and Lucas, 2017); this condition is approached by *Tarbosaurus bataar* (Hurum and Sabath, 2003) and *Zhuchengtyrannus magnus* (Hone et al., 2011) although it is not developed to the same degree.

The symphysis is rugose and covered with bumps and grooves where the dentaries contacted, as in other tyrannosaurines. The symphysis extends back to the third alveolus, as in *Tarbosaurus bataar* (Hurum and Sabath, 2003). In *Tyrannosaurus rex* it extends back to the third (Brochu, 2002) or fourth (Osborn, 1905) alveolus, while in *Zhuchengtyrannus magnus* (Hone et al., 2011) it ends under the fourth alveolus. The dentary symphysis ends just above the dentary’s ventral margin, as in *Tarbosaurus bataar* (Hurum and Sabath, 2003) and *Zhuchengtyrannus magnus* (Hone et al., 2011); in *Tyrannosaurs rex* the symphysis sometimes projects ventrally to end below the dentary ventral margin, but in other specimens it lies above, as in NMMNH P-3698.

The large splenial facet is convex anteriorly and concave posteriorly. The facet’s dorsal surface slopes gently down posteriorly and is uninterrupted; in *Tyrannosaurus rex*, *Tarbosaurus bataar*, and *Zhuchengtyrannus magnus* the splenial facet is short and interrupted by a transition at the level of tooth position 13.


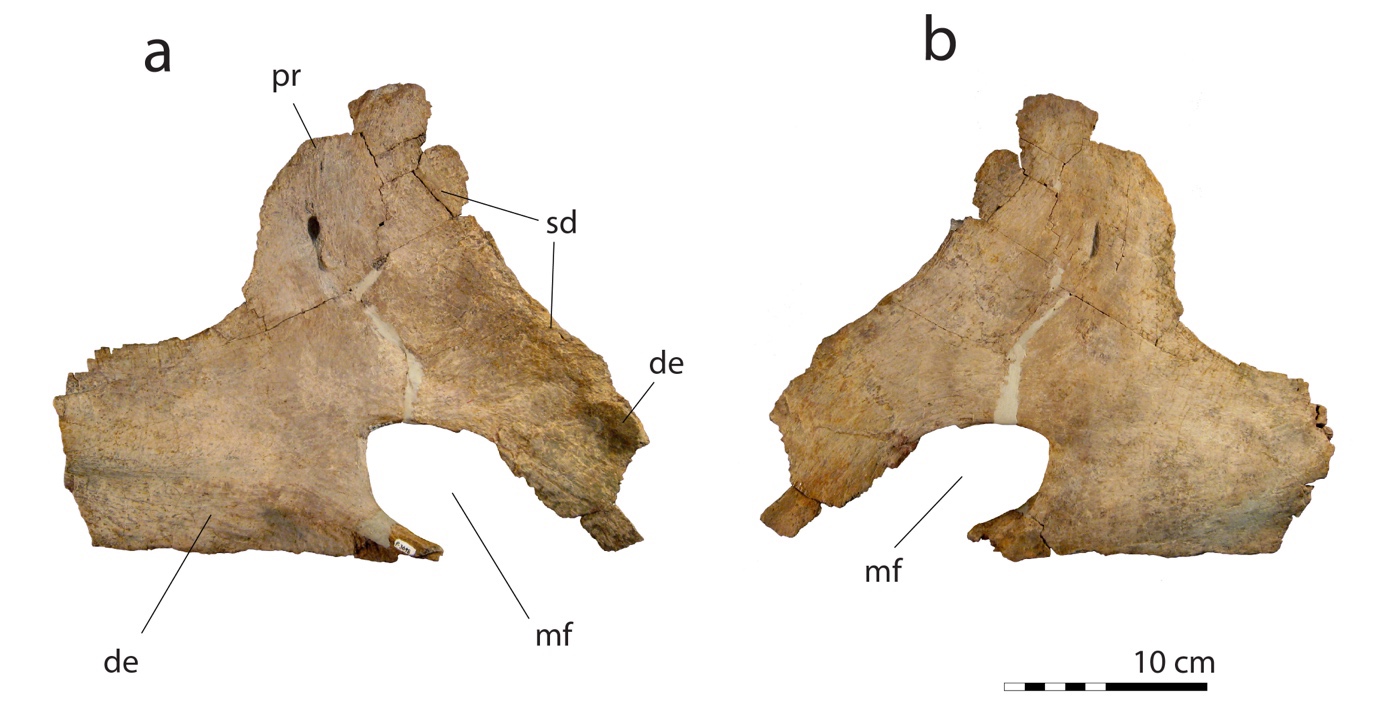


**Fig. S7.** Right splenial of *Tyrannosaurus mcraeensis*, NMMNH P-3698, in **a**, medial, **b** lateral views.

Abbreviations: de, dentary contact; mf, mylohyoid foramen; pr, prearticular; sd, supradentary contact.

**Splenial.** The splenial (Fig. S7) is triangular, as in other tyrannosaurids (Brochu, 2002; Currie, 2003; Hurum and Sabath, 2003); it contacted the dentary ventrally and anterodorsally, and the supradentary and prearticular dorsally. The anterior and posterior ends are broken off, as are the processes bounding the mylohyoid foramen.

The tall dorsal flange resembles *Tarbosaurus bataar* (Hurum and Sabath, 2003) and *Tyrannosaurus rex* (Currie, 2003);  *Gorgosaurus libratus* (Currie, 2003) has a lower dorsal flange. In horizontal view the apex is inclined dorsally and the apex of the splenial lies above the anterior mylohyoid fenestra; in *T. rex* (BHI 3033, FMNH PR 2081, LACM 157776, MOR 008, MOR 1125, TMP 1981.6.1) the apex lies slightly more posteriorly. It is triangular in shape, similar to *Tarbosaurus*; in *T. rex* the dorsal flange is quadrangular in most specimens (MOR 1125, TMP 1981.6.1), but triangular in some others (BHI 3033).

The ventral margin of the splenial is straight, versus strongly bent in *Tarbosaurus bataar* (Hurum and Sabath, 2003) and most individuals of *Tyrannosaurus rex* (BHI 3033, MOR 555; MOR 1125), although the splenial is more weakly bent in some specimens of *T*. *rex* (FMNH PR 2081, TMP 1981.6.1). The posteroventral process of the splenial extends caudally, versus being posteriorly directed in *T*. *rex* (BHI 3033, FMNH PR 2081, LACM 157776, MOR 008, MOR 1125, TMP 1981.6.1).

The posterior process of the splenial is deep, as in *Tarbosaurus bataar*; it is more slender in *T. rex* (MOR 555; MOR 1125, TMP 1981.6.1), although in some specimens (FMNH PR 2081) it is slightly deeper, approaching the condition in NMMNH P-3698. The anteroventral process forming the ventral margin of the anterior mylohyoid fenestra is broken but appears to have a different shape than in *T. rex*, with a concave ventral surface that would have wrapped over the dentary; in *T. rex* there is a simple, broad, laterally facing facet that abutted the medial surface of the dentary.

The anterior process of the splenial is deep. Along the dorsal margin of the process a groove extends for the supradentary contact, as in other tyrannosaurids (Brochu, 2002; Currie, 2003; Hurum and Sabath, 2003).


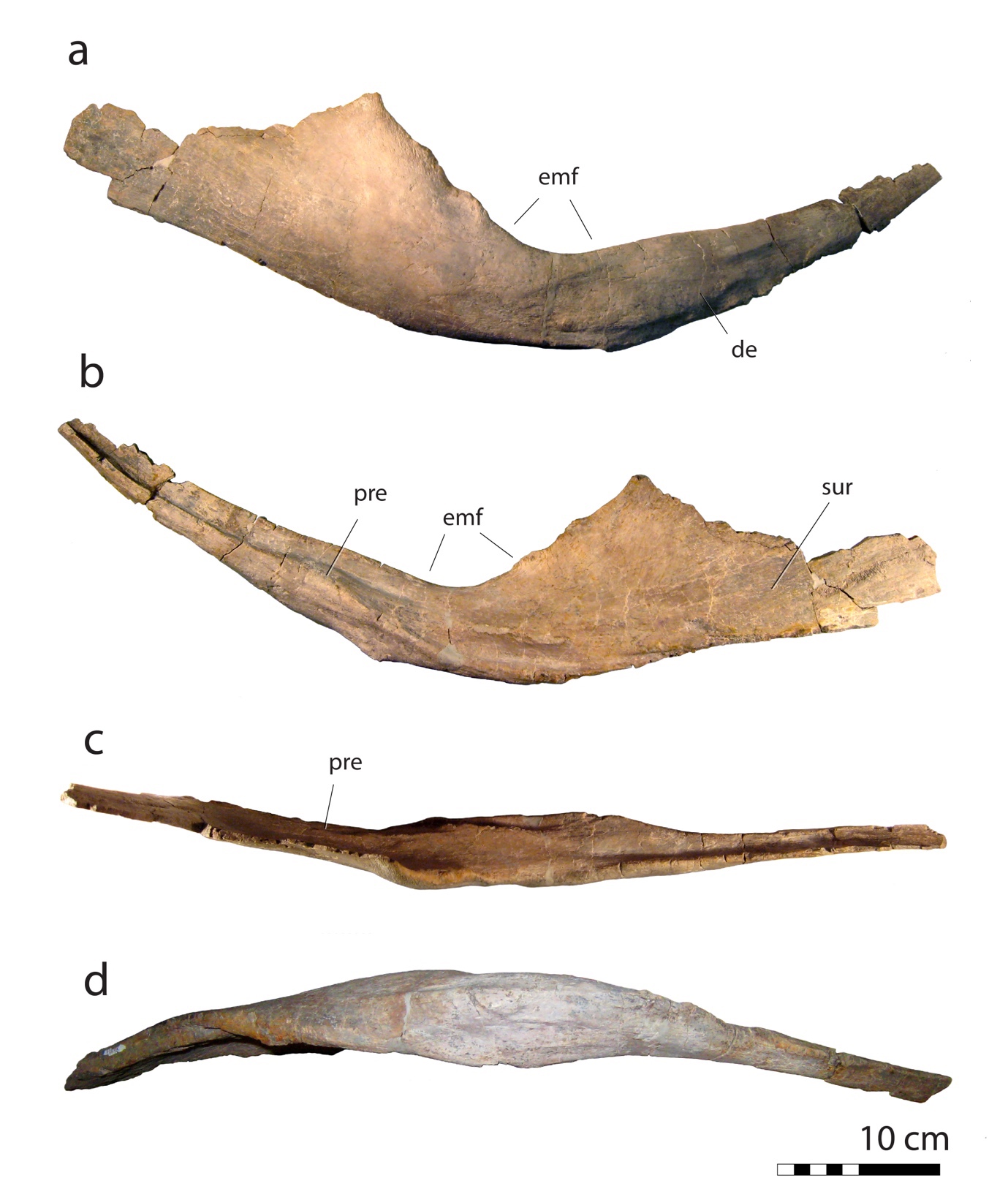


**Fig. S8.** Right angular of *Tyrannosaurus mcraeensis*, NMMNH P-3698, in **a**, lateral, **b** medial, c, dorsal, d, ventral views. Abbreviations: de, dentary contact; emf, external mandibular fenestra; pre, prearticular facet, sur, surangular facet.

**Angular.** The angular (Fig. S8) consists of an anterior prong and posterior plate. The anterior process is longer and more slender in *Tyrannosaurus mcraeensis* than in *T. rex* (BHI 3033). The anterior process of the angular is narrower than in *T. rex*; here *T. rex* has a broad anteroventral flange (BHI 3033; LACM 23844; MOR 1125, RSM P2523.8) absent from NMMNH P-3698.

The anterior flange bears several ridges medially. The most pronounced is the medial ridge, extending along the anterior process. A similar ridge occurs in *Tyrannosaurus rex* but does not contact the plate’s anteroventral margin. Dorsal to the posterior end of the ridge is a wide fossa separating the ridge from the medial ridge. A similar fossa occurs in *T. rex* but is short and anteriorly enclosed by the union of two ridges; in *Tyrannosaurus mcraeensis* the fossa invades the dorsal surface of the medial ridge of the anterior process. The angular of *T. mcraeensis* bears an ovoid fossa at the mid-length of the medial ridge of the anterior process. Anterior to this fossa the ridge extends anteriorly and terminates at the anteroventral margin of the process. In contrast, in *T. rex* an elongate fossa lies directly below the ridge, extending anteroposteriorly. In *T. mcraeensis*, dorsal to the medial ridge there is a characteristic elongate fossa, running the length of the ridge. Ventral to the medial ridge is another fossa, forming a low, inverted triangle. In the angular of *T. rex,* the ventral portion of the anterior process is strongly expanded, forming a large flange. A similar ventral flange occurs in *Daspletosaurus torosus* and in *Tarbosaurus bataar*. However, in these taxa the flange is smaller than in *T. rex*, and more closely resembles that of *T. mcraeensis*.

The posterior plate’s ventral margin is convex anteriorly, then slightly concave posteriorly, as in *T. rex* (Brochu, 2002). The lateral surface of the angular is convex. The surface is smooth along the posterior plate, but the anterior process bears several ridges and fossae to articulate with the dentary. A shallow fossa representing the contact with the dentary extends onto the lateral surface of the posterior plate. In *T. rex* a similar, deeper fossa lies here. The medial surface of the angular is concave, as in other tyrannosaurids (Brochu, 2002; Currie, 2003). The concavity forms a fossa, which is deepest anteriorly. Below this fossa is a short ridge; another ridge lies posterodorsally. A similar fossa and ventral ridge occur in *T. rex*, but it lacks the dorsal ridge seen in *T. mcraeensis*. Furthermore, in *T. mcraeensis* another ridge lies along the ventral margin of the medial surface of the plate; this ridge is absent in *T. rex*.


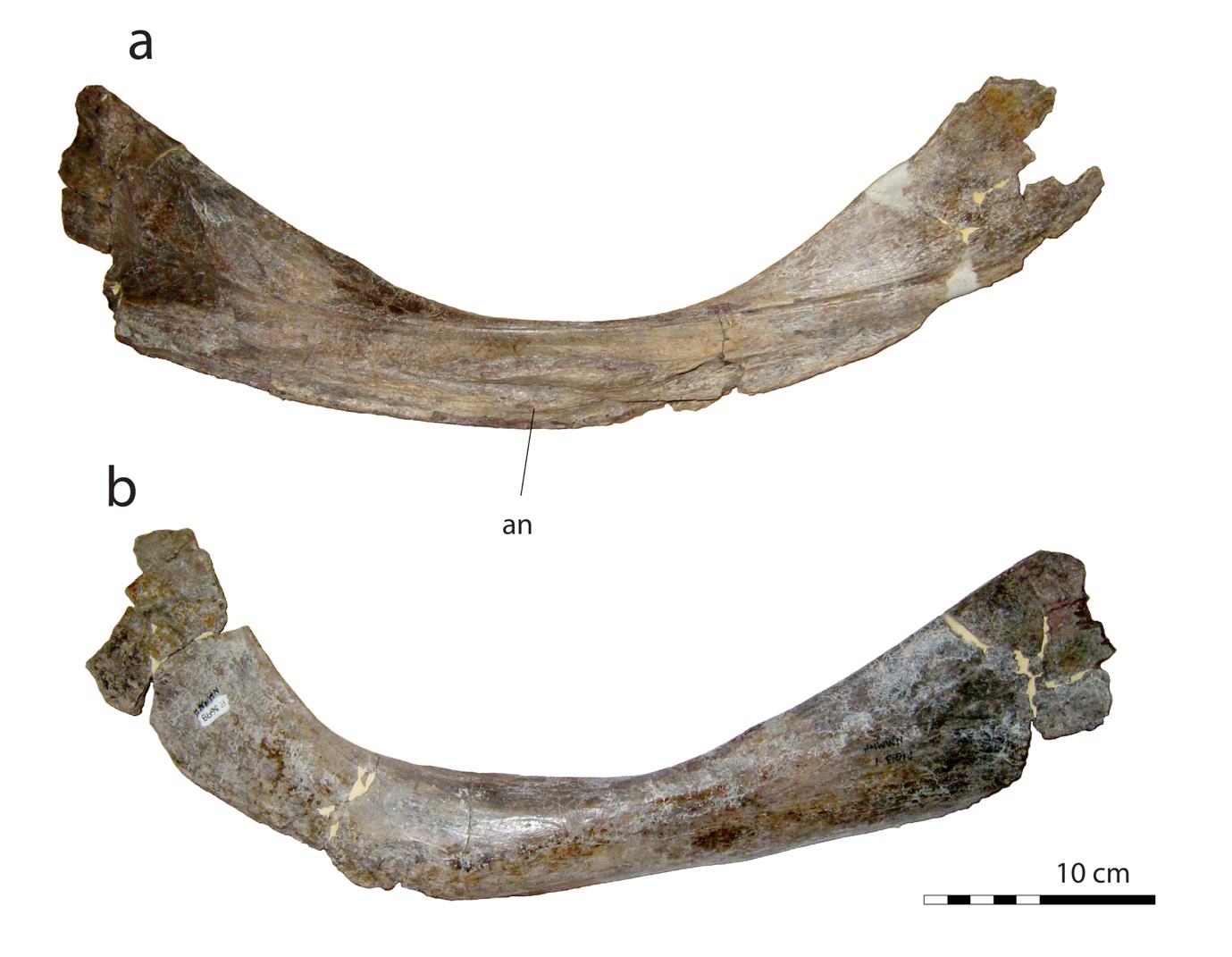


**Fig. S9.** Right prearticular of *Tyrannosaurus mcraeensis*, NMMNH P-3698, in **a**, lateral, **b** medial views.

Abbreviations: an, angular contact.

**Prearticular.** The right prearticular (Fig. S9), resembles other tyrannosaurids (Brochu, 2002; Hurum and Sabath, 2003; Brusatte et al., 2012) in being a long, curved strap-like bone. It would have contacted the angular laterally, and the surangular and articular posteriorly, and the splenial, coronoid, and dentary. It is gently curved, versus strongly bent and L-shaped in *Tyrannosaurus rex* (AMNH 5029, AMNH 5117, BHI 3033, FMNH PR 2081, LACM 23844; MOR 008, MOR 1125) and *Tarbosaurus*.

In *Tyrannosaurus mcraeensis*, the anteroventral margin of the prearticular bears a concave notch. A similar, more pronounced notch occurs in *Alioramus* *altai* (Brusatte et al., 2012), *Tarbosaurus bataar* (Hurum and Sabath, 2003), and *T. rex* (Brochu, 2002). Anterior to this notch the ventral margin is convex, versus straight in *T. rex*. The anterior process’s dorsal and ventral margins are parallel, as in *Tarbosaurus* (Hurum and Sabath, 2003) and *T. rex* (Brochu, 2002) but the prearticular anterior process is wider than in *T. rex*. The lateral surface of the prearticular contacts the medial surface of the coronoid, and the splenial overlapped the medial surface of the prearticular, as in other tyrannosaurids (Brochu, 2002; Currie, 2003; Hurum and Sabath, 2003; Brusatte et al., 2012).

The prearticular contacts the angular laterally along a suture consisting of two large, deep grooves, lying dorsally and ventrally. The dorsal groove extends from the base of the anterior process to the base of the posterior process; the ventral groove emerges from the base of the posterior process and terminates at the middle of the prearticular. The dorsal groove is split by a low, anteroposteriorly oriented ridge. The anterior end of this ridge lies at the posterior margin of the ventral notch. The arrangement of the ridges on the articular surface differs from *Tarbosaurus* and *Tyrannosaurus rex*. In *T. rex* at least seven ridges divide the articular surface, reinforcing the angular contact. The prearticular of *T. mcraeensis* is more weakly twisted than in *Tarbosaurus* (Hurum and Sabath, 2003) or *T. rex* (Brochu, 2002). In *T. rex* the twisting is so extensive that the lateral ridges, especially in the anterior region, extend beyond the anteroventral margin of the element. The ventral contact for the angular is relatively poorly developed in *T. mcraeensis*, while that of *T*. *rex* has a large ventral articulation (AMNH 5117; BHI 3033; FMNH PR 2081; MOR 1125).


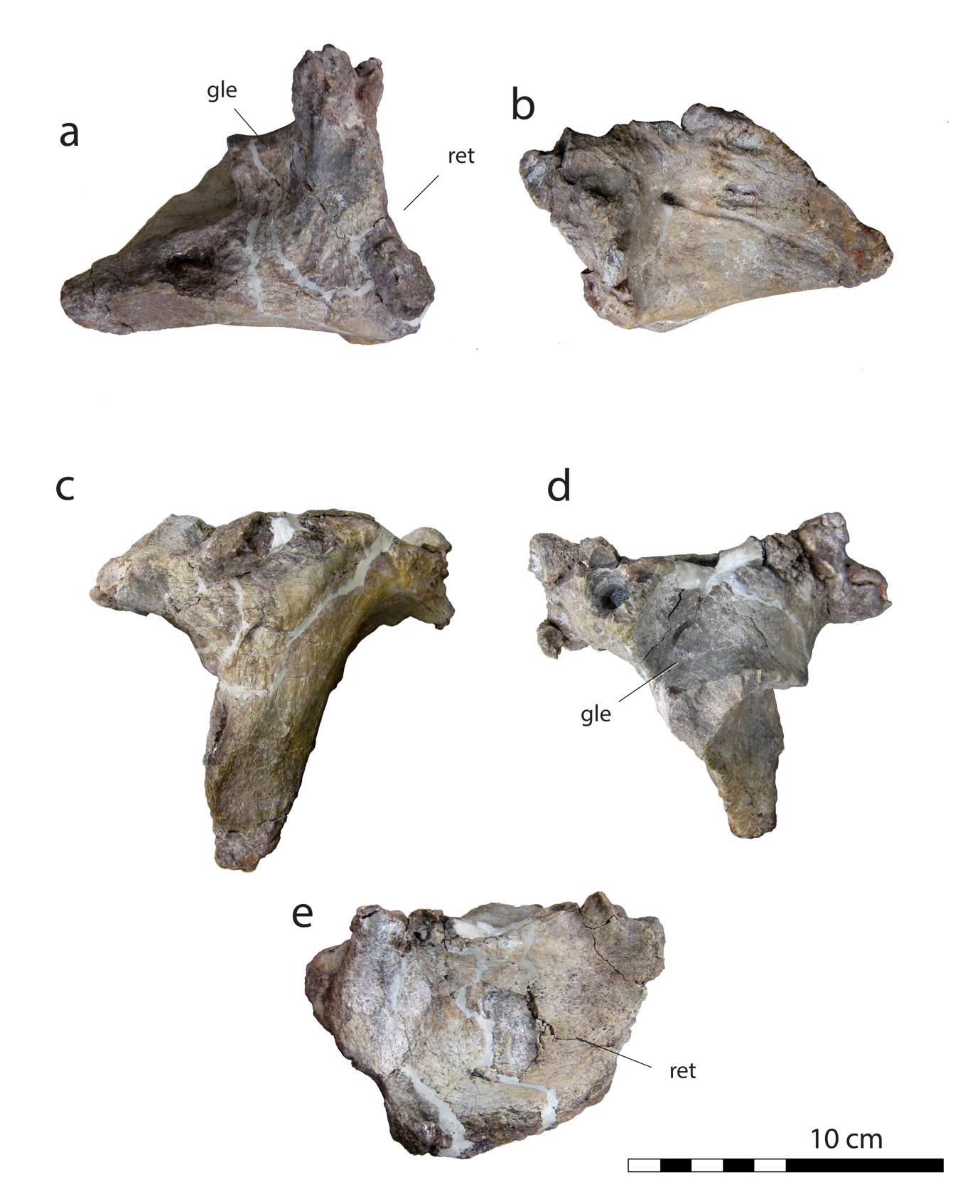


**Fig. S10.** Left articular of *Tyrannosaurus mcraeensis*, NMMNH P-3698, in **a**, lateral, **b** medial, **c** ventral, **d**, dorsal, and **e**, posterior views. Abbreviations: cot, cotyle; gle, glenoid fossa; ret, retroarticular process.

**Articular.** The articular of *Tyrannosaurus mcraeensis* is a subtriangular element (Fig. S10). The retroarticular process is shallowly concave and lacks the semi-circular outline seen in *T. rex* (Fig. S10); instead, the medial margin has an angular outline. It is convex along the dorsomedial margin and concave ventromedially.

In ventral view the articular has a T-shape; in *Tyrannosaurus rex* it is triangular. The “T”-shaped articular of *T. mcraeensis* results from a mediolaterally expanded retroarticular process and a long anterior process. In posterior view the articular process’ dorsal margin is slightly convex. As in other tyrannosaurids (Brochu, 2002; Hurum and Sabath, 2003), the dorsal surface of the articular is complex. The glenoid fossa/quadrate articulation is a saddle-shaped depression divided by a low ridge. The lateral depression is larger and extends lateromedially and anteroposteriorly, the smaller medial depression is semicircular, lies in the posteromedial corner of the bone and is slightly elevated. Anterior to the depression the bone surface is elevated, and in dorsal view forms a triangle, flaring medially then gradually tapering laterally. The surangular-articular contact consists of a deep fossa on the lateral surface of the anterior process of the articular and a smaller circular fossa adjacent to the posterior margin of the articular. As in other tyrannosaurids, but especially *Tarbosaurus bataar* (Hurum and Sabath, 2003) and *T. rex* (Brochu, 2002), the posterior walls of both fossae are nearly vertical. A lateromedial ridge and a groove on the dorsal surface of the articular separate the glenoid fossa from the retroarticular process. In contrast, in *T. rex* only a groove is present here; the ridge is absent (Brochu, 2002). The articular of *T. mcraeensis* has a single large, circular foramen for the chorda tympani posterior to the glenoid fossa. In *T. rex* there are two foramina, one on each side of the articular (Brochu, 2002). Another foramen lies on the medial surface of the anterior process. Directly anterior to this foramen is elongate, narrow, channel-like sulcus extending along the anterior process. The foramen occurs in most other tyrannosaurids and may be the exit for the chorda tympani channel (Brochu, 2002). Dorsal to the sulcus is a strong rugosity consisting of irregular ridges and grooves, extending anterodorsally. Near the dorsal ridge of the anterior process the bone is excavated by a pneumatic fossa. A similar but more pronounced pneumatic fossa occurs on the surangular contact surface. In medial view the retroarticular process strongly projects posteriorly beyond the posterior margin of the articular. The ventral surface of the articular is rugose, with anteroposteriorly oriented lineations invading the posterior end of the anterior process. The most prominent rugosity lies at the anterior end of the anterior process and forms a fossa that extends lateromedially for nearly half the length of the process. The fossa is deepest along the lateral margin of the anterior process, and a sharp ridge marks its lateral margin. Posterior to this ridge the bone is convex but along the medial surface extends a long, sharp ridge. In *T. rex* the ventral surface of the anterior process of the articular is convex and lacks the rugosity seen in *T. mcraeensis*.


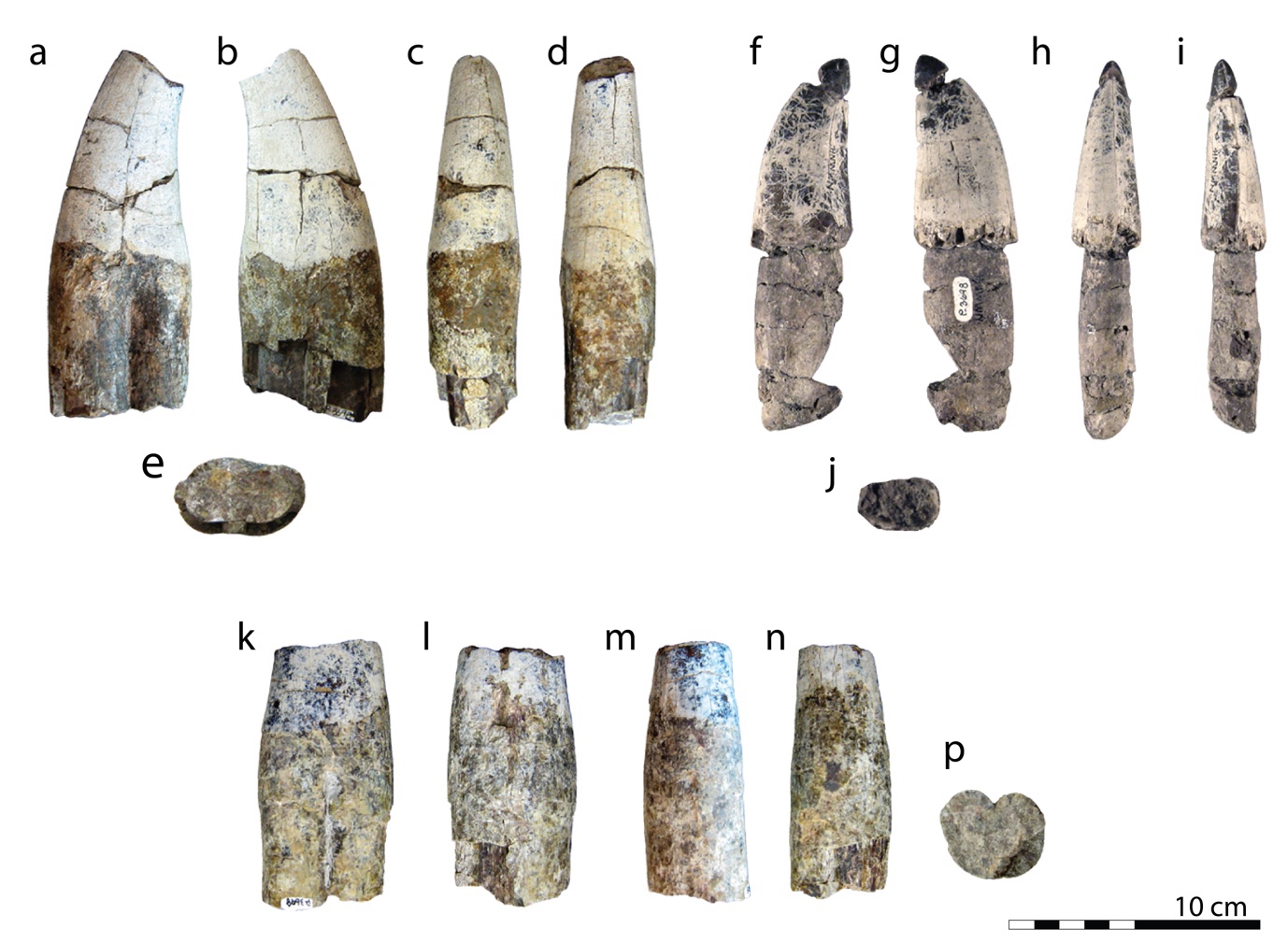


**Fig. S11.** Isolated teeth from the right dentary / left maxilla, NMMNH P-3698, holotype of *Tyrannosaurus mcraeensis*. In a, f, k, lingual view; b, g, l, labial view; c, h, m, mesial view; d, i, n, distal view; e, j, p, basal view.

**Teeth.** Nine teeth are preserved partially or wholly in the left dentary; alveoli 6, 7, and 9 preserve complete teeth; 1, 4, 5, 11 and 12 have broken teeth. In addition, six isolated teeth are preserved (Fig. S11). Based on the curvature of the mesial carina, which twists towards the lingual surface of the crown, some come from the right dentary and / or left maxilla.

Teeth resemble those of *Tyrannosaurus rex* (Smith, 2005) in having massive crowns. The labiolingual width of some teeth approaches their mesiodistal length, as in *T. rex*; these presumably represent anterior teeth. Others, presumably representing posterior teeth, are more laterally compressed. Mesial and distal carinae are serrated. As is typical of Tyrannosauridae, the carinae are twisted. The mesial carina twists such that it lies anteriorly towards the apex of the tooth crown, then twists towards the lingual surface towards the base of the crown; the distal carina is shifted laterally. This twisting is well-developed in large teeth from the front of the jaws but less so from smaller teeth from the back of the jaws.

They resemble maxillary teeth, being large, robust, and labiolingually expanded. Tooth crowns have massive apices, similar to *Tarbosaurus* (Hurum and Sabath, 2003); *Tyrannosaurus rex* has more elongate, pointed tooth apices (Brochu, 2002; Smith, 2005), the so-called “railroad spike” morphology. The sixth tooth has a worn apex; *T. rex* tooth wear is often heavy due to biting bone (Erickson and Olson, 1996; Longrich et al., 2010; Gignac and Erickson, 2017).

**3. Extended Diagnosis**

*Diagnosis*. *Tyrannosaurus mcraeensis* is distinguished from *Tyrannosaurus rex* by the following characters:

1. Postorbital cornual process low, blunt, and located over the posterior edge of the orbit, versus a tall and prominent cornual process, often with a distinct and narrow apex, located over the orbit in *T. rex.*
2. Anteriorly projecting prefrontal/frontal articulation, versus an anteroventrally projecting prefrontal/frontal articulation in *T*. *rex*.
3. Squamosal quadratojugal process strongly downturned, versus more anteriorly oriented in *T*. *rex*.
4. Squamosal with a prominent ridge bounding the anterior margin of the ventral fossa, versus, versus a highly reduced or absent ridge in *T*. *rex*.
5. Squamosal medial margin strongly concave; In NMMNH P-3698, the medial margin of the squamosal is strongly concave. It is weakly concave in *Tyrannosaurus rex* (CM 9380; LACM 150167, RSM P2523.8).
6. posterior of dentary shallow and with a convex posteroventral margin, versus deep and downturned in *T*. *rex*.
7. Splenial apex directed dorsally, versus posterodorsally in *T*. *rex.*
8. Splenial anteroventral process with a distinct shelf where it contacts the dentary, versus an abutting contact in *T*. *rex*.
9. Posterior (angular) process of splenial directed posteriorly, versus sigmoidal curvature in *T*. *rex*.
10. Prearticular weakly bowed, versus strongly curved upwards where it contacts the splenial in *T*. *rex*.
11. Angular-splenial contact weakly developed, versus a prominent angular contact on the prearticular, and an angular with a large anteroventral flange for the prearticular in *T*. *rex*.
12. Articular with a deep, quadrangular shape in posterior view, versus wide and crescentic in *T*. *rex*.
13. Articular T-shaped in ventral view, versus triangular in *T*. *rex*.

The large number of specimens known for *Tyrannosaurus* make possible extensive comparisons. This means that even where morphological differences may appear minor, they can nevertheless diagnose a distinct taxon if these small differences are consistently absent in *Tyrannosaurus rex*, as is the case with the characters above.

Comparisons with *Tyrannosaurus rex* (Figs. S12-S20; see also character discussion below) show that the features cited above cannot be explained in terms of individual variation in *T*. *rex*. Each of the diagnostic characters seen in NMMNH P-3698 has been observed to be absent in *T*. *rex* in multiple specimens, and in all examples studied. Furthermore, differences can be seen in every one of the bones of the skull. This is inconsistent with referral of NMMNH P-3698 to *T*. *rex* and supports the identification of a distinct genus and species.

*Specimens*. We considered the following specimens in creating the diagnosis: AMNH 5029; AMNH 5117, BHI 3033, FMNH PR 2081, MOR 555, MOR 1125, RSM P2523.8, TMP 1981.6.1, UMNH 11000.


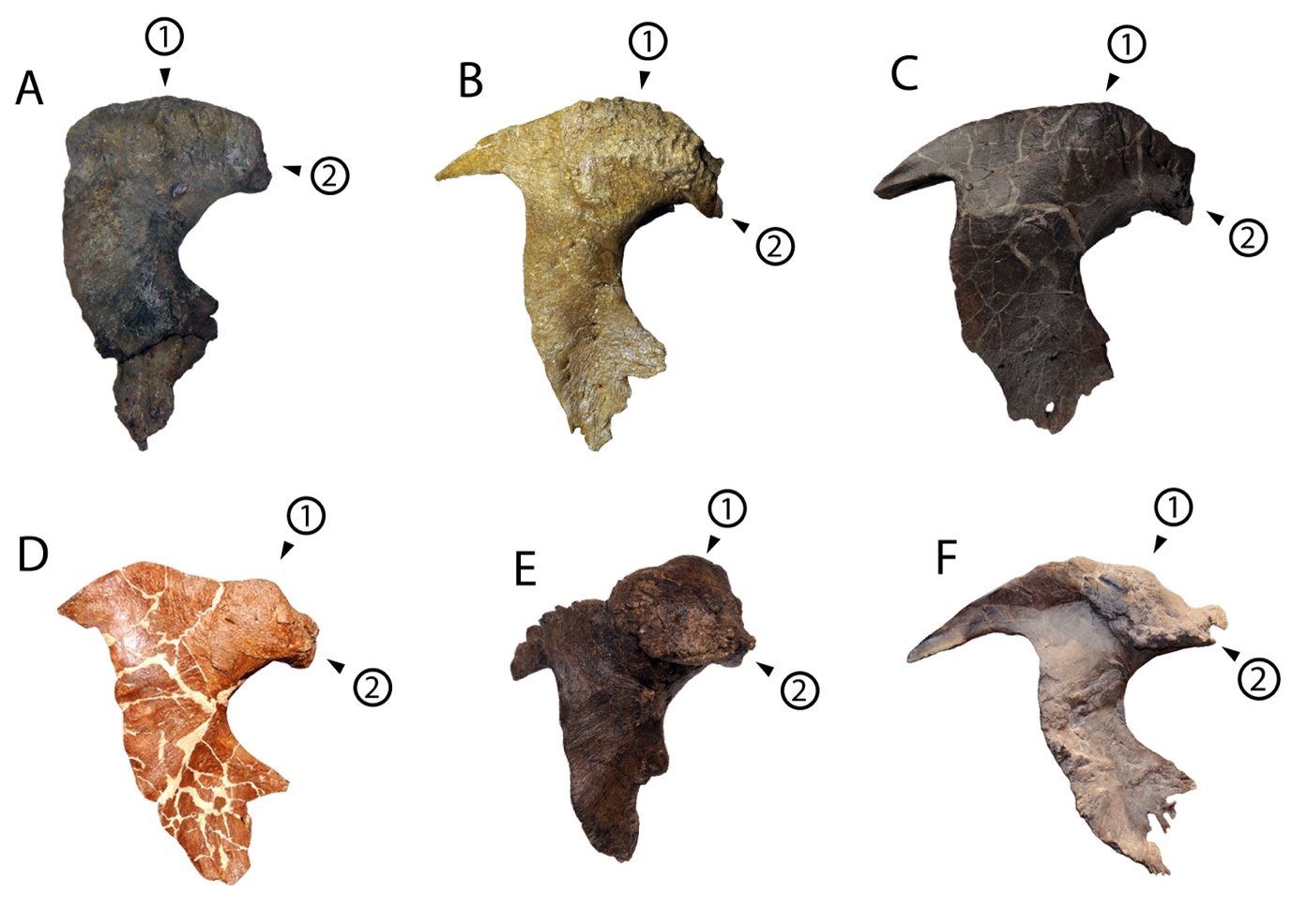


**Figure S12.** Postorbitals of *Tyrannosaurus mcraeensis* and *Tyrannosaurus* *rex* in lateral view. **a**, *Tyrannosaurus mcraeensis* NMMNH P-3698. **b**-**f**, *Tyrannosaurus rex*: **b**, MOR 1125; **c**, LACM 150167; **d**, MOR 980; **e**, FMNH PR2081; **f**, RSM P2523.8.

(1) *Postorbital cornual process low, blunt, and located over the posterior edge of the orbit*. The cornual process in NMMNH P-3698has a very low, broadly rounded dorsal margin with an apex just behind the orbit. That of *Tyrannosaurus is* highly variable, but it always has a taller profile, with a more anteriorly positioned apex of the cornual process above the back or center of the orbit (AMNH 5177, BHI 3033, FMNH PR 2081, MOR 555, MOR 1125, RSM P2523.8, TMP 1981.6.1, UMNH 11000).

(2) *Anteriorly projecting prefrontal/frontal articulation*. The anterior or prefrontal process of the postorbital projects anteriorly in NMMNH P-3698. That of *Tyrannosaurus* is distinctly hooked downwards in lateral view (AMNH 5177, BHI 3033, FMNH PR 2081, MOR 008, MOR 555, LACM 150167, MOR 1125, RSM P2523.8, TMP 1981.6.1, UMNH 11000).


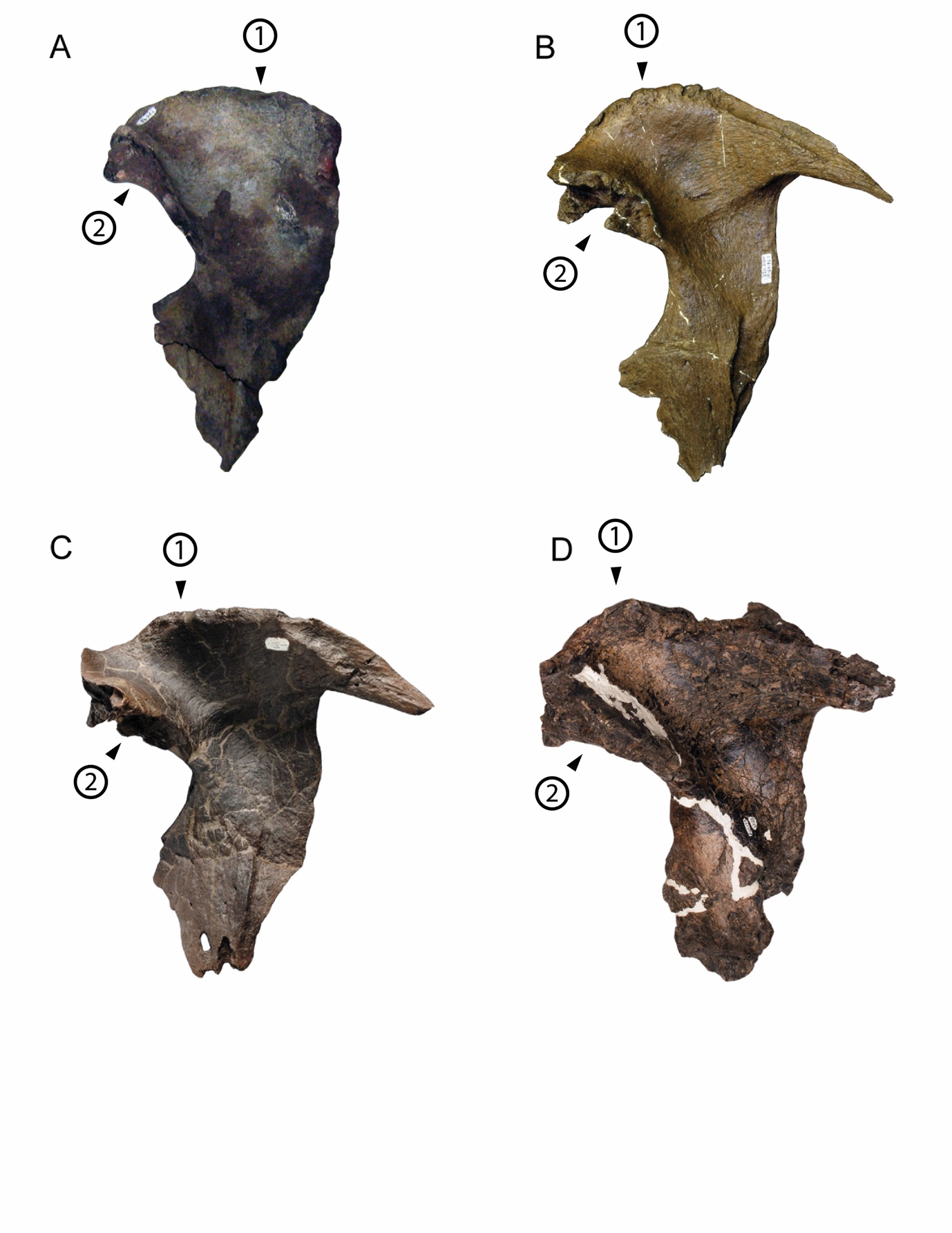
 **Fig. S13.** Postorbitals of *Tyrannosaurus mcraeensis* and *T*. *rex* in medial view. **a**, *Tyrannosaurus mcraeensis*. **b**-**d**, *Tyrannosaurus rex*: **a**, NMMNH P-3698; **b**, MOR 1125; **c**, LACM 150167; **d**, RSM P2523.8. Note that in medial view the cornual process (1) is lower and its apex is more posteriorly located in NMMNH P-3698; that of *Tyrannosaurus rex* is taller and more anteriorly located. The frontal/prefrontal contact (2) is located more ventrally in *Tyrannosaurus rex*.


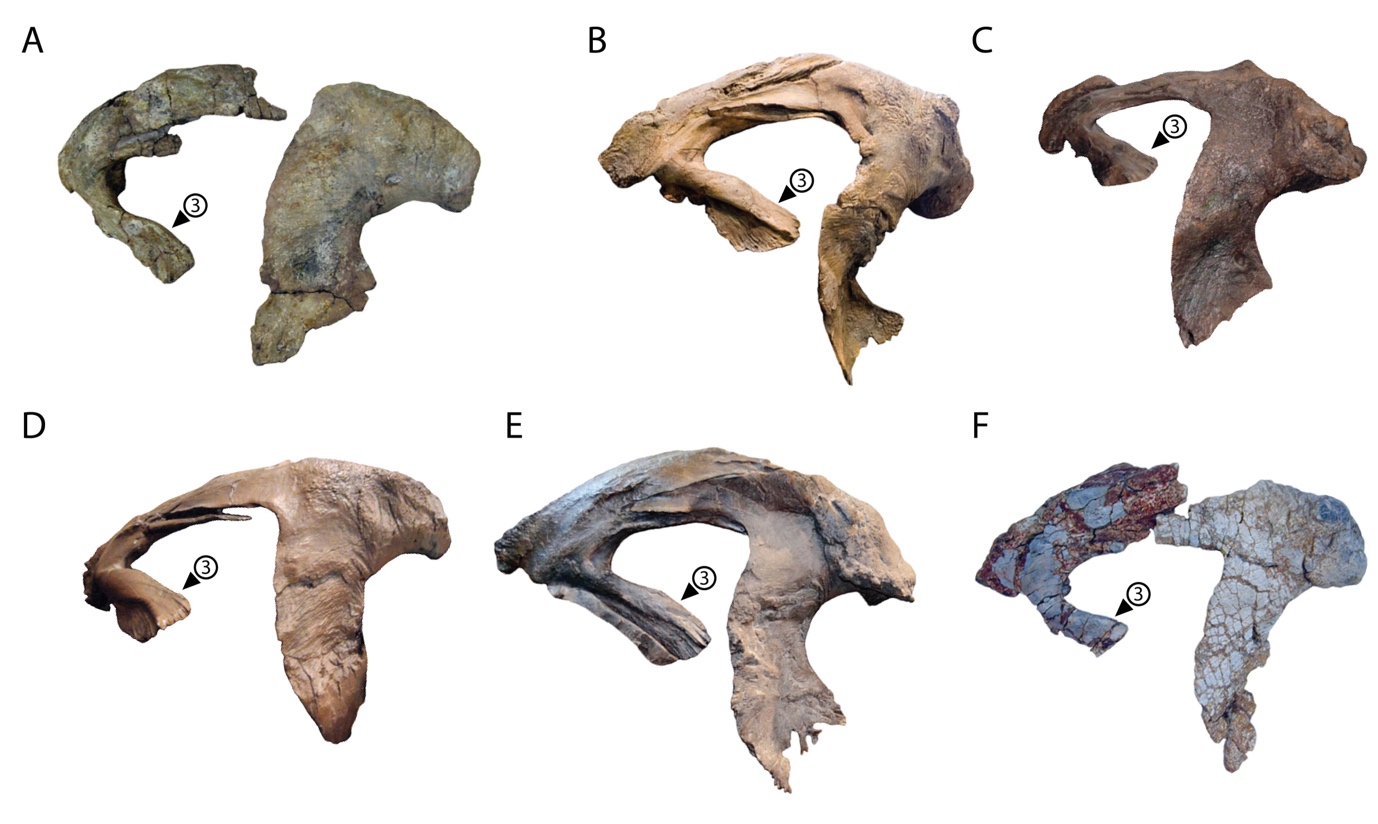


**Fig. S14.** Squamosal-postorbital complex of *Tyrannosaurus mcraeensis* **a**, compared to *Tyrannosaurus rex* **b-f**. **a**, NMMNH P-3698; **b**, CM 9380; **c**, BHI 3033; **d**, MOR 555; **e**, FMNH PR2081; **f**, UMNH VP 11000).

(3) *Squamosal quadratojugal process downturned*. In *Tyrannosaurus mcraeensis*, the quadratojugal process extends anteroventrally and its tip is downturned to that it forms almost a right angle to the main body of the squamosal (Fig. S14). In *T*. *rex*, the quadratojugal process extends more anteriorly and is straighter, forming an acute angle with the squamosal body (AMNH 5027, BHI 3033, CM 9380, FMNH PR 2081, MOR 555, RSM P2523.8, UMNH VP 11000).

**
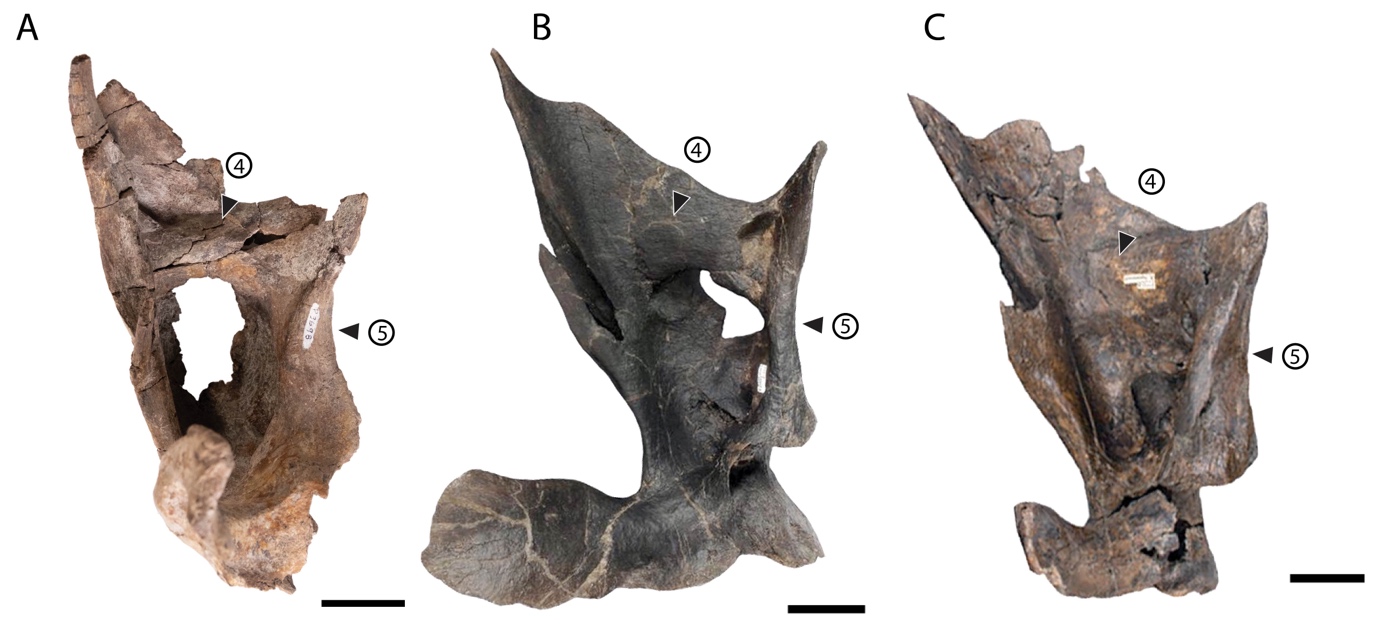
Fig. S15.** Squamosal of **a**, *Tyrannosaurus mcraeensis* and **b,c** *Tyrannosaurus rex* compared. Right squamosals of **a**, NMMNH P-3698; **b**, LACM 150167, **c**, RSM P2523.8. Scale = 5 cm.

(4) *Squamosal ventral fossa bounded anteriorly by a large ridge.* The squamosal in tyrannosaurids is excavated ventrally by a large, deep fossa. In NMMNH P-3698, this fossa is bounded anteriorly by a wide, prominent ridge running transversely across the anterior margin of the ventral fossa (Fig. S15), as in other tyrannosaurids, e.g., *Gorgosaurus libratus* TMP 94.143.1 (Currie, 2003). In *Tyrannosaurus rex*, this fossa is bounded posteriorly, but not anteriorly, such that the ridge is lost and the bone ahead of the fossa extends smoothly (BHI 3033, CM 9380, LACM 150167, MOR 555, MOR 1125, RSM P2523.8).

(5) *Squamosal medial margin strongly concave*. In NMMNH P-3698, the medial margin of the squamosal is strongly concave. It is weakly concave in *Tyrannosaurus rex* (CM 9380; LACM 150167, RSM P2523.8).

**
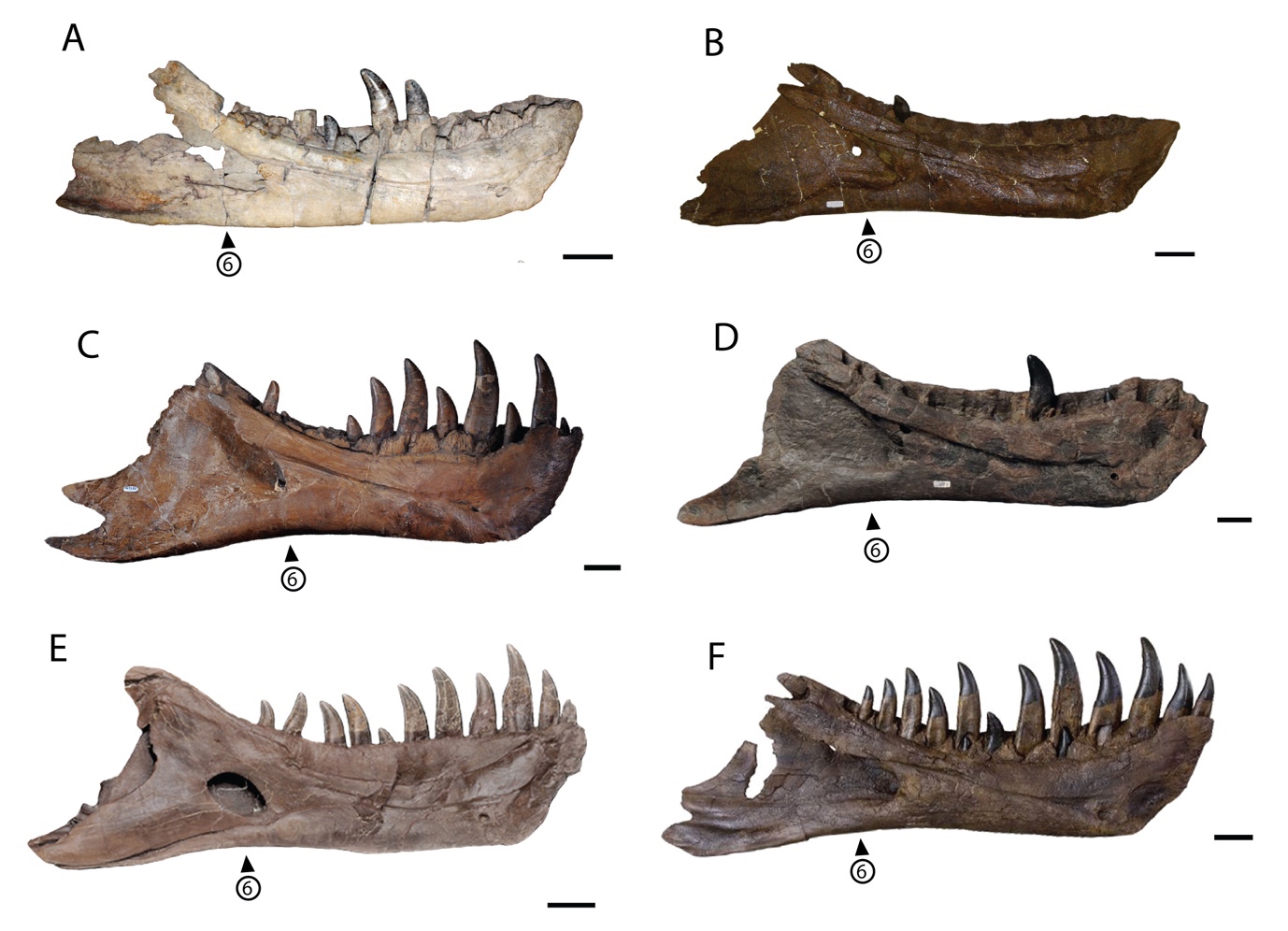
Fig. S16.** Dentary of **a**, *Tyrannosaurus mcraeensis* and **b**-**f**, *Tyrannosaurus rex* compared; a, NMMNH P-3698; **b**, MOR 1125; **c**, CM 9380 (holotype); **d**, LACM 150167; **e**, FMNH PR2081; **f**, BHI 3033. Scale = 10 cm.

(6) *Posterior of dentary deep and with a convex posteroventral margin*. The posteroventral margin of the dentary in *Tyrannosaurus mcraeensis* is shallow and strongly convex (Fig. S16), with a slightly convex ventral margin that curves up posteriorly such that the angular process is directed posterodorsally. This contrasts with the condition in *T. rex* specimens (AMNH 5027, BHI 3033; CM 9380; FMNH PR 2081; MOR 1125; LACM 23844; LACM 150167, RSM P2523.8), where the dentary is deep and concave below the back of the toothrow and the angular process extends posteroventrally. The sole exception is MOR 008; in this specimen the posteroventral margin is straight to convex, but the dentary is much deeper than in NMMNH P-3698, so the morphology seen in NMMNH P-3698 does not occur in any specimen of *T*. *rex*.

**
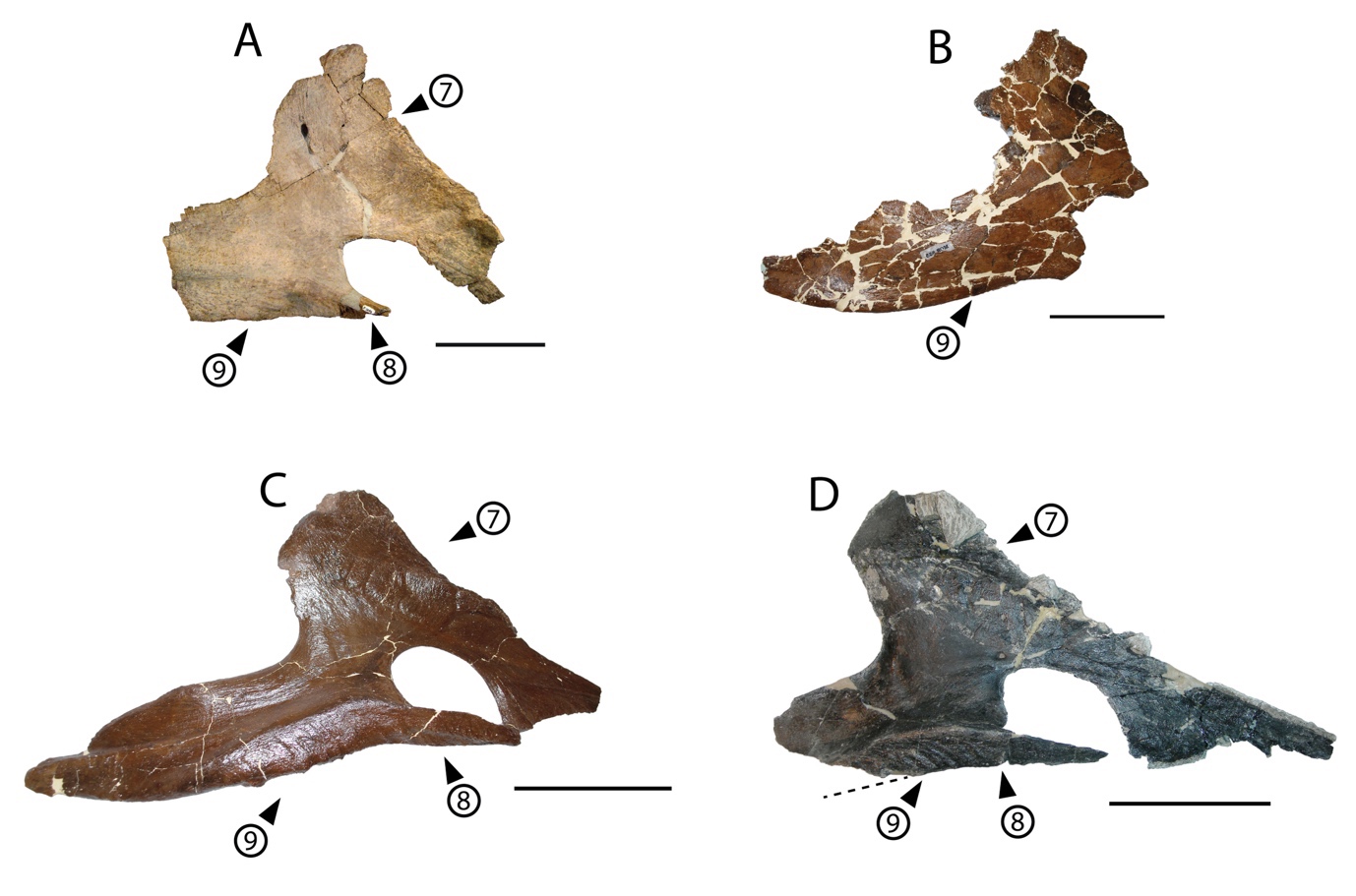
Fig. S17.** Splenial of **a**, *Tyrannosaurus mcraeensis* and **b**-**f**, *Tyrannosaurus rex* compared; **a**, NMMNH P-3698; **b**, MOR 555; **c**, MOR 1125, **d**, TMP 1981.6.1. Scale = 10 cm.

(7) *Splenial apex directed dorsally*. In *Tyrannosaurus mcraeensis* NMMNH P-3698, the apex of the splenial rises almost vertically relative to the ventral margin of the jaw. In *T. rex*, the apex is directed more posteriorly (AMNH 5027, BHI 3033, FMNH PR 2081, LACM 157776, MOR 008, MOR 1125, TMP 1981.6.1) (Fig. S17).

(8) *Shelf-like overhang of dentary by splenial anteroventral process*. In *Tyrannosaurus mcraeensis* NMMNH P-3698, the anteroventral process of the splenial has a distinct, shelf-like overhang where the splenial anteroventral process contacts the dentary (Fig. S17). In *T. rex*, this contact is a simple abutting contact via a broad, laterally facing facet (BHI 3033, MOR 1125; TMP 1981.6.1).

(9) *Posterior (angular) process of splenial directed posteriorly*. In *Tyrannosaurus mcraeensis* NMMNH P-3698, the posterior end of the splenial projects posteriorly (Fig. S17); it is directed more posteroventrally in *T. rex* (AMNH 5027, BHI 3033, FMNH PR 2081, LACM 157776, MOR 008, MOR 555, MOR 1125, TMP 1981.6.1)

**
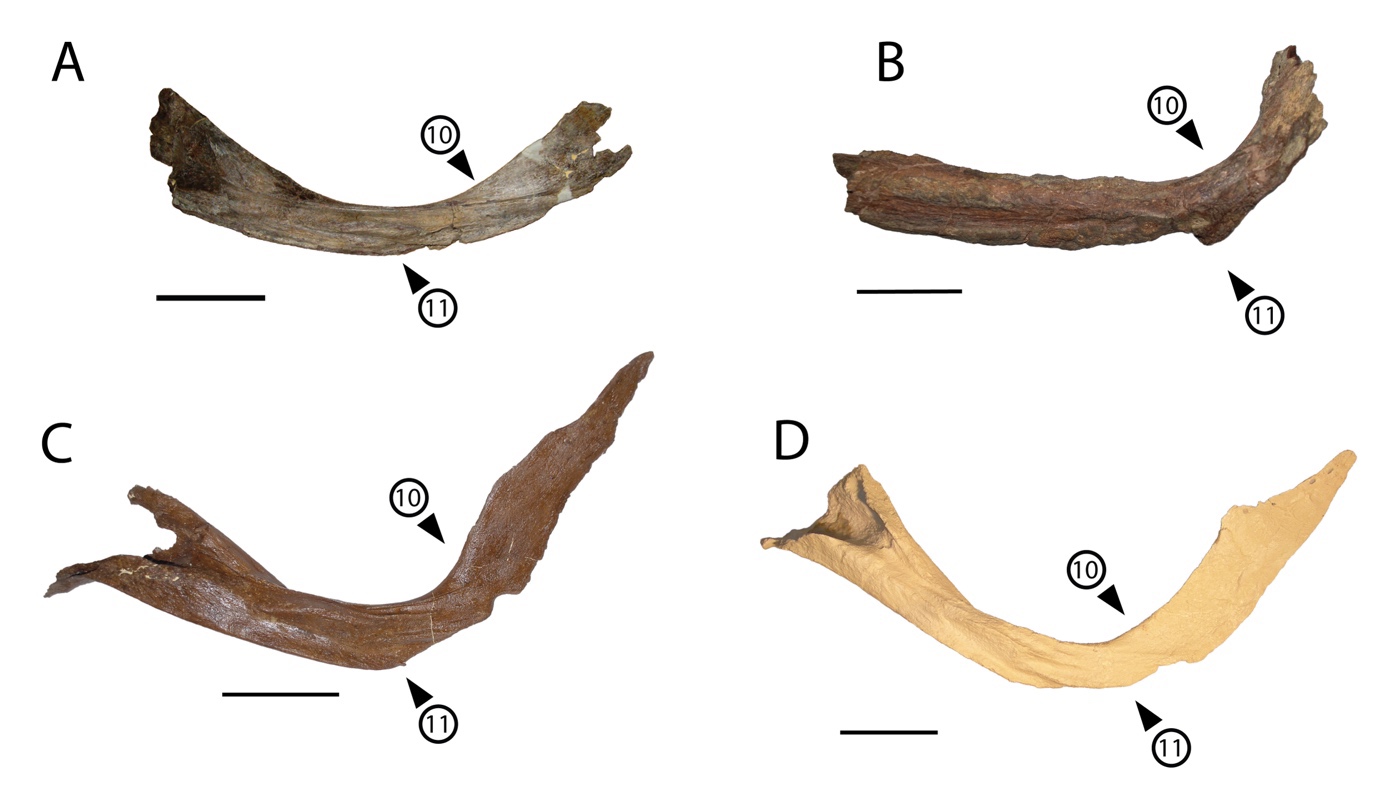
Fig. S18.** Prearticular of **a**, *Tyrannosaurus mcraeensis* and **b**-**d**, *Tyrannosaurus rex*. **a**, NMMNH P-3698; **b**, AMNH 5117; **c**, MOR 1125; **d**, BHI 3033 (cast). Scale = 10 cm.

(10) *Prearticular weakly bowed*. In NMMNH P-3698, the prearticular is weakly bowed where it approaches the back of the dentary and the splenial (Fig. S18). In *Tyrannosaurus* *rex*, the prearticular is strongly curved upward (AMNH 5029; AMNH 5117; BHI 3033; FMNH PR 2081; LACM 23844; MOR 008; MOR 1125).

(11) *Small ventral articulation between prearticular and angular*. In NMMNH P-3698, the prearticular has a narrow ventral shelf that articulated with the angular (Fig. S18). This shelf is enlarged in *Tyrannosaurus rex* (AMNH 5117; BHI 3033; FMNH PR 2081; MOR 1125), with a corresponding facet on the angular.

**
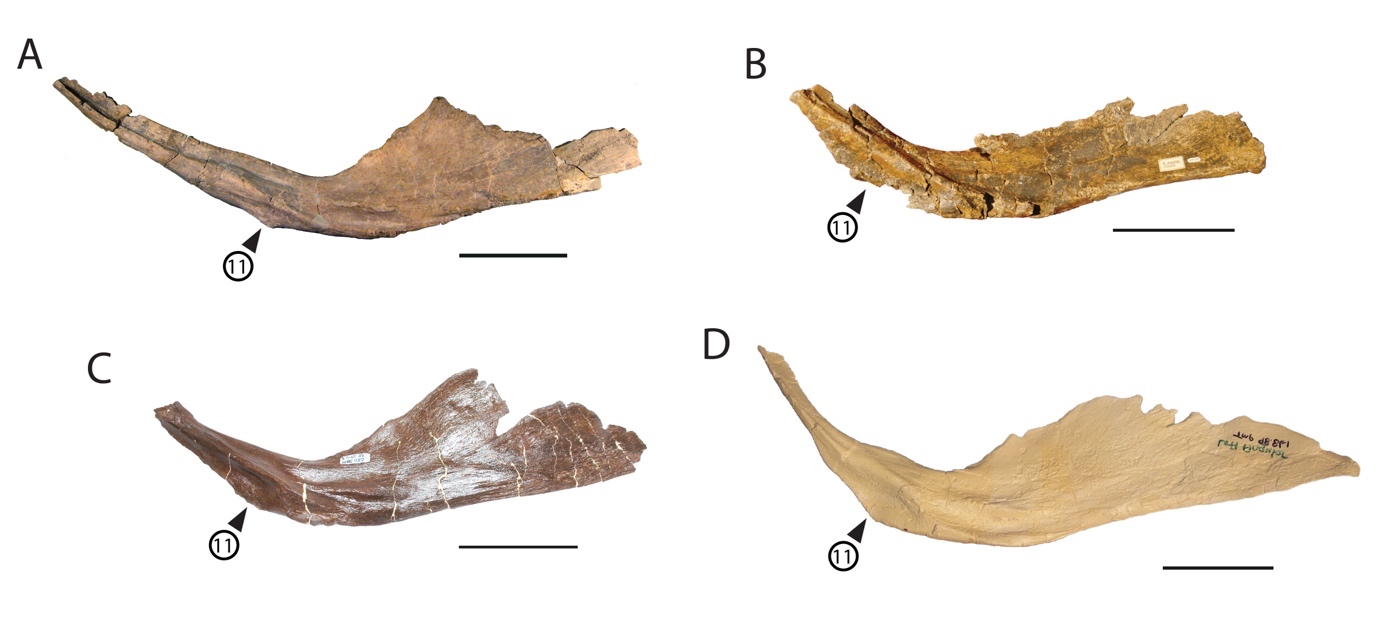
Fig. S19.** Angular of **a**, *Tyrannosaurus mcraeensis*, **b**-**d**, *Tyrannosaurus rex*. **a**, NMMNH P-3698; lateral view. **b**, RSM P2523.8; **c**, MOR 1125 (reversed), BHI 3033 (cast).

(11) *Small ventral articulation between angular and splenial*. The angular of NMMNH P-3698 has a weak anteroventral flange for the splenial. That of *T*. *rex* (BHI 3033; LACM 23844; MOR 1125, RSM P2523.8) is well-developed and forms a broad, strongly projecting ventral flange. This character is also seen in the corresponding articulation of the prearticular (Fig. S19).


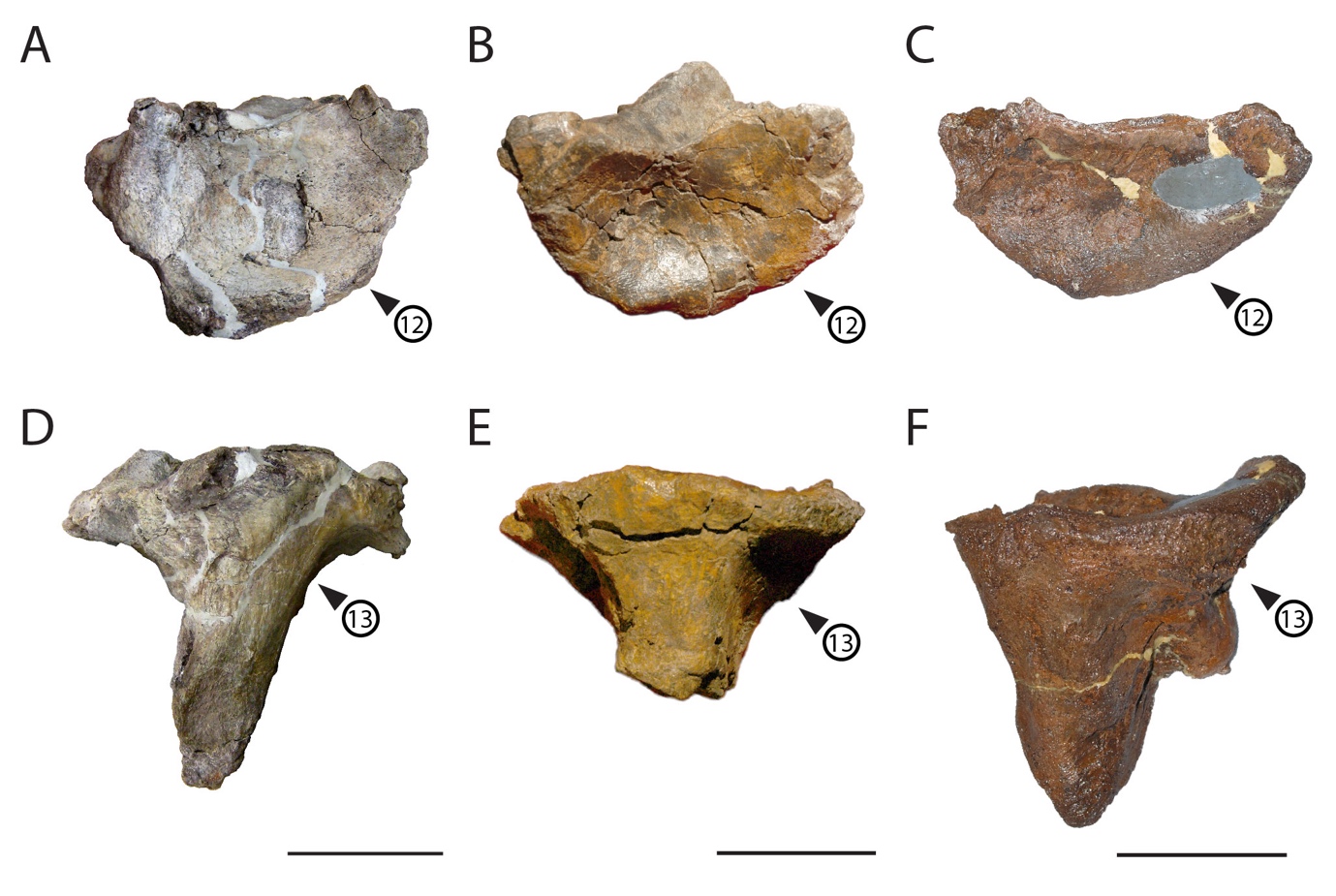


**Fig. S20.** Articular of **a**, *Tyrannosaurus mcraeensis*, **b**,**c**, *Tyrannosaurus rex* in posterior view; **d**, *Tyrannosaurus mcraeensis* and **e**,**f** *Tyrannosaurus rex* in ventral view. **a**,**d** NMMNH P-3698. **B**,**e** RSM P2523.8; **c**,**f** MOR 980.

(12) Retroarticular process deep and quadrangular in posterior view. The retroarticular process of NMMNH P-3698 is dorsoventrally deep and has an angular shape seen in posterior view (Fig. S20). That of *Tyrannosaurus rex* is mediolaterally broader and has a broadly rounded ventral margin (BHI 3044, MOR 008, MOR 980, RSM P2523.8).

(13) Angular T-shaped in ventral view. The angular is narrow anteriorly then strongly flares out posteriorly where it forms the retroarticular process in NMMNH P-3698. In *Tyrannosaurus rex* (BHI 3044, MOR 980, RSM P2523.8) the angular has a more triangular shape, gradually becoming wider posteriorly (Fig. S20).

1. **Phylogenetic and Biogeographic Analysis**

We estimated a time-calibrated phylogeny based on the 512-character matrix using the Mk model (Lewis, 2001) and the total-evidence approach of Ronquist et al. (Ronquist et al., 2012a) as described in recent papers (Pyron, 2011). We first estimated an uncalibrated topology with 28 characters specified as ordered. We then estimated strict-clock branch lengths for this topology as a constraint. From the linear regression of the calibrated vs. uncalibrated branch lengths, we took the inverse of the slope as the prior value for the independent gamma-rates model, exponentially distributed with mean = 4.4782. For the lognormal distribution on the clock-rate prior, we used the median potential root age (235Ma) divided by the median tree height from the strict-clock analysis (0.3274 subst. per site) for a mean of -6.576149 and a standard deviation of 0.01 (0.100753 in lognormal space) for a broad prior density. Other priors were placed on their default setting, including the fossilized-clock branch-length model, exp(10) for speciation rate, beta(1,1) for extinction and fossilization, sampling probability of 0.01, sampling strategy set to “diversity” to indicate maximal coverage of deep internal branches, a uniform prior of 213–253Ma on the root age, and a calibrated node-age prior. All analyses were performed in MrBayes 3.2.7a (Ronquist et al., 2012b), with 4 runs of 4 chains each run for 20 million generations, with ESS > 200 for all parameters and PSRF ~ 1 across runs and chains. We summarized the posterior distribution as a maximum clade credibility tree (Figure S21) calculated using the ‘paleotree’ package in R (Bapst, 2012).

We optimized biogeographic history across the summary tree by classifying species into 6 areas: Europe (A), South America (B), Asia (C), Appalachia (D), Northern Laramidia (E), and Southern Laramidia (F), the latter two separated by the northern borders of present-day Utah and Colorado as the demarcating boundary. We tested DIVA-like (Ronquist, 1997), BAYAREA-like (Landis et al., 2013), and DEC (Matzke, 2014) models in RASP (Yu et al., 2015). We also tested the “+J” jump-dispersal parameter despite recent criticisms (Ree and Sanmartín, 2018) which have recently been addressed (Matzke, 2022). Model testing revealed moderate support for DEC (AICc weight = 0.4; Table S2) and strong support for the “+J” parameter (*P =* 0.0001; Table S2), which we implemented for ancestral-range estimation (Figure S22, S23).

We estimate strong support for an Asian origin of Tyrannosauroidea and a Northern Laramidia origin of Tyrannosauridae, indicating Beringian dispersal into the New World and subsequent expansion into Southern Laramidia. A secondary re-colonization of Asia from Southern Laramidia occurred within Tyrannosaurini by the *Tarbosaurus* + *Zhuchengtyrannus* clade*,* the sister lineage to *Tyrannosaurus.* Subsequently, *Tyrannosaurus rex* expanded across Laramidia prior to the end Cretaceous, while *Tyrannosaurus mcraeensis* is known only from the type locality in Southern Laramidia. We visualized the timescale and biogeographic results using the ‘strap’ package in R (Bell and Lloyd, 2015).

Results of the time-calibrated analysis are in broad agreement with those of a cladistic analysis using equal-weights parsimony (Fig. S24) in TNT.

Inclusion of NMMNH P-3698 in an alternative matrix, Voris et al. (2020) (which is in turn derived from Carr, 2017) produces broadly similar results (Fig. S25) with *Tyrannosaurus mcraeensis* recovered as part of an unresolved polytomy with *Tarbosaurus* and *Tyrannosaurus rex*. The difference primarily stems from the addition of characters specific to the *Tyrannosaurus mcraeensis* + *Tyrannosaurus rex* clade in the present study, which improves resolution.

**Table S2.** Model-fitting results for 6 base models in RASP, indicating best fit of DEC+J.

| ***Model*** | ***LnL*** | ***numparams*** | ***d*** | ***e*** | ***j*** | ***AICc*** | ***AICc_wt*** |
| --- | --- | --- | --- | --- | --- | --- | --- |
| DEC | -95.75 | 2 | 0.0097 | 0.14 | 0 | 195.7 | 0.0009 |
| **DEC+J** | **-88.56** | **3** | **0.0035** | **0.29** | **0.056** | **183.6** | **0.4** |
| DIVALIKE | -92.62 | 2 | 0.012 | 0.28 | 0 | 189.5 | 0.021 |
| DIVALIKE+J | -89.01 | 3 | 0.0044 | 0.36 | 0.048 | 184.5 | 0.26 |
| BAYAREALIKE | -97.6 | 2 | 0.01 | 0.21 | 0 | 199.4 | 0.0001 |
| BAYAREALIKE+J | -88.77 | 3 | 0.0028 | 0.23 | 0.058 | 184 | 0.32 |

**Table S3.** Hypothesis-testing for the “+J” parameter, indicating significance for all model classes.

| ***alt*** | ***null*** | ***LnLalt*** | ***LnLnull*** | ***DFalt*** | ***DFnull*** | ***DF*** | ***Dstatistic*** | ***pval*** |
| --- | --- | --- | --- | --- | --- | --- | --- | --- |
| DEC+J | DEC | -88.56 | -95.75 | 3 | 2 | 1 | 14.38 | 0.0001 |
| DIVALIKE+J | DIVALIKE | -89.01 | -92.62 | 3 | 2 | 1 | 7.23 | 0.0072 |
| BAYAREALIKE+J | BAYAREALIKE | -88.77 | -97.6 | 3 | 2 | 1 | 17.67 | 2.60E-05 |

**Figure S21.** Maximum clade credibility tree from total-evidence dating, with branch lengths in time scaled relative to the end Cretaceous (~66Ma), and posterior probability node support (>0.95 considered “strong”). Tick marks on the scale bar indicate 5Ma intervals.


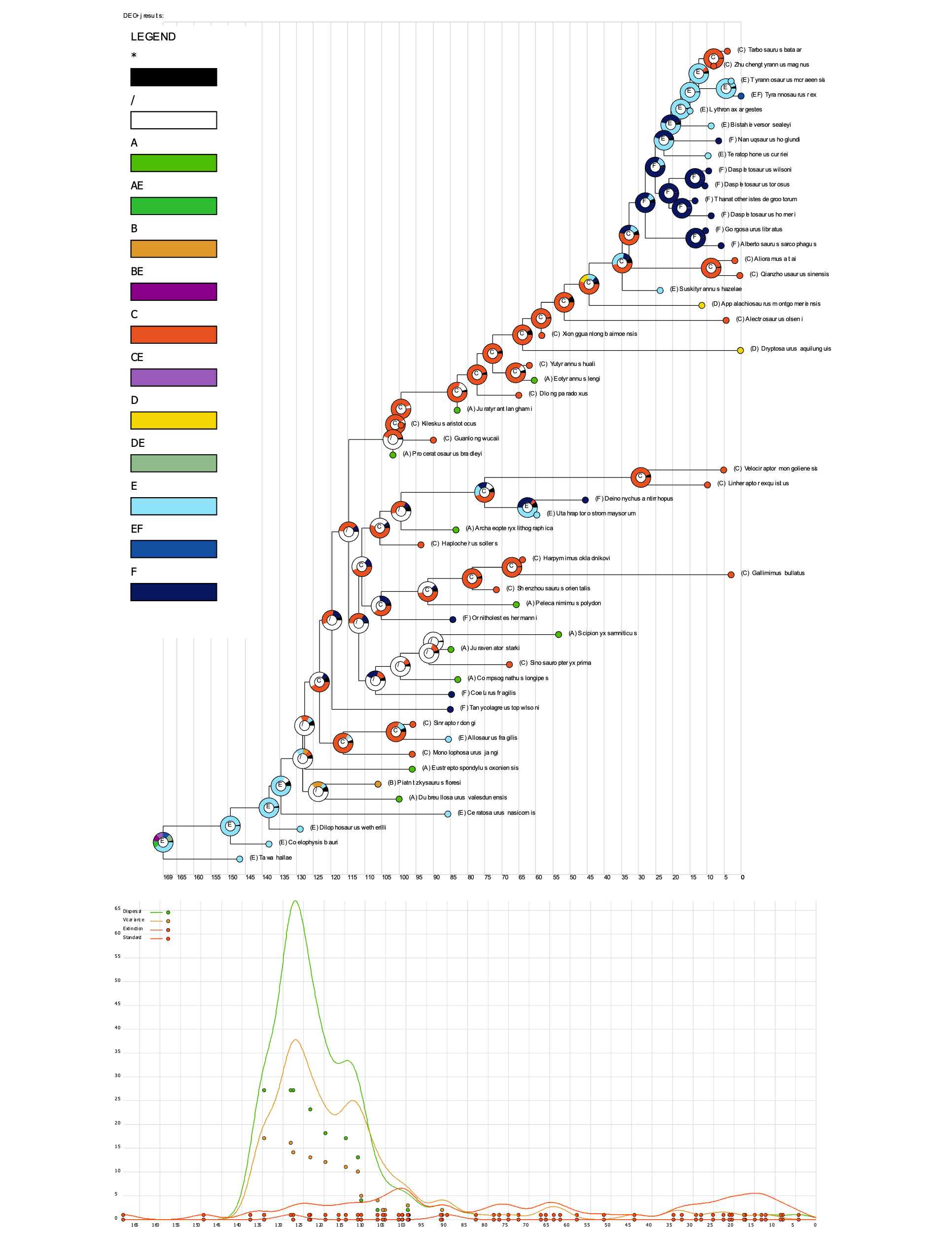


**Figure S22.** Full results from the DEC+J model estimated in RASP, showing Beringian dispersal of Tyrannosauridae into Northern Laramidia, evolution of Tyrannosaurini in southern Laramidia, and re-colonization of Asia from Southern Laramidia in the late Cretaceous. The timescale is offset to the end Cretaceous (~66 Ma), with tick marks every 10 Ma.

**Figure S23.** Expanded version of the figure from the main text, showing the most-likely state from the DEC+J ancestral-range estimation. Nodes without states had ambiguous reconstructions or exceeded the maximum allowable number of areas (2) observed in the tip states.

**Figure S24.** Relationships of *Tyrannosaurus mcraeensis* (NMMNH P-3698) within Theropoda. 50% majority rules consensus of the 10 most parsimonious trees of 55 taxa and 551 morphological characters, with a treelength of 1799 steps. CI = 0.377, RI = 0.756. Only nodes scoring less than 100 are indicated.

**Figure S25.** Relationships of *Tyrannosaurus mcraeensis* (NMMNH P-3698) using the Voris et al.(2020) matrix in TNT. Strict consensus of two most-parsimonious trees.

**References**

Amato, J.M., Mack, G.H., Jonell, T.N., Seager, W.R., Upchurch, G.R., 2017. Onset of the Laramide orogeny and associated magmatism in southern New Mexico based on U-Pb geochronology. Bulletin 129, 1209-1226.

Bapst, D.W., 2012. Paleotree: an R package for paleontological and phylogenetic analyses of evolution. Methods in Ecology and Evolution 3, 803-807.

Bell, M.A., Lloyd, G.T., 2015. strap: an R package for plotting phylogenies against stratigraphy and assessing their stratigraphic congruence. Palaeontology 58, 379-389.

Benson, R.B., Campione, N.E., Carrano, M.T., Mannion, P.D., Sullivan, C., Upchurch, P., Evans, D.C., 2014. Rates of dinosaur body mass evolution indicate 170 million years of sustained ecological innovation on the avian stem lineage. PLoS Biol 12.

Brochu, C.A., 2002. Osteology of *Tyrannosaurus rex*: insights from a nearly complete skeleton and high-resolution computed tomographic analysis of the skull. Journal of Vertebrate Paleontology, Memoirs 7, 1-138.

Brown, B., Kaisen, P.C., 1933. A gigantic ceratopsian dinosaur, Triceratops maximus, new species. American Museum novitates; no. 649.

Brusatte, S.L., Carr, T.D., Norell, M.A., 2012. The osteology of *Alioramus*, a gracile and long-snouted tyrannosaurid (Dinosauria: Theropoda) from the Late Cretaceous of Mongolia. Bulletin of the American Museum of Natural History 2012, 1-197.

Campione, N.E., Evans, D.C., 2011. Cranial growth and variation in edmontosaurs (Dinosauria: Hadrosauridae): implications for latest Cretaceous megaherbivore diversity in North America. PLoS ONE 6, e25186.

Campione, N.E., Evans, D.C., 2020. The accuracy and precision of body mass estimation in non‐avian dinosaurs. Biological Reviews 95, 1759-1797.

Carr, T.D., Williamson, T.E., 2010. *Bistahieversor sealeyi*, gen. et sp. nov., a new tyrannosauroid from New Mexico and the origin of deep snouts in Tyrannosauroidea. Journal of Vertebrate Paleontology 30, 1-16.

Carr, T.D., Varricchio, D.J., Sedlmayr, J.C., Roberts, E.M., Moore, J.R., 2017. A new tyrannosaur with evidence for anagenesis and crocodile-like facial sensory system. Scientific Reports 7, 1-11.

Carr, T.D., Napoli, J.G., Brusatte, S.L., Holtz, T.R., Hone, D.W., Williamson, T.E., Zanno, L.E., 2022. Insufficient Evidence for Multiple Species of *Tyrannosaurus* in the Latest Cretaceous of North America: A Comment on “The Tyrant Lizard King, Queen and Emperor: Multiple Lines of Morphological and Stratigraphic Evidence Support Subtle Evolution and Probable Speciation Within the North American Genus *Tyrannosaurus*”. Evolutionary Biology, 1-15.

Currie, P.J., 2003. Cranial anatomy of tyrannosaurid dinosaurs from the Late Cretaceous of Alberta, Canada. Acta Palaeontologica Polonica 48, 191-226.

Dalman, S.G., Lucas, S.G., 2017. On the Dentary in the Fukui Prefectural Dinosaur Museum of Gorgosaurus libratus (Theropoda: Tyrannosauridae) from the Dinosaur Park formation (Upper Cretaceous) of Alberta, Canada. Memoir of the Fukui Prefectural Dinosaur Museum 16, 17-27.

Dalman, S.G., Lucas, S.G., Jasinski, S.E., Longrich, N.R., 2022. *Sierraceratops turneri*, a new chasmosaurine ceratopsid from the Hall Lake Formation (Upper Cretaceous) of south-central New Mexico. Cretaceous Research 130, 105034.

Erickson, G.M., Olson, K.H., 1996. Bite marks attributable to *Tyrannosaurus rex*: preliminary description and implications. 16, 175-178.

Fiorillo, A.R., Tykoski, R.S., 2014. A diminutive new tyrannosaur from the top of the world. PLoS One 9, e91287.

Gignac, P.M., Erickson, G.M., 2017. The biomechanics behind extreme osteophagy in *Tyrannosaurus rex*. Scientific Reports 7, 1-10.

Hone, D.W., Wang, K., Sullivan, C., Zhao, X., Chen, S., Li, D., Ji, S., Ji, Q., Xu, X., 2011. A new, large tyrannosaurine theropod from the Upper Cretaceous of China. Cretaceous Research 32, 495-503.

Hurum, J.H., Sabath, K., 2003. Giant theropod dinosaurs from Asia and North America: skulls of *Tarbosaurus bataar* and *Tyrannosaurus rex* compared. Acta Palaeontologica Polonica 48.

Kirkland, J.I., Hernández-Rivera, R., Gates, T., Paul, G.S., Nesbitt, S., Serrano-Brañas, C.I., Garcia-de la Garza, J.P., 2006. Large hadrosaurine dinosaurs from the latest Campanian of Coahuila, Mexico. New Mexico Museum of Natural History and Science Bulletin 35, 299-315.

Landis, M.J., Matzke, N.J., Moore, B.R., Huelsenbeck, J.P., 2013. Bayesian analysis of biogeography when the number of areas is large. Systematic Biology 62, 789-804.

Lehman, T.M., McDowell, F.W., Connelly, J.N., 2006. First isotopic (U-PB) age for the Late Cretaceous *Alamosaurus* vertebrate fauna of West Texas, and its significance as a link between two faunal provinces. Journal of Vertebrate Paleontology 26, 922-928.

Lehman, T.M., Wick, S.L., Barnes, K.R., 2017. New specimens of horned dinosaurs from the Aguja Formation of West Texas, and a revision of *Agujaceratops*. Journal of Systematic Palaeontology 15, 641-674.

Leslie, C.E., Peppe, D.J., Williamson, T.E., Heizler, M., Jackson, M., Atchley, S.C., Nordt, L., Standhardt, B., 2018. Revised age constraints for Late Cretaceous to early Paleocene terrestrial strata from the Dawson Creek section, Big Bend National Park, west Texas. GSA Bulletin 130, 1143-1163.

Lewis, P.O., 2001. A likelihood approach to estimating phylogeny from discrete morphological character data. Systematic biology 50, 913-925.

Loewen, M.A., Sampson, S.D., Lund, E.K., Farke, A.A., Aguillón-Martínez, M.C., de Leon, C.A., Rodríguez-de la Rosa, R.A., Getty, M.A., Eberth, D.A., 2010. Horned dinosaurs (Ornithischia: Ceratopsidae) from the Upper Cretaceous (Campanian) Cerro del Pueblo Formation, Coahila, Mexico, in: Ryan, M.J., Chinnery, B.J., Eberth, D.A. (Eds.), New Perspectives on Horned Dinosaurs: The Royal Tyrrell Museum Ceratopsian Symposium. Indiana University Press, Bloomington, pp. 99-116.

Loewen, M.A., Irmis, R.B., Sertich, J.J., Currie, P.J., Sampson, S.D., 2013. Tyrant dinosaur evolution tracks the rise and fall of Late Cretaceous oceans. PLoS ONE 8, e79420.

Longrich, N.R., Horner, J.R., Erickson, G.M., Currie, P.J., 2010. Cannibalism in *Tyrannosaurus rex*. PLoS ONE 5(10), e13419.

Lucas, S.G., Nelson, W.J., Krainer, K., Elrick, S.D., 2019. The Cretaceous System in central Sierra County, New Mexico. New Mexico Geology 41, 3-39.

Matzke, N.J., 2014. Model selection in historical biogeography reveals that founder-event speciation is a crucial process in island clades. Systematic biology 63, 951-970.

Matzke, N.J., 2022. Statistical comparison of DEC and DEC+ J is identical to comparison of two ClaSSE submodels, and is therefore valid. Journal of Biogeography 49, 1805-1824.

Nesbitt, S.J., Denton, R.K., Loewen, M.A., Brusatte, S.L., Smith, N.D., Turner, A.H., Kirkland, J.I., McDonald, A.T., Wolfe, D.G., 2019. A mid-Cretaceous tyrannosauroid and the origin of North American end-Cretaceous dinosaur assemblages. Nature Ecology & Evolution 3, 892-899.

Osborn, H.F., 1905. *Tyrannosaurus* and other Cretaceous carnivorous dinosaurs. Bulletin of the American Museum of Natural History 35, 733-771.

Paul, G.S., Persons, W.S., Van Raalte, J., 2022. The Tyrant Lizard King, Queen and Emperor: Multiple Lines of Morphological and Stratigraphic Evidence Support Subtle Evolution and Probable Speciation Within the North American Genus *Tyrannosaurus*. Evolutionary Biology 49, 156-179.

Persons IV, W.S., Currie, P.J., Erickson, G.M., 2020. An older and exceptionally large adult specimen of *Tyrannosaurus rex*. The Anatomical Record 303, 656-672.

Pyron, R.A., 2011. Divergence time estimation using fossils as terminal taxa and the origins of Lissamphibia. Systematic biology 60, 466-481.

Ramírez-Velasco, A.A., 2022. Phylogenetic and biogeography analysis of Mexican hadrosauroids. Cretaceous Research, 105267.

Ree, R.H., Sanmartín, I., 2018. Conceptual and statistical problems with the DEC+ J model of founder‐event speciation and its comparison with DEC via model selection. Journal of Biogeography 45, 741-749.

Ronquist, F., 1997. Dispersal-vicariance analysis: a new approach to the quantification of historical biogeography. Systematic biology 46, 195-203.

Ronquist, F., Klopfstein, S., Vilhelmsen, L., Schulmeister, S., Murray, D.L., Rasnitsyn, A.P., 2012a. A total-evidence approach to dating with fossils, applied to the early radiation of the Hymenoptera. Systematic Biology 61, 973-999.

Ronquist, F., Teslenko, M., Van Der Mark, P., Ayres, D.L., Darling, A., Höhna, S., Larget, B., Liu, L., Suchard, M.A., Huelsenbeck, J.P., 2012b. MrBayes 3.2: efficient Bayesian phylogenetic inference and model choice across a large model space. Systematic Biology 61, 539-542.

Smith, J.B., 2005. Heterodonty in *Tyrannosaurus* *rex*: implications for the taxonomic and systematic utility of theropod dentitions. Journal of Vertebrate Paleontology 25, 865-887.

Voris, J.T., Zelenitsky, D.K., Therrien, F., Currie, P.J., 2019. Reassessment of a juvenile *Daspletosaurus* from the Late Cretaceous of Alberta, Canada with implications for the identification of immature tyrannosaurids. Scientific Reports 9, 1-10.

Voris, J.T., Therrien, F., Zelenitsky, D.K., Brown, C.M., 2020. A new tyrannosaurine (Theropoda: Tyrannosauridae) from the Campanian Foremost Formation of Alberta, Canada, provides insight into the evolution and biogeography of tyrannosaurids. Cretaceous Research 110, 104388.

Wick, S.L., Lehman, T.M., 2013. A new ceratopsian dinosaur from the Javelina Formation (Maastrichtian) of West Texas and implications for chasmosaurine phylogeny. Naturwissenschaften.

Yu, Y., Harris, A.J., Blair, C., He, X., 2015. RASP (Reconstruct Ancestral State in Phylogenies): a tool for historical biogeography. Molecular Phylogenetics and Evolution 87, 46-49.
