# Supplementary material for: A giant tyrannosaur from the Campanian–Maastrichtian of southern North America and the evolution of tyrannosaurid gigantism
Source: Sci Rep. 2024 Jan 11;14:22124. doi: 10.1038/s41598-023-47011-0 (PMC10784284; doi:10.1038/s41598-023-47011-0)
Supplement: Supplementary file 2 — Supplementary Information 2. [file 41598_2023_47011_MOESM2_ESM.pdf]

Character list for phylogenetic analysis.

The 512 characters (334 cranial and 178 postcranial) used in the phylogenetic analysis are listed below. This matrix is derived from Loewen et al. 2013 and last appeared in Nesbitt et al. 2019. Recent previous usage of characters is indicated by citations in parentheses. Characters presented in Brusatte et al., 2009 were largely derived from Carr and Williamson, 2010, which was in press at the time. Many of the characters of Li et al. (2009); Sereno et al. (2009); Brusatte et al. (2009); and Carr and Williamson (2010) are restated in Brusatte et al. (2010). Citations previous to 2009 are generally not included, as many are reviewed in Sereno and Brusatte (2009). Taxa are scored for the adult condition wherever possible. New characters are indicated in red. Ordered characters are indicated as **ORDERED** and are based on either ontogenetic trajectory within a species of tyrannosaur or on inclusion of a character state within another. Ordered characters (a total of 27 include: 13, 39, 43, 46, 70, 94, 96, 100, 102, 108, 110, 137, 147, 149, 167, 188, 202, 207, 272, 274, 276, 281, 291, 406, 489, 491, and 508.

Skull

1. Naris, size:
   (0) – small, less than half of the length of the antorbital fenestra
   (1) – large, greater than half but still less than the length of the antorbital fenestra
   (2) – huge, approaching the length of the antorbital fenestra
   (Loewen et al., 2013:1 now run as unordered)
2. Naris, posterior margin position:
   (0) – anterior to antorbital fossa
   (1) – posterior to or even with the antorbital fossa, but anterior to maxillary fenestra
   (2) – posterior to antorbital fossa, and posterior to the anterior border of the maxillary fenestra
   (Modified from Chiappe et al., 1998; Li et al., 2009:23; modified from Loewen et al., 2013:2 now run as unordered)
3. Rostrum length compared to posterior skull length (measured from the anterior premaxilla to midpoint of the lacrimal bar divided by basal skull length to end of quadrate):
   (0) – longirostran, rostrum more than 0.65
   (1) – rostrum 0.55 - 0.65 of posterior skull
   (2) – brevirostrine, rostrum 0.4 - 0.55
   (Loewen et al., 2013:3)
4. Orbit, shape:
   (0) – round
   (1) – orbit more than 10% taller than wide but less than twice as tall
   (2) – dorsoventrally elongate orbit twice as tall as wide
   (Modified from Li et al., 2009:2)
5. Orbit size:
   (0) – huge, anterioposterior length at least 3 times the anteroposterior length of laterotemporal fenestra
   (1) – small, anterioposterior length less than 3 times the anteroposterior length of laterotemporal fenestra
   (Loewen et al., 2013:5)
6. Orbit orientation:
   (0) – laterally directed, orbit is directed parallel to sagittal plane
   (1) – sublaterally, orbit is directed from 10 to 15 degrees from sagittal plane
   (2) – anteriorly directed, orbit is directed anteriorly at greater than 20 degrees from sagittal plane
7. Laterotemporal fenestra, posterior bar:
   (0) – ascending rami of quadrate and quadratojugal meet squamosal to form a straight posterior border
   (1) – angled (kinked) posterior border of the laterotemporal fenestra in lateral view
   (Li et al., 2009:254)

8. Lateral temporal fenestra, orientation of long axis relative to long axis of orbit:
    (0) – posterodorsally directed long axis of laterotemporal fenestra compared to long axis of orbit
    (1) – the long axis of the orbit and laterotemporal fenestra are approximately parallel
    (Modified from Brusatte et al., 2010:5)
9. Skull width vs skull length:
    (0) – narrow, 37% or less than premaxilla-quadrate length
    (1) – 40% - 58% of premaxilla-quadrate length
    (2) – wide, more than 63% of premaxilla-quadrate length
    (Modified from Holtz, 2001; Currie et al., 2003)
10. Snout width, mediolateral width of the snout at the level of the posterior-most maxillary tooth:
    (0) – twice or less the width of nasals
    (1) –approximately three times the width of the nasals
    (Holtz, 2001; Currie et al., 2003; Brusatte et al., 2009:2)
11. Skull height as a ratio of maximum skull height in lateral view to skull length (premaxilla-occipital condyle length):
    (0) – short, less than 30%
    (1) – tall, greater than 35%
    (Modified from Kurzanov, 1976; Brusatte et al., 2009:3)
12. Skull, anteroposterior length compared to the length of the dorsal vertebral series:
    (0) – skull short, less than 40% of the length of the dorsal vertebrae
    (1) – skull long, greater than 40% of the length of the dorsal vertebrae
    (Brusatte et al., 2010:3)

Premaxilla

13. Premaxilla, premaxillary symphasis, overall shape in dorsal view:
    (0) – acute, V-shaped
    (1) – rounded, U-shaped
    (2) – transverse, anteriorly facing
    ORDERED
    (Modified from Li et al., 2009:24)
14. Premaxilla, maxillary process orientation in dorsal view:
    (0) – laterally (and resultantly widely visible in lateral view)
    (1) – rostrolaterally (facing almost equally dorsally and laterally)
    (2) – anteriorly (and resultantly mostly hidden in lateral view)
    (Brusatte et al., 2010:9)
15. Anterior margin of the premaxillary body in lateral view:
    (0) – inclined and extends caudodorsally
    (1) – or extends nearly vertically
    (Modified from Brusatte et al., 2009:5)
16. Premaxilla, shape of anterior margin:
    (0) – smoothly curved
    (1) – discrete inflection point between nearly vertical anterior region and more horizontal dorsal region
    (Modified after Brusatte et al., 2010:13)
17. Premaxilla, nasal processes divergence:
    (0) – nasal processes are appressed
    (1) – nasal processes divergent with medial nasal process intervening in between
    (Carpenter et al., 2005; Brusatte et al., 2009:6)
18. Premaxilla, subnarial process length:
    (0) – subnarial process absent
    (1) – present but much shorter than narial process
    (2) – subequal to length of narial process
    (3) – much longer than narial process
    (Loewen et al., 2013:18)

19. Large foramen in the lateral surface of the base of the nasal process of the premaxilla:
    (0) – absent
    (1) – present
    (Modified from Brusatte et al., 2009:7)
20. Premaxilla, form of narial fossa ventral to internal naris:
    (0) – shallowly excavated
    (1) – deeply excavated, anterior margin invaginated as a deep groove
    (Modified after Brusatte et al., 2010:11)
21. Premaxilla, extent of narial fossa:
    (0) – limited to region immediately ventral to external naris
    (1) – extensive, covers more than two thirds of the depth of the body of premaxilla near the posterior end
    (Brusatte et al., 2010:12)
22. Premaxilla, main body, dorsoventral depth:
    (0) – shallow, less than or equal to anteroposterior length
    (1) – between 1-1.9 times anteroposterior length
    (2) – deep, greater than 2 times anteroposterior length
    (Brusatte et al., 2010:8)
23. Premaxillary interdental plates:
    (0) – fused
    (1) – unfused
    (Brusatte et al., 2009:12)
24. Premaxilla, position of palatal process:
    (0) – immediately above interdental plates
    (1) – separated from interdental plates by deep lingual surface of premaxilla
    (Brusatte et al., 2010:14)

Maxilla

25. Maxilla, constriction between articulated premaxillae and maxillae:
    (0) – absent
    (1) – present
    (Rauhut, 2003; Smith et al., 2007:21)
26. Maxilla and premaxilla contact along alveolar margins:
    (0) – in contact at alveolar magins
    (1) – alveolar margins do not contact
    (Tykoski, 2005; Smith et al., 2007:22)
27. Maxilla, orientation of the maxillae towards each other in dorsal view:
    (0) – acutely angled
    (1) – subparallel
    (Harris, 1998; Smith et al., 2007:23)
28. Maxilla, anterior margin, premaxillary suture on body of maxilla in lateral view:
    (0) – sloping
    (1) – vertical
    (Modified from Brusatte et al., 2009:22)
29. Maxilla, anterior process possesses concave step in anterior margin of maxilla:
    (0) – absent
    (1) – present shallow step with relatively short anterior ramus
    (2) – present deep step with long anterior ramus
    (Modified from Brusatte et al 2010:30)
30. Maxilla, pneumatic excavation/antrum in maxillary anterior process:
    (0) – absent
    (1) – present
    (Sereno et al., 1994; Smith et al., 2007:31)

31. Maxilla, vertical height at inflection point of anterior end of antorbital fenestra versus total height:
    (0) – short, less than 40% total height
    (1) – from 45% to 55% total height
    (2) – tall, more than 60% total height
    (Loewen et al., 2013:31)
32. Maxilla, promaxillary fenestra:
    (0) – absent
    (1) – present
    (Russell, 1970)
33. Maxillary fenestra:
    (0) – absent
    (1) – small, fenestra occupies less than half of the depressed area between the anterior margins of the antorbital fossa and antorbital fenestra
    (2) – large, takes up greater than half up to 3/4 of the space between the anterior margins of the antorbital fenestra and fossa
    (3) – greater than 3/4 of the length of the eyeball-bearing portion of the orbit
    (Modified from Gauthier, 1986; Li et al., 2009:29; Sereno et al., 2009: 1; Carr and Williamson 2010: 17; and Brusatte et al., 2010)
34. Maxilla, maxillary fenestra, shape of posterior border:
    (0) – round or ovoid
    (1) – subrectangular or squared corner
    (Loewen et al., 2013:34)
35. Maxilla, maxillary fenestra, location:
    (0) – posterior to the anterior margin of the antorbital fossa
    (1) – partially overlapped laterally by the anterior margin of the antorbital fossa
    (Carr and Williamson 2010: 15, 16; Sereno et al., 2009: 12; Brusatte et al., 2010:17)
36. Maxilla, maxillary fenestra, location:
    (0) – dorsal to ventral margin of antorbital fossa
    (1) – abuts ventral margin of antorbital fossa
    (Sereno et al. 2009: 13; Brusatte et al., 2010:18)
37. Maxilla, secondary posterior maxillary fenestra within interfenestral strut:
    (0) – absent
    (1) – present
    (Loewen et al., 2013:37)
38. Maxilla, elongate secondary fossa dorsal to maxillary fenestra inside antorbital fossa on the lateral surface of the maxilla:
    (0) – absent
    (1) – present
    (Loewen et al., 2013:38)
39. Maxilla, form of contact with nasal in subadult to adult specimens:
    (0) – smooth
    (1) – weakly scalloped
    (2) – deeply scalloped with interlocking transverse ridges on both elements
    ORDERED
    (Sereno et al. 2009: 15; Brusatte et al., 2010:31)
40. Maxilla, form of external subcutaneous surface texturing:
    (0) – random foramina and shallow grooves and ridges
    (1) – deep, prominent, dorsoventrally trending grooves and ridges
    (Brusatte et al., 2010:32)
41. Maxilla, neurovascular foramina, form:
    (0) – foramina absent
    (1) – primary row of foramina continues as a row posteriorly
    (2) – transitions into a sharp groove, paralleling the antorbital fossa rim, posteriorly
    (Modified from Brusatte et al., 2010:36)

42. Maxilla, antorbital fossa, extent on main body under midpoint of antorbital fenestra:
    (0) – covers more than 65% of the depth of the main body beneath the midpoint of the antorbital fenestra
    (1) – covers 50% to 60% of the depth of the main body beneath the midpoint of the antorbital fenestra
    (2) – covers much less than 45% of the depth of the main body beneath the midpoint of the antorbital fenestra
    (Modified from Brusatte et al., 2010:37)
43. Maxilla, ventral margin in lateral view:
    (0) – straight
    (1) – slightly convex
    (2) – highly convex
    ORDERED
    (Modified from Sereno et al. 2009: 14; Carr and Williamson 2010: 30; Brusatte et al., 2010:28)
44. Maxilla, dorsal alveolar ridge or palatal shelf of maxilla dorsal to interdental plates:
    (0) – unexpanded mediolaterally
    (1) – broad mediolateral expansion forming shelf subequal to or wider than alveolar body
    (Loewen et al., 2013:44)
45. Maxilla, broad mediolaterally expanded dorsal alveolar ridge or palatal shelf, ventral border:
    (0) – covex below palatal suture
    (1) – concave below palatal suture
    (Loewen et al., 2013:45)
46. Maxilla, depressor pits for dentary teeth in dorsal alveolar ridge or palatal shelf of maxilla dorsal to interdental plates:
    (0) – no pits
    (1) – shallow pits along most of the ventral surface of palatal shelf of maxilla, may have deep anterior pits
    (2) – deep pits along ventral surface of palatal shelf of maxilla
    ORDERED
    (Loewen et al., 2013:46)

Nasal

47. Nasal, constriction just posterior to subnarial processes, in mediolateral width at posterior end of naris:
    (0) – unconstricted
    (1) – constricted, but less than 30% of the width of widest point of nasal
    (2) – constricted, to less than 50% of the width of widest point of nasal
    (Modified from Carr and Williamson 2010: 41; Brusatte et al 2010:42; Loewen et al., 2013:47 now run as unordered)
48. Nasals, fusion along the midline:
    (0) – unfused along entire length
    (1) – at least partially fused
    (Holtz, 2001; Li et al., 2009:263)
49. Nasal, crest (or crests):
    (0) – absent
    (1) – midline crest present
    (2) – bilateral crests present along lateral margins of nasals
    (Li et al., 2009:264)
50. Nasal, crest fenestration:
    (0) – not fenestrate
    (1) – fenestrate
    (Modified from Brusatte et al., 2009:45)
51. Nasal, crest, dorsal wing of lacrimal wraps onto side of nasal crest:
    (0) – absent
    (1) – present
    (Smith et al., 2007; Li et al., 2009:290)

52. Nasal, dorsal surface ornamentation (excluding crests):
    (0) – smooth
    (1) – ornamented
    (Holtz, 2001; Li et al., 2009:296)
53. Nasal, dorsal ornamentation:
    (0) – irregular rugosities
    (1) – midline hornlets
    (Loewen et al., 2013:53)
54. Nasal, dorsal surface of the nasals (excluding crest morphology):
    (0) – flat for most of length
    (1) – dorsally convex
    (Modified from Currie et al., 2003; Brusatte et al., 2009:32)
55. Nasal, dorsal surface of the posterior end of the nasal (frontal process only):
    (0) – flat
    (1) – convex
    (2) – concave
    (Modified from Brusatte et al., 2009:42)
56. Nasal, dorsal surface in dorsal view, mediolateral expansion of anterior end, anterior to the maxillary fenestra:
    (0) – absent
    (1) – present
    (Loewen et al., 2013:56)
57. Nasal, in dorsal view, posterior end:
    (0) – the same width as the rest of the element
    (1) – restricted, posterior 1/3 of element narrower than the anterior 2/3
    (2) – expanded, posterior 1/3 of element wider than the anterior 2/3
    (Loewen et al., 2013:57)
58. Nasal, dorsal surface in lateral view (excluding crests):
    (0) – straight or convex, relatively flat
    (1) – concave with ventral deflection
    (Loewen et al., 2013:58)
59. Nasal, lateral wing for articulation with lacrimal:
    (0) – absent
    (1) – present
    (Modified from Smith et al., 2007:290; Carr and Williamson 2010: 40; Sereno et al. 2009:4; Brusatte et al 2010)
60. Nasal, frontal suture shape:
    (0) – simple wedge (V-shaped)
    (1) – W-shaped
    (2) – transverse or gentle U-shaped
    (Loewen et al., 2013:60)

Lacrimal

61. Lacrimal, shape in lateral view:
    (0) – inverted “L” shaped, anterior and ventral process at right angles
    (1) – “7” shaped, acute angle between anterior and ventral processes
    (Modified from Carr and Williamson 2010: 55; Brusatte et al., 2010: 48)
62. Lacrimal, anterior process length:
    (0) – longer than ventral process
    (1) – from 90% to 70% the length of the ventral process
    (2) – short less than 65% ventral process length
    (Modified from Sereno et al., 1998; Smith et al., 2007:56)

63. Lacrimal, ornamentation in adult individuals:
    (0) – absent
    (1) – manifest only as surface rugosity
    (2) – dorsal crest
    (Modified from Sereno et al. 2009: 18; Carr and Williamson 2010: 56; Brusatte et al., 2010: 49)
64. Lacrimal, dorsal crest, height in lateral view:
    (0) – low, less than the width of the anterior/posterior length of the descending process
    (1) – relatively tall, at least as tall as the anterior/posterior length of the descending process
    (New Character)
65. Lacrimal, dorsal crest, cross-section in anterior view:
    (0) – pinched or squared
    (1) – rounded
    (Loewen et al., 2013:65)
66. Lacrimal, dorsal crest, shape in lateral view:
    (0) – sharp, with crest forming distinct peak
    (1) – rounded
    (2) – rounded peak
    (Loewen et al., 2013:66)
67. Lacrimal, dorsal crest, position of the apex of the ornamentation in lateral view:
    (0) – apex anterior to posterior edge of antorbital fenestra
    (1) – apex posterior to posterior edge of antorbital fenestra
    (Modified from Li et al., 2009:52)
68. Lacrimal, posterior dorsal process:
    (0) – absent, or short
    (1) – present, long and gracile
    (2) – present, well-developed and robust
    (Loewen et al., 2013:68)
69. Lacrimal, cornual boss on posterodorsal process:
    (0) – absent
    (1) – present, boss forms ridge or shelf so that ridge and rounded crest are sigmoidal in dorsal view
    (2) – present, forms massive rounded boss
    (Loewen et al., 2013:69)
70. Lacrimal, extent of pneumaticity:
    (0) – absent
    (1) – limited, partially hollows bone in the region where the anterior and ventral rami meet
    (2) – extensive, completely hollows bone
    ORDERED
    (Sereno et al. 2009: 16; Brusatte et al 2010: 52)
71. Lacrimal, accessory pneumatic recess on anterior process of lacrimal:
    (0) – absent
    (1) – present
    (Modified from Brusatte et al., 2009:60)
72. Lacrimal, position of accessory pneumatic recess:
    (0) – posterior, much closer to ventral process than anterior end of anterior process
    (1) – anterior, much closer to anterior end of anterior process than ventral process
    (Modified from Brusatte et al., 2009:61)
73. Lacrimal, pneumatic recess opening internally onto anteriomedial surface of bone inside antorbital fossa and below anterior process and lacrimal vacuity:
    (0) – absent
    (1) – present
    (Carr and Williamson 2010: 68; Brusatte et al 2010:57)
74. Lacrimal, posterior process, medial surface for articulation with frontal, inflated by pneumatic recess:
    (0) – no
    (1) – yes
    (Brusatte et al 2010:62)

75. Lacrimal, lacrimal vacuity opening laterally at the angle of the lacrimal above antorbital fenestra:
    (0) – absent
    (1) – present, large, where the vacuity extends posteriorly to the ventral process at least ½ width of ventral process
    (2) – tiny, much less than ½ width ventral process where the vacuity ends cranial to the ventral process
    (Modified from Li et al., 2009:38)
76. Lacrimal, lacrimal vacuity, height to length ratio:
    (0) – tall, greater than 1.1
    (1) – short, less than 0.9
    (2) – subequal
    (Modified from Brusatte et al., 2009:55)
77. Lacrimal, lacrimal vacuity, rim of lacrimal vacuity flush with rim of the antorbital fossa:
    (0) – flush with ventral surface
    (1) – rimmed
    (Loewen et al., 2013:77)
78. Lacrimal, lacrimal vacuity, posterodorsal margin of lacrimal vacuity:
    (0) – rimmed
    (1) – rounded
    (Loewen et al., 2013:78)
79. Lacrimal, orbitonasal ridge on medial surface, position:
    (0) – anterior to posterior margin of ventral process
    (1) – adjacent to or contacting posterior margin of ventral process
    (Carr and Williamson 2010: 69; Brusatte et al 2010:60)
80. Lacrimal, extent of antorbital fossa on ventral process:
    (0) – covers greater than 60% of anteroposterior length along the contact with the jugal
    (1) – less than 60% of anteroposterior length along the contact with the jugal
    (Brusatte et al 2010:63)
81. Lacrimal, subocular process restricting anterior orbit margin on caudolateral margin of ventral process, and defining ocular region of orbit:
    (0) – absent
    (1) – present
    (Modified from Sampson et al., 1998; Currie and Carpenter, 2002:31; Benson, 2009:43; Brusatte et al., 2009:63)

Prefrontal

82. Prefrontal:
    (0) – present
    (1) – absent
    (Li et al., 2009)
83. Prefrontal, exposure on anterior rim of orbit in lateral view:
    (0) – present, with ventral process forming part of the anterior rim of the orbit
    (1) – absent, displaced posteriorly and/or medially with the ventral process absent
    (Modified from Rauhut, 2003; Smith et al., 2007: 61; Sereno et al., 2009:20; Brusatte et al., 2010:111)
84. Prefrontal, extent of exposure on orbital rim in lateral view:
    (0) – exposed with ventral process less than 20% of the diameter of the orbit
    (1) – exposed with long ventral process that extends more than halfway down the ventral process of the lacrimal, anteriorposterior length similar to that of the anterior process of the lacrimal
    (Modified from Carr and Williamson 2010:132; Brusatte et al 2010:112)
85. Prefrontal, shape of prefrontal excluded from the anterior rim of the orbit:
    (0) –ventral process absent, but dorsal exposure similar to that of lacrimal
    (1) – excluded from the anterior rim of the, being displaced posteriorly and/or medially; ventral process absent, and greatly reduced in size
    (Modified from Rauhut, 2003; Smith et al., 2007: 61; Sereno et al., 2009:20; Brusatte et al., 2010:111)

86. Anterior extent of the prefrontal position relative to lateral part of frontal process of nasal:
    (0) – anterior to frontal process of nasal
    (1) – even with or lateral to frontal process of nasal
    (2) – posterior to frontal process of nasal
    (Modified from Brusatte et al., 2009:117)
87. Prefrontal, contact with lacrimal and frontal:
    (0) – prefrontal separates lacrimal and frontal
    (1) – lacrimal and frontal contact each other behind the prefrontal
    (2) – lacrimal and frontal contact each other in front of the prefrontal
    (Modified from Brusatte et al., 2009:119)

Frontal

88. Frontal, anterior margin:
    (0) – narrow anteriorly as an elongate wedge between nasals
    (1) – anterior end terminates abruptly even if w shaped, suture with nasal transversely oriented
    (Modified from Xu et al., 2006; Li et al., 2009:41; Sereno et al. 2009:38; Brusatte et al., 2010:113)
89. Frontal, ratio of anteroposterior length of exposed portion on skull roof to mediolateral width at midpoint of single element:
    (0) – long, greater than 2.5
    (1) – short, less than 2.0
    (Brusatte et al., 2010:114)
90. Frontal, exposure along orbital margin:
    (0) – broadly exposed along orbital margin
    (1) – present but limited to a small notch
    (2) – excluded by postorbital-lacrimal contact in large specimens
    (Li et al., 2009:271; Sereno et al., 2009:21; Carr and Williamson 2010:104; Brusatte et al., 2010:120)
91. Frontal, shape of the prefrontal articular surface on dorsal surface of skull:
    (0) – straight or anteromedial arc
    (1) – notch
    (Modified from Brusatte et al., 2009:129)
92. Frontal, expansion of dorsotemporal fossa onto dorsal surface:
    (0) – dorsotemporal fossa extends anteriorly onto dorsal surface of frontal
    (1) – dorsotemporal fenestra excluded from frontal
    (Modified from Currie 1995; Currie and Varricchio, 2004:14; Li et al., 245)
93. Frontal, dorsotemporal fossa, anterior emargination:
    (0) – straight or curved
    (1) – sinusoidal
    (Currie, 1995; Li et al., 2009:42)
94. Frontal, dorsotemporal fossa, anteroposterior length compared to overall length of exposed portion of frontal on skull roof:
    (0) – less than 30%
    (1) – between 30-45%
    (2) – between 45-55%
    (3) – greater than 60%
    ORDERED
    (Brusatte et al., 2009:123; Carr and Williamson 2010:136-137; Brusatte et al., 2010: 115)
95. Frontal, dorsotemporal fossa, medial extension:
    (0) – fossa restricted to posterolateral corner of frontal
    (1) – meets opposing fossa at the midline
    (Sereno et al., 2009:39; Brusatte et al., 2010:116)

96. Frontal, postorbital suture:
    (0) – dorsoventrally shallow and undifferentiated
    (1) – dorsoventrally shallow (approximately 6 times longer than deep) and differentiated into a vertical region anteriorly and a horizontal region posteriorly
    (2) – dorsoventrally deep (approximately twice as long as deep) and subtly differentiated into vertical and horizontal regions
    ORDERED
    (Sereno et al. 2009:37; Carr and Williamson 2010:145; Brusatte et al., 2010:119)
97. Frontal, shape of frontal viewed between dorsotemporal fossa:
    (0) – flat
    (1) – low flat-topped or low bilateral sagittal crests without midline peak
    (2) – short, low pyramidal sharp sagittal crest
    (3) – long, tall, bladelike sharp sagittal crest on frontal, sometimes flat-topped
    (4) – domed with dorsally convex surface
    (Modified from Brusatte et al., 2009:131; Carr and Williamson 2010:139; Carr and Williamson 2010:141; Brusatte et al., 2010:118; Brusatte et al., 2010:117)

Parietal

98. Parietal-frontal suture, form:
    (0) – transversely smooth
    (1) – tab-like wedge from parietal extends anteriorly to overlie frontal on midline
    (Carr and Williamson 2010:147; Brusatte et al., 2010:121)
99. Parietals, dorsal surface in transverse view:
    (0) – flat
    (1) – comprised of two parallel crests forming flat table
    (2) – transversly convex with single midsagittal crest
    (Modified from Xu et al., 2006; Li et al., 2009:45; Carr and Williamson 2010:150; Brusatte et al., 2009:122,123)
100. Parietals, distinct frontoparietal peak:
    (0) – absent
    (1) – low frontoparietal peak
    (2) – frontoparietal peak ½ to less than 2/3 height of nuchal crest
    (3) – frontoparietal peak nearly as tall as nuchal crest
    ORDERED
    (Loewen et al., 2013:100)
101. Parietals, dorsal surface in lateral view:
    (0) – generally flat or concave
    (1) – arched, dorsally convex
    (Loewen et al., 2013:101)
102. Parietals, shape of flat or concave dorsal surface in lateral view:
    (0) – generally flat
    (1) – shallowly concave
    (2) – deeply concave with depth of concavity approaching ½ of length
    ORDERED
    (Loewen et al., 2013:102)
103. Parietals, nuchal crest height:
    (0) – as low subequal to roof of the skull
    (1) – equal or shorter than tallest part of the skull
    (2) – taller than any other part of the skull
    (Modified from Holtz, 2001; Brusatte et al., 2009:134; Sereno et al., 2009:41; Carr and Williamson 2010:149)

Postorbital

104. Postorbital, anterior (frontal) process length:
    (0) – short, anterior process is approximately half the length of the ventral process
    (1) – long, anterior process is greater than 60% of the length of the ventral process
    (Li et al. 2010:252; Brusatte et al., 2010: 88)
105. Postorbital, anterior process:
    (0) – slender process, thickness is much less than the thickness of the midpoint of the ventral process
    (1) – robust process, thickness is subequal to that of the midpoint of the ventral process
    (Loewen et al., 2013:105)
106. Postorbital, anterior process curvature in lateral view:
    (0) – straight anterior (frontal) process
    (1) – anterior process strongly arched anterodorsally
    (Modified from Li et al., 2009:4)
107. Postorbital, descending process:
    (0) – relatively narrow and comparable to preorbital bar (lacrimal) in anterior/posterior width
    (1) – at least the midpoint of the postorbital bar (formed by descending process of postorbital and sometimes ascending process of jugal) wide and plate-like, about twice as wide as preorbital bar
    (Li et al. 2010: 268)
108. Postorbital, subocular process:
    (0) – absent
    (1) – present, slight expansion into the orbit
    (2) – greatly expanded into the orbit so as to greatly restrict ventral part of orbit
    ORDERED
    (Modified from Sereno et al. 2009:30; Brusatte et al., 2009: 92; Brusatte et al., 2010: 86)
109. Postorbital, subocular process, position:
    (0) – at ventral end of ventral process
    (1) – flange-like, separated from ventral tip of ventral process by a notch
    (Carr and Williamson, 2010:102; Brusatte et al., 2010:87)
110. Postorbital, ornamentation:
    (0) – absent
    (1) – present low sculpturing
    (2) – present cornual boss of rugose grooves
    (3) – present as round knobby cornual boss
    ORDERED
    (Loewen et al., 2013:110)
111. Postorbital, cornual process, position relative to posterior margin of orbit:
    (0) – cornural process positioned posterior to orbit margin
    (1) – cornual process confluent with posterior margin of orbit
    (Loewen et al., 2013:111)
112. Cornual process undercut at base, so that process is differentiated from the rest of the lateral surface:
    (0) – absent
    (1) – present
    (Modified from Brusatte et al., 2009:88)
113. Postorbital, squamosal process, form of posterodorsal margin:
    (0) – uninterrupted convex arc
    (1) – emarginated by squamosal (discrete concave notch within the margin in lateral view)
    (Brusatte et al 2009: 90; Carr and Williamson 2010:99; Brusatte et al 2010:84)
114. Postorbital, squamosal process length:
    (0) – reaches posterior margin of the laterotemporal fenestra
    (1) – stops short of posterior margin of fenestra
    (Brusatte et al 2009: 9; Sereno et al. 2009:28; Carr and Williamson 2010:100; Brusatte et al 2010: 85)
115. Postorbital, expansion of dorsotemporal fossa onto dorsal surface of postorbital:
    (0) – no dorsotemporal fenestra on postorbital
    (1) – covers most of frontal process of the postorbital and extends anteriorly onto dorsal surface of frontal
    (Modified from Currie 1995; Currie and Varricchio, 2004:14; Li et al., 2009:245)

116. Postorbital, postorbito-lacrimal osteoderm:
  (0) – absent
  (1) – present
  (Brusatte et al., 2009:94)

Squamosal

117. Squamosal, anterior extent:
  (0) – at or ahead of anterior margin of the fenestra
  (1) – posterior to anterior margin of laterotemporal fenestra
  (Modified from Brusatte et al., 2009:89)
118. Squamosal, presence of dorsotemporal fossa:
  (0) – absent, fenestra bounded laterally and posteriorly by the squamosal
  (1) – present, fossa extends onto dorsal surface of the squamosal
  (Li et al., 2009:219)
119. Squamosal, web of bone overhangs the posterior border of the dorsotemporal fossa:
  (0) – absent
  (1) – present
  (Modified from Brusatte et al., 2009:95)
120. Squamosal, orientation of web of bone that overhangs dorsotemporal fossa:
  (0) – dorsal
  (1) – lateral
  (Loewen et al., 2013:120)
121. Squamosal, dorsotemporal fossa, surface morphology:
  (0) – flat or concave
  (1) – convex
  (Carr and Williamson 2010:107; Brusatte et al., 2010: 91)
122. Squamosal, descending process orientation:
  (0) – parallels quadrate shaft
  (1) – oblique to quadrate shaft oriented at about 45 degrees
  (2) – nearly perpendicular to quadrate shaft
  (Modified from Li et al., 2009:47; Brusatte et al., 2009: 98; Carr and Williamson 2010:111; Brusatte et al., 2010: 92)
123. Squamosal, non-vertical quadratojugal process, extent of constriction of the laterotemporal fenestra:
  (0) – process constricts less than 80%
  (1) – process constricts more than 90%
  (Loewen et al., 2013:123)
124. Squamosal, quadratojugal process, morphology of anterior tip of entire process in those taxa with horiziontal processes:
  (0) – tapered point
  (1) – squared off
  (Modified from Carr and Williamson 2010:110; Brusatte et al., 2010: 93)
125. Squamosal, horizontal quadratojugal process, relationship of the articulation with quadratojugal:
  (0) – horizontal process with rod-like ridge above quadratojugal and forming lap joint medial to the quadratojugal
  (1) – process forms a broad lap joint with quadratojugal, but visible portion of horizontal process above quadratojugal is tab-like
  (Modified from Brusatte et al., 2009: 99; Brusatte et al., 2010: 94)
126. Squamosal, pneumaticity in the ventral surface of the dorsal portion of bone:
  (0) – absent
  (1) – foramen present
  (2) – foramen and fossa present
  (Modified from Brusatte et al., 2009:103; Loewen et al. 126 now run as unordered)

127. Squamosal, fossa in ventral surface of the dorsal ceiling of bone, shape:
    (0) – broad with rounded margin
    (1) – rimmed with sharp margins
    (Loewen et al., 2013:127)
128. Squamosal, postquadratic process thickness in lateral view measured perpendicular to long axis of the process in the dorsoventral or cranioposterior plane in lateral view:
    (0) – absent or thin
    (1) – subequal but not thin
    (2) – thicker than long
    (Loewen et al., 2013:128)
129. Squamosal, postquadratic process:
    (0) – short
    (1) – at least as long as quadrate cotylus
    (2) – twice as long as quadrate cotylus
    (3) – three or more times as long as the quadrate cotylus
    (Modified from Brusatte et al., 2009:105)

Jugal

130. Jugal, contribution to antorbital fenestra:
    (0) – absent
    (1) – participates along a small surface in the antorbital fenestra
    (2) – extensive, forms the caudoventral corner of antorbital fenestra
    (Modified from Li et al., 2009:246)
131. Jugal, pneumatic recess:
    (0) – absent
    (1) – small and round
    (2) – large and elongate
    (Modified from Sereno et al., 1996; Smith et al., 2007:47)
132. Jugal, pneumatic recess orientation of long axis:
    (0) – axis of recess is horizontal
    (1) – axis of foramen inclined at an angle of 45 degrees to the ventral skull margin
    (Modified from Currie et al., 2003: Li et al., 2009:33)
133. Jugal, position of pneumatic recess in antorbital fossa:
    (0) – antorbital fossa edge does not extend past the pneumatic recess
    (1) – antorbital fossa edge is continues caudoventral to the pneumatic recess
    (Modified from Brusatte et al., 2009: 68)
134. Jugal, maxillary process, antorbital fossa position relative to ventral process of lacrimal:
    (0) – posterior edge of fossa posterior to or directly ventral to midpoint of ventral process
    (1) – posterior edge of fossa anterior to midpoint of ventral process
    (Modified from Brusatte et al., 2009:69)
135. Jugal, maxillary process, dorsoventral depth:
    (0) – shallow, not expanded relative to suborbital portion of bone
    (1) – deep, expanded relative to suborbital portion of bone
    (Carr and Williamson 2010: 73; Brusatte et al., 2010: 65)
136. Jugal, suture with lacrimal, angle of the posterior half of the contact:
    (0) – low, suture sub-horizontal
    (1) – steep with posteroventral tab
    (Modified from Brusatte et al., 2009: 74; Carr and Williamson 2010:81; Brusatte et al., 2010: 70)
137. Jugal, suboccular process on ventral part of orbit to accept the suboccular flange of the postorbital:
    (0) – absent, postorbital process confluent with suborbital margin
    (1) – present, slight notch between postorbital articulation and suborbital margin
    (2) – present, pronounced flange corresponding to suboccular flange of postorbital
    ORDERED
    (Loewen et al., 2013:137)

138. Jugal, postorbital process width at base:
    (0) – narrow less than 1/4 length of jugal
    (1) – wide, greater than 1/3 length of jugal
    (Loewen et al., 2013:138)
139. Jugal, postorbital facet of ascending process:
    (0) – absent or shallow
    (1) – mediolaterally oriented scarf joint
    (Modified from Brusatte et al., 2009:75)
140. Jugal, postorbital process, orientation relative to ventral margin of jugal in life position:
    (0) – approximately perpendicular
    (1) – posteriorly inclined 15-25 degrees from perpendicular
    (2) – strongly posteriorly inclined to 30 or more degrees
    (Modified from Brusatte et al., 2010: 75)
141. Jugal, shape of orbital margin:
    (0) – weakly concave, approximately level with lacrimal-jugal suture
    (1) – U-shaped, extends ventral to lacrimal-jugal suture
    (Sereno et al., 2009: 26; Brusatte et al., 2010: 79)
142. Jugal, ventral margin:
    (0) – ventral margin relatively straight or gently convex
    (1) – ventral margin convex rugose peak
    (Loewen et al., 2013:142)
143. Jugal, lateral jugal ornamentation:
    (0) – absent
    (1) – rugose thickening present
    (Loewen et al., 2013:143)
144. Jugal, dorsal prong of quadratojugal ramus, slope in lateral view:
    (0) – horizontal
    (1) – posterodorsal
    (Brusatte et al., 2009: 82; Carr and Williamson 2010:91; Brusatte et al., 2010: 77)
145. Jugal, raised rim on the ventrolateral surface, paralleling the ventral margin of the bone and anteriorly confluent with the antorbital fossa rim of the maxilla:
    (0) – absent
    (1) – present
    (Modified from Brusatte et al., 2010:80)

Quadratojugal

146. Quadratojugal, form of jugal articulation:
    (0) – dorsal prong of posterior process of jugal does not approach the base of the quadratojugal
    (1) – does approach the base of the quadratojugal (the corner where the anterior and dorsal processes of the quadratojugal meet)
    (Carr and Williamson 2010:120; Brusatte et al 2010: 100)
147. Quadratojugal, constriction of lateral temporal fenestra:
    (0) – absent, dorsal process is approximately vertical
    (1) – present, convex kink along the suture between the quadratojugal and squamosal that projects into the fenestra, constricting it to approximately one half of its maximum anteroposterior length
    (2) – present, dorsal region of quadratojugal moderately expanded anteroposteriorly relative to the remainder of the bone, constricting fenestra to approximately one half of its maximum anteroposterior length
    (3) – present, dorsal region of quadratojugal expanded anteroposteriorly by at least twice the minimum anteroposterior dimension of the bone, forming a flange that meets the ventral process of the squamosal to nearly divide the fenestra
    ORDERED
    (Modified from Sereno et al. 2009:35; Carr and Williamson 2010:116; Brusatte et al., 2010: 98)

148. Quadratojugal, anterior process, dorsoventrally expanded so majority of process overlaps laterally onto jugal:
(0) – absent
(1) – present
(Loewen et al., 2013:148)

149. Quadratojugal, overall form of jugal process:
(0) – elongate tapered process
(1) – tab-like process with rounded anterior end
(2) – tab-like process with squared-off anterior end
ORDERED
(Modified from Currie et al., 2003; Sereno et al., 2009:34; Carr and Williamson 2010:121; Brusatte et al., 2010: 101)

150. Quadratojugal, squamosal-quadratojugal contact:
(0) – at tips or absent
(1) – broad
(Carrano et al., 2002, 2005; Rauhut, 2003; Smith et al., 2007:82)

151. Quadratojugal, anteroposterior breadth of quadratojugal dorsal process:
(0) – narrow
(1) – broad
(Smith et al., 2007:83)

152. Quadratojugal, dorsal process, ridge along anterior margin of lateral surface:
(0) – absent
(1) – present, subtle and fades in strength dorsally
(2) – present, robust and extends to the dorsal margin of the bone
(Brusatte et al., 2009: 107; Carr and Williamson 2010:117; Brusatte et al., 2010: 99; Loewen et al. 2013:152 now run as unordered)

153. Quadratojugal, presence of posterior process:
(0) – absent, without horizontal process posterior to ascending process (reversed "L" shape)
(1) – present, process inverted 'T' or 'Y' shape
(Modified from Li et al., 2009:35, 281; Brusatte et al 2010: 103)

154. Quadratojugal, orientation of posterior process:
(0) – lateral facing process
(1) –posterior facing process
(Modified from Li et al., 2009:35, 281; Brusatte et al 2010: 103)

Quadrate

155. Quadrate foramen size:
(0) – absent
(1) – small less than 3% element dorsoventral height
(2) – more than 5% element height but less than 40% element height
(3) – more than 45% element height
(Modified from Holtz, 2004; Li et al., 2009:275; Loewen et al. 2013 now run as unordered)

156. Quadrate foramen conformation:
(0) – almost completely formed within the quadrate
(1) – 75% of foramen formed by the quadrate
(Modified after Rauhut, 2003)

157. Quadrate foramen orientation:
(0) – posteriorly, not visible in lateral view
(1) – caudolaterally, obliquely visible in lateral view
(2) – laterally, completely visible in lateral view
(Loewen et al., 2013:157)

158. Quadrate foramen position:
(0) – at midheight or dorsal to midheight
(1) – ventral, near the quadrate neck
(Loewen et al., 2013:158)

159. Quadrate, pneumaticity:
   (0) – absent
   (1) – present, deep recess on the anterior surface where the pterygoid wing and condyles meet
   (Molnar, 1985; Currie et al., 2003; Carr and Williamosn 2009:126; Brusatte et al 2010: 114: Brusatte et al 2010: 106)
160. Quadrate, oval fossa on medial surface of pterygoid wing:
   (0) – absent
   (1) – present shallow fossa
   (2) – present deep fossa
   (Loewen et al., 2013:160; now run as unordered)
161. Quadrate condyles, position relative to occipital condyle when skull is in articulation:
   (0) – anterior to or approximately aligned
   (1) – completely posterior
   (Carr and Williamson 2010:127; Sereno et al. 2009:42; Brusatte et al 2010: 107)

Supraoccipital

162. Supraoccipital, form of dorsal margin:
   (0) – smoothly convex and undivided
   (1) – divided into two processes ("bilobate")
   (Brusatte et al., 2009:158; Carr and Williamson 2010:172; Brusatte et al., 2010: 149)
163. Supraoccipital, distinct dorsal process of supraoccipital:
   (0) – absent
   (1) – present
   (Brusatte et al., 2009: 155)
164. Supraoccipital, width of dorsal process of supraoccipital:
   (0) – narrow
   (1) – wide
   (Brusatte et al., 2009: 156)
165. Supraoccipital, dorsal process of supraoccipital form of dorsal surface:
   (0) – flat or peaked
   (1) – forked
   (Loewen et al., 2013:166)
166. Supraoccipital, form of median posterior surface of dorsal process:
   (0) – flat
   (1) – midline peaked ridge
   (2) – midline groove present
   (Loewen et al., 2013:166)
167. Supraoccipital, median ridge above dorsal process:
   (0) – absent or no differentiation between dorsal process and median ridge
   (1) – present; slight, peaked ridge
   (2) – present; well-developed, wide ridge
   ORDERED
   (Modified from Holtz, 2001; Currie et al., 2003; Brusatte et al., 2009:157)
168. Supraoccipital, contribution to dorsal rim of foramen magnum:
   (0) – forms entire rim
   (1) – makes limited contribution to rim via triangular ventral process
   (2) – completely excluded from rim
   (Harris, 1998; Currie et al., 2003; Brusatte et al., 2009:158; Carr and Williamson 2010:158; Brusatte et al., 2010:148)

Prootic

169. Prootic, depression for pneumatic recess:
   (0) – absent
   (1) – present as dorsally open fossa on prootic/opisthotic
   (Li et al., 2009; Brusatte et al., 2010: 163)

Opisthotic

170. Opisthotic, posterior (caudal) tympanic recess:
   (0) – absent
   (1) – present
   (Li et al., 2009:18)
171. Opisthotic, posterior (caudal) tympanic recess position:
   (0) – posterior
   (1) – anterior
   (Modified from Brusatte et al., 2009:141)
172. Opisthotic, ventral extension:
   (0) – notch separates basal tuber from more anteroventral extension of exoccipital-basisphenoid suture
   (1) – notch absent (Currie et al., 2003; Brusatte et al., 2009:142)
173. Opisthotic, orientation of the paroccipital processes:
   (0) – directed laterally, or slightly ventrolaterally
   (1) – directed strongly ventrolaterally,
   (Modified from Rauhut, 1997; Rauhut, 2003; Smith et al., 2007:90; Li et al., 2009)
174. Opisthotic, ventral extent of the paroccipital processes:
   (0) – above or level with the dorsal border of the occipital condyle
   (1) – situated at mid-height of occipital condyle or lower
   (Rauhut, 2003; Smith et al., 2007:91)
175. Opisthotic, curvature of the ventral surface paroccipital process:
   (0) – straight
   (1) – curving ventrally, pendant
   (Currie et al., 2003; Brusatte et al., 2009:144)
176. Opisthotic, paroccipital process length:
   (0) – elongate and slender
   (1) – process short, thick, 60% or more of dorsoventral height compared to mediolateral length
   (Modified from Smith et al., 2007; Li et al., 2009:56)
177. Opisthotic, exoccipital-opisthotic, paroccipital processes, deep fossa on posterior surface dorsolateral to the foramen magnum:
   (0) – absent
   (1) – present and shallow
   (2) – present and deep
   (Modified from Brusatte et al., 2010: 151; Loewen et al., 177 now run as unordered)
178. Opisthotic, exits of cranial nerves X-XII:
   (0) – flush with surface of exoccipital
   (1) – cranial nerve exits located together in a bowl-like depression
   (Li et al., 2009:19; Brusatte et al., 2010:153)
179. Opisthotic, exoccipital-opisthotic, crista tuberalis (= metotic strut), extent in posterior view:
   (0) – crista tuberalis absent or extent very limited
   (1) – extensive, extends subequal to the length of the paroccipital process
   (Modified from Brusatte et al., 2010:152)

Laterosphenoid

180. Laterosphenoid, transverse scar on lateral surface:
   (0) – absent
   (1) – present
   (Modified from Brusatte et al., 2009:137)
181. Laterosphenoid, dorsal suture with the parietal:
   (0) – flat
   (1) – raised into a sharp ridge laterally
   (Brusatte et al., 2009:138)

182. Laterosphenoid, antotic crest separating lateral wall of braincase from orbital and temporal spaces:
(0) – absent or indistinct
(1) – present; robust and rugose
(Carr and Williamson 2010:152, 155; Brusatte et al., 2010:164)
183. Laterosphenoid, antotic crest, form:
(0) – single structure
(1) – bifurcates ventrally
(Brusatte et al., 2010:165)
184. Laterosphenoid, fossa on lateral surface that houses head of epipterygoid:
(0) – absent or shallow
(1) – present, deep and rugose
(Brusatte et al., 2010:166)
185. Laterosphenoid, ventrolateral shelf:
(0) – absent
(1) – present
(Modified from Brusatte et al., 2009:140)

Basioccipital

186. Basioccipital, orientation of occipital surface:
(0) – faces directly posteriorly
(1) – faces posteroventrally
(Holtz, 2001; Currie et al., 2003; Brusatte et al., 2009:3; Sereno et al. 2009:46; Brusatte et al., 2010:147)
187. Basioccipital, occipital condyle constriction of neck:
(0) – lateral constriction of neck
(1) – subspherical without constricted neck
(Li et al., 2009:55)
188. Basioccipital, subcondylar recesses:
(0) – absent
(1) – shallow
(2) – deep
ORDERED
(Holtz, 2004; Li et al. 2009:255; Carr and Williamson 2010:168; Brusatte et al., 2010:156)
189. Basioccipital, basicranium in ventral view:
(0) – longer than wide, or with subequal proportions
(1) – clearly wider than long
(Currie et al., 2003; Li et al. 2009:272; Sereno et al. 2009:49; Carr and Williamson 2010:169; Brusatte et al., 2010:161)
190. Basioccipital, basal tubera:
(0) – equally formed by basioccipital and basisphenoid and not subdivided
(1) – subdivided by a lateral longitudinal groove into a medial part entirely formed by the basioccipital, and a lateral part, entirely formed by the basisphenoid
(Rauhut, 2003; Smith et al., 2007:100)
191. Basioccipital, basal tubera mediolateral width:
(0) – greater than occipital condyle width
(1) – equal to or less than occipital condyle width
(Currie et al., 2003; Makovicky et al., 2003; Smith et al., 2007:102)
192. Basioccipital, basal tubera, dorsoventral depth:
(0) – less than depth of occipital condyle
(1) –greater than depth of occipital condyle
(Brusatte et al., 2010:154)
193. Basioccipital, basicranium,mediolateral width of basal tubera versus the width of the basispterygoid processes:
(0) – subequal or basal tuber wider
(1) – basipterygoids wider
(Loewen et al., 2013:193)

194. Basioccipital, basal tubera in posterior view, concave notch ventrally between opposing tubera, dorsoventral depth:
  (0) – shallow, less than 30% depth of tubera
  (1) – deep, more than 30% depth of tubera
  (Brusatte et al., 2010:155)
195. Basioccipital, ventral surface across basal tubera and basisphenoids orientation:
  (0) – oriented nearly horizontally
  (1) – oriented caudoventrally
  (Harris, 1998; Currie et al., 2003:149; Brusatte et al., 20090:149)
196. Basioccipital, pneumatic openings associated with internal carotid artery:
  (0) – absent
  (1) – present
  (Allain, 2002; Coria and Currie, 2006; Smith et al., 2007:106; Brusatte et al., 20090:146)

Parabasisphenoid
197. Parabasisphenoid, with large surfaces of pronounced muscle scars flanking basisphenoid recess:
  (0) – no
  (1) – yes
  (Bakker et al. 1998; Li et al., 2009:280)
198. Parabasisphenoid, basipterygoid recess (pneumatic recess above basipterygoid processes on lateral surface of braincase):
  (0) – absent or very shallow pneumatic fossa
  (1) – present as a deep fossa
  (Carr and Williamson 2010:160; Brusatte et al., 2010:157)
199. Parabasisphenoid, basisphenoid recess:
  (0) – undivided
  (1) – divided
  (Modified from Li et al., 2009:10)
200. Parabasisphenoid, orientation of division of basisphenoid recess:
  (0) – transversely divided so that there is an anterior and posterior recess
  (1) – divided by a single midline strut into two laterally separate chambers
  (2) – divided by a "Y" shaped strut of bone forming three recesses
  (Modified from Li et al., 2009:10; Loewen et al., 2013 now run as unordered)
201. Parabasisphenoid, basisphenoid recess, inflation of the dorsal surface of the recess:
  (0) – absent
  (1) – present
  (Carr and Williamson 2010:166; Brusatte et al., 2010:159)
202. Parabasisphenoid, basisphenoid recess, orientation of central axis:
  (0) – horizontal, recess obscured in posterior view
  (1) – posteroventral, recess partially visible in posterior view
  (2) – extremely posteroventral, recess compressed anteroposteriorly and widely visible in posterior view, and basipterygoid processes located beneath the basal tubera
  ORDERED
  (Sereno et al. 2009:50; Carr and Williamson 2010:163,165; Brusatte et al., 2010: 158)
203. Parabasisphenoid, shape of rostrum:
  (0) – anteroposteriorly expanded, ventral margin is a smooth concave arch
  (1) – dorsoventrally expanded, ventral margin is nearly vertical posteriorly and then abruptly transitions to horizontal trend anteriorly
  (Brusatte et al., 2010:162)

Palate
204. Palate, shelf-like primary palate, presence:
  (0) – absent
  (1) – present
  (Currie et al., 2003; Brusatte et al., 2009:159)
205. Palate, internal choana, shape:

(0) – anteroposteriorly elongate oval
(1) – nearly circular
(Brusatte et al., 2010:145)

206. Palate, suborbital fenestra, shape:
(0) – anteroposteriorly elongate oval
(1) – nearly circular
(Brusatte et al., 2010:146)

Vomer

207. Vomer, anterior horizontal expansion:
(0) – absent
(1) – present, slight expansion
(2) – present, expanded to equilateral diamond shape
ORDERED
(Modified from Carr, 1999; Sereno et al. 2009:45; Bursatte et al., 2009:160; Carr and Williamson 2010:176; Brusatte et al., 2010: 128)

Palatine

208. Palatine anterior expansion of dorsal process into a vomerine process:
(0) – absent
(1) – present
(Loewen et al., 2013:209)

209. Palatine, vomeropterygoid process, anteroposterior length of dorsal margin ratio to length of greatest constriction of process neck:
(0) – long, greater than 2.0
(1) – short, or less than 2.0
(Carr and Williamson 2010:188; Brusatte et al., 2010:134)

210. Palatine posterior expansion of dorsal process:
(0) – absent
(1) – present
(Loewen et al., 2013:210)

211. Palatine, overall orientation of dorsal surface of vomerine process:
(0) – rostrodorsally directed at 45 degrees
(1) – horizontal
(Modified from Brusatte et al., 2009:173; Carr and Williamson 2010:189; Brusatte et al., 2010:135)

212. Palatine, dorsoventral height of the dorsal process:
(0) – tall, 60% or more than narrowest anteroposterior width of the neck of the dorsal process
(1) – short, less than 50%
(Modified from Brusatte et al., 2009:171)

213. Palatine, overall orientation of neck of dorsal process:
(0) – rostrodorsally directed at nearly 45 degrees
(1) – vertically directed
(Loewen et al., 2013:213)

214. Palatine, posterior expansion of base of the neck of the dorsal process:
(0) – absent
(1) – present
(Loewen et al., 2013:214)

215. Palatine, pneumatic recess, number of external pneumatic openings:
(0) – absent
(1) – present, one opening
(2) – present, two openings
(Modified from Brusatte et al., 2009:175; Carr and Williamson 2010: 190; Brusatte et al., 2010:136, 137)

216. Palatine, anterior or primary opening of palatine recess, location of posterior margin:
(0) – posterior to or even with the posterior margin of the neck of the vomeropterygoid process
(1) – anterior to posterior margin of the vomeropterygoid process neck
(Carr and Williamson 2010:194; Brusatte et al., 2010:138)
217. Palatine, position of the posterior edge of the posterior pneumatic recess compared to the posterior edge of the dorsal process:
(0) – anterior to the posterior margin of dorsal process
(1) – extends posterior to the posterior margin of the process
(Brusatte et al., 2009:177)
218. Palatine, presence of slot on the articular surface for the maxilla:
(0) – absent
(1) – present
(Brusatte et al., 2009:180)
219. Palatine, maxillary process, form of maxillary articulation:
(0) – flat
(1) – deeply excavated as a slot, demarcated dorsally by a pronounced lip of bone
(Carr and Williamson 2010:197; Brusatte et al., 2010: 141)
220. Palatine, maxillary articulation, form:
(0) – maxilla abuts lateral surface of maxillary process and anterior region of jugal process
(1) – contact reinforced by a “brace” at the anteroventral corner of the jugal process, which sits within internal antorbital fossa
(Brusatte et al., 2010:143)
221. Palatine, morphology of maxillary articulation brace:
(0) – projects ventrally due to a jugal process that extends further ventrally than the maxillary process, such that there is a discrete corner between the two processes in lateral view
(1) – projects laterally, with no discrete corner between the smoothly confluent jugal and maxillary processes in lateral view
(Brusatte et al., 2010:144)
222. Palatine, jugal process, location of contact surface for lacrimal:
(0) – posterior (“distal”), separated from opening of palatine recess by wide margin
(1) – anterior (“proximal”), closely approaches opening of palatine recess
(Carr and Williamson 2010:196; Brusatte et al., 2010:140)
223. Palatine, extension of pneumatic recess into jugal process:
(0) – no extension
(1) – yes, process visibly inflated
(Carr and Williamson 209:199; Brusatte et al., 2010:142)

Epipterygoid

224. Epipterygoid ventral margin:
(0) – undivided
(1) – forked
(Brusatte et al., 2009:161)

Ectopterygoid

225. Ectopterygoid, body pneumatic:
(0) – apneumatic
(1) – pneumatic, uninflated
(2) – pneumatic with inflated body
(Modified from Brusatte et al., 2009:162; Sereno et al. 2009:44; Carr and Williamson 2010:178; Brusatte et al., 2010:129; Loewen et al., 2013:225 now run as unordered)
226. Ectopterygoid, external opening of pneumatic recess, shape:
(0) – thin ovoid slot
(1) – larger, round or triangular
(Carr and Williamson 2010:183; Brusatte et al., 2010:132)

227. Ectopterygoid, surface posteriorly adjacent to external opening of pneumatic recess, form:
    (0) – flat, recess grades smoothly into the floor of the lateral temporal fenestra (=subtemporal fenestra)
    (1) – lip, recess separated from lateral temporal fenestra (=subtemporal fenestra)
    (Carr and Williamson 2010:185; Brusatte et al., 2010:133)
228. Ectopterygoid, jugal process, external pneumatic foramina leading into jugal recess:
    (0) – absent
    (1) – present
    (Brusatte et al., 2009:164; Carr and Williamson 2010:180; Brusatte et al., 2010:130)
229. Ectopterygoid, jugal process inflation:
    (0) – uninflated
    (1) – inflated by internal pneumaticity
    (Carr and Williamson 2010:181; Brusatte et al., 2010:131)
230. Ectopterygoid, mediolateral length of jugal process:
    (0) – short
    (1) – long
    (Brusatte et al., 2009:163)
231. Ectopterygoid, dorsoventral thickening of jugal process:
    (0) – thin jugal process
    (1) – thickened
    (Brusatte et al., 2009:165)
232. Ectopterygoid, dorsal recess form:
    (0) – absent
    (1) – present forms dorsal lip
    (Li et al., 2009:60)

Pterygoid

233. Pterygoid, contact between pterygoid and palatine:
    (0) – continuous
    (1) – discontinuous in the mid-region, resulting in a subsidiary palatal fenestra
    (Ostrom, 1969; Smith et al., 2007:119)

Mandible

234. Mandible, mandibular ramus, dorsoventral depth of dentary at level of dentary-surangular contact on the dorsal margin of the lower jaw:
    (0) – shallow, less than 20% of the total anteroposterior length of the lower jaw
    (1) – deep, greater than 20% of the total anteroposterior length of the lower jaw
    (Carr and Williamson 2010:200; Brusatte et al 2009: 183; Brusatte et al.,2010: 168)
235. External mandibular fenestra:
    (0) – absent
    (1) – present; tiny
    (2) – present; large
    (Hwang et al., 2004; Peyer, 2007; Li et al., 2009: 253; Sereno et al., 2009:51;Carr and Williamson, 2010:217; Brusatte et al., 2010: 169)
236. Mandible, internal mandibular fenestra size:
    (0) – small and slit-like
    (1) – large and rounded
    (Currie 1995; Li et al., 2009:73)
237. Mandible, madibular joint, position in lateral view:
    (0) – anterior to or even with the paroccipital process
    (1) – posterior to the paroccipital process
    (Brusatte et al., 2009:115)

238. Mandible, lower jaw, articulation, glenoid position relative to level of alveolar margin of dentary with mandible occluded:
    (0) – level or ventral
    (1) – dorsal
    (Sereno et al., 2009:54; Brusatte et al., 2010: 170)
239. Mandible, occlusion in lateral view:
    (0) – jaws occlude for their full length
    (1) – jaws diverge anteriorly due to kink and downward deflection in dentary buccal margin
    (Li et al., 2009:215)

Dentary

240. Dentary, dorsal upturn of element at anterior end:
    (0) – absent
    (1) – present
    (Loewen et al., 2013:240)
241. Dentary shape in lateral view:
    (0) – with subparallel dorsal and ventral edges
    (1) – subtriangular in lateral view, posteriorly expanded
    (Li et al., 2009:70)
242. Dentary, expansion of posterior dentary:
    (0) – posterior height of dentary two times or less than minimum dentary height
    (1) – posterior end of dentary more than two times the minimum dentary height
    (Loewen et al., 2013:242)
243. Dentary, symphysis, texture:
    (0) – generally smooth
    (1) – strongly rugose and beveled, with interlocking ridges and convexities for articulation with the opposing symphysis
    (Brusatte et al., 2010: 173)
244. Dentary, primary neurovascular foramina, arrangement:
    (0) – distinct foramina or set into a shallow groove posteriorly
    (1) – set into a deep and sharp groove across the middle and posterior regions of the dentary
    (Brusatte et al., 2010: 176)
245. Dentary, overall shape of dorsal surface (excluding anterior upturn if present):
    (0) – horizontal, straight
    (1) – dorsally concave
    (Brusatte et al., 2010: 177)
246. Dentary, ventral margin of bone in lateral view, profile:
    (0) – straight
    (1) – sigmoidal
    (Loewen et al., 2013:246)
247. Dentary, posterior end, presence of posterodorsal process above external mandibular fenestra:
    (0) – present
    (1) – absent
    (Modified from Li et al 2009: 68)
248. Dentary, posterior end, presence surangular process on the dorsal surface of the mandible:
    (0) – absent
    (1) – present
    (Modified from Li et al 2009: 68)
249. Dentary, Meckelian groove, dorsoventral depth:
    (0) –wide and shallowly inset into medial surface of bone
    (1) –narrow and deeply inset into bone, groove appears as a thin, sharp structure
    (Brusatte et al., 2010: 178)
250. Dentary, position of Meckelian groove:
    (0) – closer to ventral margin of bone
    (1) – roughly at midheight
    (Modified from Bursatte et al., 2009:195)

251. Dentary, anterior extent of lingual bar:
    (0) – medial to first alveolus
    (1) – medial to second (or further posterior) alveolus (Bursatte et al., 2009:196)
252. Dentary, articular surface for splenial along ventral region of dentary ramus below the Meckelian fossa, form:
    (0) – dorsoventrally shallow and smooth
    (1) – dorsoventrally deep (nearly as deep as anterior depth of fossa) and rugose
    (Brusatte et al., 2010:174)
253. Dentary, angle of the anterior end in lateral view:
    (0) – anterior end relatively vertical with transition point between dorsal and ventral surfaces below alveoli 1-3 when present
    (1) – anterior end angled with transition point between dorsal and ventral surfaces below alveolus 4 or further back
    (Bursatti et al., 2009:197; Carr and Williamson 2010:215; Brusatte et al., 2010: 171)
254. Dentary, anteriormost alveoli, size in comparison to alveoli in middle of tooth row:
    (0) – approximately same size
    (1) – first two alveoli substantially smaller
    (2) – first alveolus substantially smaller
    (Brusatte et al., 2010:175)
255. Dentary, distinct interdental plates:
    (0) – present
    (1) – absent
    (Modified from Li et al 2009: 90)

Coronoid and Supradentary

256. Supradentary ossification, shape:
    (0) – elongate, shallow strip
    (1) – deep, crescentic shape
    (Brusatte et al., 2010:194)
257. Supradentary and coronoid ossifications, form of contact at their zone of fusion:
    (0) – smoothly confluent
    (1) – offset by a concave notch
    (Brusatte et al., 2010:195)

Splenial

258. Splenial, anterior margin:
    (0) – tapered point
    (1) – squared or jagged anterior end
    (Loewen et al., 2013:259)
259. Splenial, form of posterior end:
    (0) – straight or slightly curved
    (1) – distinctly forked
    (Sereno et al., 1996; Smith et al., 2007:131)
260. Splenial, mylohyoid foramen:
    (0) – small, smaller than dentary alveoli
    (1) – large, subequal to or larger than dentery alveoli
    (Modified from Carr and Williamson 2010:210; Brusatte et al., 2010: 191)
261. Splenial, myohyoid foramen orientation of long axis:
    (0) – horizontal
    (1) – dorsoposteriorly inclined in line with intercoronoid process
    (Modified from Carr and Williamson 2010:210; Brusatte et al., 2010: 191)
262. Splenial, mediolateral thickness of intercoronoid process:
    (0) – no thickening
    (1) – thickened mediolaterally
    (Loewen et al., 2013:262)

Angular

263. Angular, exposure in lateral view:
    (0) – exposed almost to end of mandible in lateral view, reaches or almost reaches articular
    (1) – excluded from posterior end
    (Li et al. 2009:263)
264. Angular, exclusion from posterior end:
    (0) – end of angular stops well short of glenoid
    (1) – stops near the anterior end of glenoid
    (Li et al. 2009:264)
265. Angular posterior process:
    (0) – posterior process, point projects dorsally above ventral margin of mandible
    (1) – posterior process tip flush with ventral margin of mandible
    (Li et al. 2009:265)
266. Angular, ventral margin, form:
    (0) – smoothly convex
    (1) – anterior region "flexed" relative to posterior region, such that there is a discrete step between them
    (Brusatte et al., 2010:189)

Surangular

267. Surangular, dorsoventral depth:
    (0) – shallow, element less than 35% craniocaudal length
    (1) – deep, depth 40% or more element anteroposterior length
    (Loewen et al., 2013:267)
268. Surangular, lateral surface, wide depression present for dorsal surangular process of the dentary:
    (0) – absent or narrow groove
    (1) – wide depression
    (Loewen et al., 2013:268)
269. Surangular, groove on dorsolateral surface of anterior end:
    (0) – absent
    (1) – present, shallow groove faintly visible
    (2) – present, deep well-demarked groove
    (Loewen et al., 2013:269; now run as unordered)
270. Surangular, foramen in dorsolateral groove:
    (0) – absent
    (1) – present, small foramen much smaller than groove
    (2) – present, large foramen subequal to groove in diameter
    (Loewen et al., 2013:270; now run as unordered)
271. Surangular, anteroventral extension:
    (0) – extension is absent
    (1) – encloses external mandibular fenestra by contacting the angular anteriorly
    (Currie et al., 2003; Brusatte et al., 2009:190)
272. Surangular, surangular shelf on lateral surface, form:
    (0) – absent or low ridge
    (1) – prominent, shelf present but forms a dorsoventrally thin ridge
    (2) – prominent, well-developed shelf that is dorsoventrally deep
    ORDERED
    (Holtz, 2001 in Currie et al., 2003; Xu et al., 2006; Sereno et al. 2009:53; Brusatte et al. 2009:191; Carr and Williamson 2010:209; Brusatte et al., 2010: 180)
273. Surangular, surangular shelf, orientation relative to the long axis of the lower jaw:
    (0) – ventrally inclined anteriorly or sigmoidal
    (1) – dorsally inclined anteriorly
    (2) – horizontal
    (Modified from Brusatte et al., 2010: 182)

274. Surangular foramen in lateral surface of surangular anterior to mandibular articulation:
(0) – absent
(1) – tiny foramen, smaller or subequal to glenoid fossa
(2) – large surangular foramen present (bigger than glenoid fossa in diameter)
ORDERED
(Modified from Li et al. 2009:74; Sereno et al. 2009:52; Carr and Williamson 2010:204; Brusatte et al., 2010: 179)
275. Surangular, position of surangular shelf in relation to surangular foramen:
(0) – placed far dorsal to posterior surangular foramen
(1) – foramen abuts shelf but shelf projects laterally and does not overhang foramen
(2) – shelf projects ventrolaterally to overhang foramen
(Carr and Williamson 2010:208; Brusatte et al., 2010: 181)
276. Surangular, pneumatic fossa posterodorsal to posterior surangular foramen:
(0) – absent
(1) – present and shallow sometimes with a small foramen inside foramen
(2) – present and deeply invaginated with large foramen
ORDERED
(Brusatte et al., 2010: 183)
277. Surangular, adductor muscle attachment site dorsal to surangular shelf, orientation:
(0) – faces primarily dorsally
(1) – faces almost equally dorsally and laterally
(2) – faces primarily laterally
(Brusatte et al., 2010: 184)
278. Surangular, laterally inclined flange on caudodorsal surface for articulation with lateral process of lateral quadrate condyle:
(0) – absent
(1) – present
(Li et al., 2009:212)

Prearticular

279. Prearticular shape:
(0) – dorsally concave sweeping low curve in lateral view
(1) – subcircular in lateral view, “U” shaped
(Loewen et al., 2013:27)
280. Prearticular, anterior process expansion:
(0) – unexpanded
(1) – expanded into a paddle like shape, more than 220% the dorsoventral depth of the midshaft
(Loewen et al., 2013:280)
281. Prearticular, midshaft cross-sectional shape:
(0) – mediolaterally flattened, plate-like
(1) – sub-round with sub-equal width and height
(2) – round
ORDERED
(Loewen et al., 2013:281)
282. Prearticular, ventral process or flange:
(0) – absent
(1) – present
(Loewen et al., 2013:282)
283. Prearticular, ventral bar, series of tall ridges and deep grooves on lateral surface to strengthen articulation with angular:
(0) – absent
(1) – present
(Brusatte et al., 2010: 193)

284. Prearticular, shape of caudoventral margin:
   (0) – sweeping ventrally convex curve
   (1) – sigmoidal with downturned articular process
   (Loewen et al., 2013:284)

Articular

285. Articular, medial process:
   (0) – absent
   (1) – present
   (Li et al., 2009:77)
286. Articular, retroarticular process shape:
   (0) –rod-like, sort
   (1) – rod-like, elongate and slender
   (2) – short, wide, and with distinct posteriorly facing concavity
   (Li et al., 2009:269)
287. Articular, smooth non-articular region between glenoid and attachment site for depressor mandibular muscles:
   (0) – present
   (1) – absent
   (Brusatte et al., 2010: 190)
288. Articular, pneumatic foramen posterior to glenoid:
   (0) – absent
   (1) – present
   (Currie et al., 2003; Brusatte et al., 2009: 185)

Dentition

289. Premaxilla, number of teeth:
   (0) – 5 or more
   (1) – 4
   (2) – 3
   (3) – edentulous
   (Modified after Li et al., 2009:80)
290. Premaxillary teeth, position of mesial carina:
   (0) – symmetrical
   (1) – offset mesial to distal carina on all teeth (teeth ziphodont)
   (2) – rotated distally on premaxillary teeth 1 and 2 (anterior teeth "D-shaped" and posterior teeth ziphodont)
   (3) – rotated distally on all teeth
   (Modified from Bakker et al., 1988; Harris, 1998:46; Smith et al., 2007:19; Benson, 2009:84; Li et al. 2009:91; Sereno et al. 2009:56-58; Carr and Williamson 2010:219; Brusatte et al., 2010:196)
291. Premaxillary teeth, median vertical ridge on lingual surface:
   (0) – absent
   (1) – present as subtle structure in anterior (mesial) premaxillary teeth
   (2) – present as pronounced structure in all premaxillary teeth
   ORDERED
   (Sereno et al. 2009:60; Brusatte et al., 2010:198)
292. Premaxillary teeth, curvature of posterior (posterior) teeth:
   (0) – recurved
   (1) – straight
   (Brusatte et al., 2010:199)
293. Premaxillary teeth, size compared to rostal-most maxillary tooth:
   (0) – subequal
   (1) – significantly smaller
   (Modified from Li et al., 2009: 257; Sereno et al. 2009:59; Brusatte et al., 2010: 197)

294. Premaxillary teeth, presence of serrations:
 (0) – present
 (1) – absent on at least the mesial carina or completely absent
 (Modified after Holtz, 2004; Li et al., 2009: 256)
295. Maxillary teeth, presence:
 (0) – present
 (1) – absent edentulous
 (Holtz, 2004; Li et al., 2009: 82)
296. Maxilla, position of carina on anteriormost tooth:
 (0) – symmetrical
 (1) – asymmetrical so that tooth is D shaped
 (Loewen et al., 2013:28)
297. Maxilla, position of the posteriormost tooth:
 (0) – nearer to the posterior end of the antorbital fenestra
 (1) – posterior to but closer to the midpoint of the antorbital fenestra
 (2) – anterior to the midpoint of the antorbital fenestra
 (Loewen et al., 2013:297)
298. Maxilla, number of teeth:
 (0) – 18 or more alveoli
 (1) – 14 to 17 alveoli
 (2) – 11 to 13 alveoli
 (3) – 10 to 1 alveoli
 (Modified from Li et al., 2009: 202; Sereno et al. 2009:61; Carr and Williamson 2010:220; Brusatte et al., 2010:200)
299. Dentary, portion of dentary with teeth:
 (0) – teeth throughout to 65% of the dorsal surface of the dentary
 (1) – teeth restricted to the anterior 33% or edentulous
 (Modified from Li et al., 2009:220)
300. Dentary, number of teeth:
 (0) – 220 or more alveoli
 (1) – 18 to 21 alveoli
 (2) – 15-17 alveoli
 (3) – 14 or less alveoli
 (Modified from Li et al., 2009:84)
301. Tooth spacing on maxilla and dentary:
 (0) – closely appressed
 (1) – widely spaced with considerable separation between alveoli
 (Loewen et al., 2013:301)
302. Maxillary and dentary, mid position tooth serration symmetry:
 (0) – proximal and distal carina serrations similar in size
 (1) – clear asymmetry in serrations on proximal and distal carina or without serrations on either surface
 (2) – lacking serrations
 (Modified from Holtz, 2004; Li et al., 2009:83)
303. Maxillary and dentary teeth, form:
 (0) – ziphodont, transverse width of base less than 60% of mesiodistal length
 (1) – thickened, width greater than 60% of length
 (2) – thickened, width nearly equal to length
 (3) – round
 (Sereno et al. 2009:62; Brusatte et al., 2010:201)

Axis

304. Axis, pleurocoel:
 (0) – absent
 (1) – present
 (Modified from Rowe and Gauthier, 1990; Smith et al., 2007; Li et al., 2009:142; Brusatte et al., 2010:203)

305. Axis, pleurocoel position:
    (0) – midheight of centrum
    (1) – dorsally located, directly underneath neurocentral suture and directly posterior to diapophysis
    (Modified from Rowe and Gauthier, 1990; Smith et al., 2007; Li et al., 2009:142; Brusatte et al., 2010:203)
306. Axis, pneumatic foramen, extent of surrounding fossa:
    (0) – limited to margins of foramen
    (1) – extensive, occupies most of lateral surface of centrum
    (Brusatte et al., 2010:204)
307. Axis, anteroposterior length of centrum compared to dorsoventral height of posterior centrum face:
    (0) – longer than tall
    (1) – shorter than tall, or subequal
    (Modified from Holtz, 2001; Brusatte et al., 2010:202)
308. Axis, ridge on ventral surface of centrum:
    (0) – absent
    (1) – present
    (Brusatte et al., 2010:205)
309. Axis, axial parapophyses and diapophyses:
    (0) – moderate/prominent
    (1) – reduced/absent
    (Rowe, 1989; Smith et al., 2007:143)
310. Axis, neural spine form in lateral view:
    (0) – sheet-like
    (1) – anteroposteriorly reduced and rod-like
    (Molnar et al., 1990; Smith et al., 2007:145)
311. Axis, neural spine in cranial view:
    (0) – flared transversely
    (1) – compressed mediolaterally
    (Li et al., 2009:94)
312. Axis, pneumatic foramina and fossae on each side of the anterior ridge on the neural spine:
    (0) – absent
    (1) – present
    (Brusatte et al., 2010:206)
313. Axis, neural spine, texture of dorsal region of anterior surface:
    (0) – generally smooth or with subtle texture
    (1) – highly rugose, with series of grooves, ridges, and eminences
    (Brusatte et al., 2010:207)
314. Axis, epineurapohyseal processes on axis neural spine:
    (0) – absent
    (1) – present
    (Li et al., 2009:277)
315. Axis, epipophyses, posterior extent:
    (0) – absent or terminates well anterior to postzygapophysis
    (1) – extends to, or only slightly posterior to, the posterior margin of the postzygapophysis
    (2) – large, rugose flange that extends posterior to postzygapophysis
    (3) – elongate flange that extends posteriorly 75% or more than the anteriorposterior length of the postzygapophysis
    (Modified from Brusatte et al., 2010:210)

Cervical Vertebrae

316. Cervical vertebrae, number of pneumatic openings in anterior centra:
    (0) – one pneumatic opening per side
    (1) – two pneumatic opening per side
    (Gauthier, 1986; Li et al., 2009:100)

317. Cervical vertebrae, form of centra:
(0) – amphiceolous to platycoelous
(1) – strongly opisthocoelous
(Gauthier, 1986; Smith et al., 2007:151)
318. Cervical vertebrae, epipophyses of C3-5, form:
(0) – absent or terminates well anterior to postzygapophysis
(1) – extends to, or only slightly posterior to, the posterior margin of the postzygapophysis
(2) – large, rugose flange that extends posterior to postzygapophysis
(Modified from Brusatte et al., 2010:210)
319. Cervical vertebrae, position of posterior edge of anterior cervical centra:
(0) – level with or anterior to the posterior extent of neural arch
(1) – extending beyond posterior limit of neural arch.
(Li et al., 2009:96)
320. Cervical vertebrae, anterior cervical centra, shape in anterior view:
(0) – subcircular or square
(1) – distinctly wider than high; kidney shaped
(Gauthier, 1986; Li et al., 2009:98)
321. Cervical vertebrae, anteroposterior length of centrum compared to dorsoventral height of posterior centrum face:
(0) – longer than tall
(1) – shorter than tall, or subequal
(Modified from Holtz, 2001; Brusatte et al., 2010:202)
322. Cervical vertebrae, neural spine positon:
(0) – centroid of neural spine positioned on anterior half of vertebrae
(1) – centroid of neural spine positioned posterior to the midpoint of vertebrae
(Loewen et al., 2013:322)
323. Cervical vertebrae, neural spine anterioposterior minimum length:
(0) – wide, ½ or more than the length of the centrum
(1) – narrow, less than ½ the length of the centrum
(Loewen et al., 2013:323)
324. Cervical vertebrae, neural spine dorsoventral height:
(0) – short, less than the height of the centrum
(1) – low and centered on neural arch, giving arch an “X” shape in dorsal view
(2) – approximately the same height as the posterior centrum face
(3) – tall, 1.5 or more than the height of the centrum face
(Modified from Makovicky and Sues, 1998; Holtz, 2001; Li et al., 2009:99; Brusatte et al., 2010:212)
325. Cervical vertebrae, neural spine mediolateral thickness:
(0) – thin
(1) – anteroposterally tapering thin to thick
(2) – thick, stout rectangle, base is nearly square with nearly equal anteroposterior and transverse dimensions
(Modified from Holtz, 2004; Benson, 2008; Brusatte et al., 2010:211)
326. Cervical vertebrae, rugose ligament attachment scars on anterior and posterior surfaces of neural spine:
(0) – absent or weakly developed
(1) – present as prominent, flanges visible in lateral view
(Loewen et al., 2013:326)
327. Cervical vertebrae, orientation of posterior centrodiapophyseal lamina in C3-5:
(0) – nearly horizontal, fossa located primarily dorsal to lamina
(1) – projects posteroventrally, infrapostzygapophyseal fossa located primarily posterior to lamina
(Modified from Brusatte et al., 2010:216)
328. Cervical vertebrae, position of prezygapophysis in middle cervicals:
(0) – slightly overhangs centrum laterally
(1) – strongly overhangs centrum laterally, entire prezygapophyseal facet placed lateral to centrum
(Brusatte et al., 2010:215)

Dorsal Vertebrae

329. Dorsal centra pneumaticity:
    (0) – apneumatic
    (1) – pneumaticity limited to anterior dorsals
    (2) – present throughout dorsal column
    (Modified from Li et al., 2009:106 and Brusatte et al., 2010:218; Loewen et al., 2013: 329 now run as unordered)
330. Dorsal vertebrae, ratio of dorsoventral height of posterior centrum face to anteroposterior length of middle-posterior dorsal centra:
    (0) – long and low, less than 75%
    (1) – short and tall greater than 75%
    (Carpenter et al., 2005; Brusatte et al., 2009:210; Carr and Williamson 2010: 228; Brusatte et al., 2010:220)
331. Dorsal vertebrae, anterior dorsal neural spines, level of posterior termination:
    (0) – at approximately the same level as the posterior centrum face
    (1) – far posterior to the posterior centrum face
    (Modified from Brusatte et al., 2009:209; Carr and Williamson 2010:227; Brusatte et al., 2010:219)
332. Dorsal vertebrae, mid- posterior dorsal neural spines, level of posterior termination:
    (0) – at approximately the same level as the posterior centrum face
    (1) – far posterior to the posterior centrum face
    (Modified from Brusatte et al., 2009:209; Carr and Williamson 2010:227; Brusatte et al., 2010:219)
333. Dorsal vertebrae, anterioposterior minimum width of mid-dorsal neural spine in lateral view:
    (0) – wide, subequal to centrum length
    (1) – narrow, ½ centrum length or less
    (Loewen et al., 2013:333)
334. Dorsal vertebrae, dorsoventral height of mid-dorsal neural spine:
    (0) – short, subequal to or less than centrum height
    (1) – tall, 1.5 x centrum height or more
    (Loewen et al., 2013:334)
335. Dorsal vertebrae, rugose ligament attachment scars on anterior and posterior surfaces of neural spine:
    (0) – absent or weakly developed
    (1) – present as prominent, flanges visible in lateral view
    (Loewen et al., 2013:335)
336. Neural spines on posterior dorsal vertebrae, shape in lateral view:
    (0) – rectangular or square
    (1) – anteroposteriorly expanded distally, fan-shaped
    (Li et al., 2009:209)
337. Ventral keel in anterior dorsals:
    (0) – absent or very poorly developed
    (1) – pronounced
    (Rauhut, 2003; Smith et al., 2007:170)
338. Posteriormost dorsals, parapophyses position:
    (0) – on the same level as transverse process
    (1) – distinctly below transverse process
    (Makovicky, 1995; Smith et al., 2007:179)

Sacral Vertebrae

339. Sacral vertebrae, number of sacral vertebrae:
    (0) – 5 or less
    (1) – 6
    (Li et al., 2009:110)
340. Sacral vertebrae, length of anterior sacrals and posterior dorsal centrum length:
    (0) – subequal in length
    (1) – sacrals substantively longer than posterior dorsal centra.
    (Dal Sasson and Maganuco, 2011)

341. Sacral vertebrae, pleurocoels or foramina on lateral surfaces of centra:
(0) – absent on sacral vertebrae
(1) – present on anterior sacrals only
(Li et al., 2009:113; Carr and Williamson 2010:229; Brusatte et al., 2010:223)
342. Sacral vertebrae, fenestrae between fused neural spines:
(0) – neural spines unfused
(1) – spines fused but fenestrae absent
(2) – spines fused and fenestrae present
(Brusatte et al., 2010:224)
343. Sacral ribs, position of ribs on sacrum:
(0) – span two sacrals
(1) – limited to a single sacral
(Brusatte et al., 2010:225)
344. Sacral ribs, position of rib attachment for ribs on individual sacrals:
(0) – span centrum and neural arch
(1) – limited to neural arch only
(Brusatte et al., 2010:226)
345. Sacral vertebra five, position of ventral margin of posterior articular face in lateral view:
(0) – at same level as ventral margin of anterior articular face
(1) – positioned ventral to ventral margin of anterior articular face
(Brusatte et al., 2010:227)

Caudal Vertebrae
346. Caudal vertebrae: presence of transverse processes:
(0) – on more than 16 posteriors
(1) – on 12 to 16
(2) – on fewer than 12 posteriors
(Holtz, 2004; Brusatte et al., 2009:214)
347. Caudal vertebrae, transition point in caudal series at which transverse processes end:
(0) – begins distal to the 10th posterior
(1) – between the 5th and 10th posterior vertebra
(Li et al., 2009:116)
348. Caudal vertebrae, anterior caudal centra shape:
(0) – tall, oval or round in cross section
(1) – with box-like centra in posteriors I-V
(Gauthier, 1986; Li et al., 2009:117)
349. Caudal vertebrae, anterior caudals, position of posterior end of base of neural spine:
(0) – anterior to posterior surface of centrum
(1) – or level with or posterior to posterior surface of centrum
(Brusatte et al., 2010:229)
350. Caudal vertebrae, anterior caudals, shape of transverse processes in dorsal view:
(0) – rectangular, with parallel anterior and posterior sides, or slightly ovoid with a gradual expansion in width distally
(1) – distal end expanded into a spatulate bulb
(Brusatte et al., 2010:230)
351. Caudal vertebrae, anterior caudals, two laminae linking prezygapophysis and transverse process, between which is a triangular fossa:
(0) – absent
(1) – present, shallow fossa
(2) – present, deep fossa
(Modified from Brusatte et al., 2010:231; Loewen et al., 2013:352 now run as unordered)
352. Caudal vertebrae, neural spines:
(0) – simple, undivided
(1) – separated into anterior and posterior alae throughout much of caudal sequence
(Russell and Dong, 1993; Li et al., 2009:118)

353. Caudal vertebrae, anterior spur in front of neural spine in mid-caudals:
- (0) – absent
- (1) – present
- (Makovicky, 1995; Smith et al., 2007:198)

354. Caudal vertebrae, distal caudal neural spine shape:
- (0) – form a low ridge
- (1) – spine absent
- (Modified from Russell and Dong, 1993; Li et al., 2009:119)

355. Caudal vertebrae, mid to distal caudal postzygapophyses length:
- (0) – short between 20% to 100% centrum length
- (1) – extremely long, between 150% to 1000% centrum length
- (Modified Li et al., 2009:120)

Chevrons

356. Chevrons, anterior process of chevron base:
- (0) – absent/weak
- (1) – large
- (Molnar et al., 1990; Smith et al., 2007:199)

357. Chevrons of anterior caudals, form of distal end:
- (0) – short anteroposteriorly, shaft cylindrical
- (1) – distal end elongate anteroposteriorly, flattened and plate-like
- (Modified from Smith et al., 2007:200)

358. Chevrons, form of mid-caudal chevrons:
- (0) – rod-like or only slightly expanded ventrally
- (1) – L-shaped
- (Sereno et al., 1996; Smith et al., 2007:201)

359. Chevrons, form of distal caudal chevrons:
- (0) – rod-like or L-shaped
- (1) – skid-like or T-shaped
- (Rauhut, 2003; Smith et al., 2007:202)

Ribs and Gastralia

360. Pneumatic excavations in cervical or dorsal rib heads:
- (0) – absent
- (1) – present
- (Harris, 1998; Smith et al., 2007:165)

Forelimb

361. Scapula, length relative to humerus:
- (0) – longer than humerus
- (1) – shorter than humerus
- (Li et al., 2009:139)

362. Radius and ulna separation:
- (0) – radius and ulna clearly separate elements
- (1) – distinct adherence or syndesmosis distally
- (Li et al., 2009:214)

Scapula

363. Scapula general form:
- (0) – short and broad (ratio length/minimal height of shaft <9)
- (1) – slender and elongate (ratio >10)
- (Perez-Moreno et al., 1993; Smith et al., 2007:212)

364. Scapula, dorsal expansion of proximal blade measured from the dorsal surface at the scapulocoracoid suture to the most ventrally displaced dorsal surface of the scapular blade:
    (0) – not more than twice the minimum scapular blade width
    (1) – twice to 2.5 times the minimum scapular blade width
    (2) – expanded more than three times the minimum scapular width
    (3) – expanded more than four times the minimum scapular width
    (Loewen et al., 2013:364; now run as unordered)
365. Scapula, angle between dorsal arcromion process and dorsal surface of scapular blade:
    (0) – sweeping curve
    (1) – distinct angle greater than 100 degrees
    (2) – distinct angle less than 95 degrees
    (Modified from Brusatte et al 2009:216; Sereno et al. 2009: 68; Carr and Williamson 2010: 234; Brusatte et al., 2010:232)
366. Scapula, distal end expansion relative to proximal portion of scapula:
    (0) – markedly expanded
    (1) – not expanded
    (Gauthier, 1986; Smith et al., 2007:213)
367. Scapula, ratio of dorsoventral depth of distal expansion to minimum dorsoventral depth of blade:
    (0) – less than 2.5
    (1) – greater than 2.5
    (Sereno et al. 2009: 70; Brusatte et al., 2010:235)
368. Scapula, acromion margin in rostrodorsal view:
    (0) – continuous with blade in anterior aspect
    (1) – anterior edge laterally everted relative to plane of scapular blade
    (Li et al., 2009:133)
369. Scapula, acromion margin length:
    (0) – long, either tapering or horizontal triangle
    (1) – truncated with deep base but short reach beyond scapular blade and squared-off profile
    (Li et al., 2009:279)
370. Scapula and coracoid form in posterior and anterior views:
    (0) – continuous arc
    (1) – coracoid inflected medially, scapulocoracoid 'L' shaped in lateral view
    (Li et al., 2009:137)

Coracoid

371. Coracoid in lateral view:
    (0) – subcircular, with shallow ventral process
    (1) – subquadrangular with extensive ventral process
    (2) – shallow ventral blade with elongate ventral process
    (Li et al., 2009:136)
372. Coracoid, anteroposterior length at midpoint:
    (0) – long, 200% or greater than the length of the scapular acromion at midheight
    (1) – short, less than 150% of the length of the scapular acromion at midheight
    (Modified from Sereno et al. 2009: 71; Brusatte et al., 2010:237)
373. Posterolateral surface of coracoid ventral to glenoid fossa:
    (0) – unexpanded
    (1) – posterolateral edge of coracoids expanded to form triangular subglenoid fossa bounded laterally by enlarged coracoid tuber
    (Li et al., 2009:134)
374. Ventral part of coracoid anterior to the glenoid facet:
    (0) – approximately level with the rim of the facet
    (1) – with tapering posteroventral process
    (Gauthier, 1986; Smith et al., 2007:217)

375. Glenoid fossa orientation:
(0) – faces posteriorly or posterolaterally
(1) – faces laterally
(Li et al., 2009:138)
376. Ossified sternal plates in adults:
(0) – ossified sternal plates absent
(1) – plates ossified, but separate in adults
(Modified from Li et al., 2009: 128)

Humerus

377. Humerus, form of shaft:
(0) – straight
(1) – sigmoid
(Carpenter et al., 2005; Brusatte et al., 2009:218)
378. Humerus, proximal head, morphology:
(0) – low, poorly differentiated, crescentic shaped in proximal view, overhangs posterior surface and does not overhang anterior surface
(1) – enlarged, occupies the majority of the proximal end, bulbous in proximal view, overhangs both posterior and anterior surfaces
(Sereno et al. 2009:73; Brusatte et al., 2010:240)
379. Deltopectoral crest form:
(0) – long, makes up more than 40% or more of the element length
(1) – makes up 30-38% of element length
(2) – short, less than 25% element length, deltopectoral crest less pronounced, forming an arc rather than being quadrangular
(Modified from Li et al., 2009:140 and Brusatte el al., 2010:)
380. Humerus, rotation along shaft, orientation of long axis of proximal end relative to that of distal end:
(0) – approximately 30-45 degree angle
(1) – approximately parallel, shaft rotation absent
(Carr and Williamson 2010:236; Brusatte et al., 2010:242)
381. Humerus, additional muscle attachment tubera at the corner of the anterior and lateral surfaces distal to the deltopectoral crest:
(0) – absent
(1) – present
(Brusatte et al., 2010:243)
382. Humerus, form of distal condyles:
(0) – medial condyle expanded further medially than the lateral condyle is laterally
(1) – lateral and medial condyles expanded equally (offset from shaft in anterior or posterior view is equal)
(Brusatte et al., 2010:245)
383. Humerus, length relative to the femur:
(0) – 45% or more
(1) – 40-30%
(2) – 25-30%
(Carr and Williamson 2010: 235; Sereno et al. 2009: 72; Brusatte et al., 2010:239)

Ulna

384. Ulna, shaft axis, form:
(0) – bowed
(1) – straight
(Li et al., 2009; Sereno et al. 2009:75; Brusatte et al., 2010:246)
385. Ulna, distal articular surface, form:
(0) – convex
(1) – flat
(Sereno et al. 2009:76; Brusatte et al., 2010:247)

Manus

386. Distal carpal, principal distal carpal shape:
    - (0) – semilunate in lateral view with trochlear proximal surface
    - (1) – discoid with flat proximal surface
    - (Li et al. 2010:276; Carr and Williamson 2010:238; Brusatte et al., 2010:248)
387. Manus, number of metacarpals present:
    - (0) – 5
    - (1) – 4
    - (2) – 3
    - (Modified from after Li et al., 2009:150)
388. Manus, number of digits with phalanges:
    - (0) – 4
    - (1) – 3
    - (2) – 2
    - (Modified from Li et al., 2009: 270; Sereno et al. 2009:82; Brusatte et al., 2010:249)
389. Metacarpal I, medial distal condyle, size:
    - (0) – well-developed and large
    - (1) – rudimentary
    - (Sereno et al. 2009:78; Brusatte et al., 2010:250)
390. Metacarpal II, ratio of the length of metacarpal II to the length of metacarpal I:
    - (0) – metacarpal II is 2 or more times the length of metacarpal I
    - (1) – metacarpal II subequal to the length of metacarpal I
    - (2) – metacarpal II is less than 1.8 times the length of metacarpal I
    - (Modified from Sereno et al. 2009:79; Brusatte et al., 2010:252)
391. Metacarpal II, mediolateral width at midpoint:
    - (0) – equal to or narrower than metacarpal I
    - (1) – more robust than metacarpal I
    - (Carr and Williamson 2010: 253)
392. Metacarpal III, length:
    - (0) – subequal in length to MC II
    - (1) – much shorter than MC II
    - (Holtz, 2004; Brusatte et al., 2009:223)
393. Manual phalanx II-1, shaft diameter:
    - (0) – less than shaft diameter of radius
    - (1) – greater than shaft diameter of radius
    - (Li et al., 2009: 210)
394. Manual phalanx II-1, length:
    - (0) – longer than metacarpal I
    - (1) – subequal to metacarpal I
    - (2) – shorter than metacarpal I
    - (Sereno et al. 2009:81; Brusatte et al., 2010:254)
395. Manual unguals, flexor tubercle, form:
    - (0) – large, robust, rugose, conical structure
    - (1) – reduced to a small convexity
    - (Brusatte et al., 2010:255)
396. Manual unguals, degree of recurvature:
    - (0) – extensive, flexor margin deeply concave
    - (1) – thin and reduced, flexor margin shallowly concave
    - (2) – robust and reduced, flexor margin shallowly concave
    - (Modified after Brusatte et al., 2010:256)
397. Manual unguals, proximodorsal lip on some manual unguals, a transverse ridge immediately dorsal to the articulating surface:
    - (0) – absent
    - (1) – present
    - (Li et al., 2009: 153)

Ilium

398. Ilium, overall shape of blade:
    (0) – sub-quadrangular or trapezoidal in lateral view
    (1) – semi-oval outline in lateral view
    (Modified from Benson, 2008)
399. Ilium, dorsal margin, shape:
    (0) – smoothly convex or flat across entire length
    (1) – convex anteriorly and straightens out posteriorly
    (2) – straight anteriorly and transitions to convex posteriorly
    (Modified from Brusatte et al., 2010:266)
400. Ilium, length compared to length of femur:
    (0) – ilium significantly shorter than the femur (70-85%)
    (1) – almost the same length (95-105%)
    (2) – ilium significantly longer (105-115%)
    (Xu et al., 2004; Brusatte et al., 2009:233; Sereno et al. 2009: 83; Brusatte et al., 2010:257)
401. Ilium, blade height vs. length:
    (0) – low and long
    (1) – tall and short
    (Loewen et al., 2013:401)
402. Ilium, iliac blade orientation:
    (0) – roughly vertical
    (1) – dorsomedially inclined, dorsal edges of ilia approach or meet each other above sacrum
    (Modified from Li et al., 2009:265)
403. Ilium, dorsal margin of blade, position relative to sacral neural spines:
    (0) – separated by a gap
    (1) – lies against neural spines and opposing iliac blades may make contact above neural spines
    (Sereno et al. 2009: 85; Brusatte et al., 2010:260)
404. Ilium, postacetabular blade orientation in dorsal view:
    (0) – subparallel
    (1) – diverge posteriorly
    (Li et al., 2009:159)
405. Ilium, notch on anterodorsal edge of ilium:
    (0) – absent
    (1) – notch at rostrodorsal corner
    (Holtz, 2004; Li et al., 2009:262)
406. Ilium, pronounced anteroventral 'hook' on anterior expansion of ilium:
    (0) – absent preacetabular blade subtriangular
    (1) – ventral edge pendant projection
    (2) – process strongly hooked with recurved posterior margin,
    ORDERED
    (Gauthier, 1986; Rauhut, 2003; Smith et al., 2007:253; Li et al., 2009:154; Sereno et al. 2009: 87; Brusatte et al., 2010:259)
407. Ilium, preacetabular part of ilium length relative to postacetabular ilium:
    (0) – preacetabular ilium shorter
    (1) – roughly as long as postacetabular part of ilium
    (2) – longer than postacetabular ilium
    (Li et al., 2009:155)
408. Ilium, notch between pubic peduncle and pre-acetabular process:
    (0) – wide, with semicircular or tear-drop outline
    (1) – narrow and slit-like
    (Modified from Benson, 2008)

409. Ilium, dorsoventrally oriented midline supraacetabular ridge on lateral face of ilium above acetabulum:
(0) – absent
(1) – present posterodorsally inclined
(2) – present and vertical or anterodorsally oriented
(Rauhut, 2003; Benson 2008; Li et al., 2009:266; Brusatte et al., 2009:228; Sereno et al. 2009: 84; Carr and Williamson 2010: 246-248; Brusatte et al., 2010:258)

410. Ilium, dorsal extent of supraacetabular ridge:
(0) – short, does not extend into the m. iliofibularis scar
(1) – tall, extend onto the m. iliofibularis scar
(Loewen et al., 2013:410)

411. Ilium, postacetabular process shape in lateral view:
(0) – tapering or rounded, posterior margin convex
(1) – squared
(2) – acuminate (pointy)
(Modified from Li et al., 2009:158 and Brusatte et al., 2010:267)

412. Ilium, supraacetabular crest on ilium as a separate process from antitrochanter:
(0) – present, forms "hood" over femoral head
(1) – reduced, not forming hood
(2) – absent
(Li et al., 2009:157)

413. Ilium, acetabular crest, extent on pubic peduncle:
(0) – extensive, extends along most or all of the edge of the peduncle
(1) – limited, discretely offset from acetabular edge of pubic peduncle
(Li et al. 2009: 278; Brusatte et al., 2010:263)

414. Ilium, acetabular crest, maximum lateral projection relative to ischial peduncle:
(0) – significantly greater
(1) – subequal
(Sereno et al. 2009: 91; Brusatte et al., 2010:262)

415. Ilium, pubic peduncle articular facet:
(0) – flat or concave
(1) – convex
(2) – flexed with two facets set at obtuse angle to each other
(Modified from Tykoski and Rowe, 2004; Li et al., 2009:291)

416. Ilium, pubic peduncle articular facet orientation:
(0) – facet is sub-horizontal
(1) – facet faces anteroventrally
(Loewen et al., 2013:416)

417. Ilium, pubic peduncle width in lateral view:
(0) – uniform in width (i.e. unexpanded)
(1) – broadly flared toward pubic articular surface
(Rauhut, 2003; Smith et al., 2007:265).

418. Ilium, pubic and ischial peduncles relative dorsoventral length:
(0) – comparable dorsoventral length
(1) – pubic peduncle expanded and twice or more as long as ischidiac peduncle
(Sereno et al., 1994; Smith et al., 2007:266)

419. Ilium, pubic and ischial peduncles, anteroposterior width at dorsal neck:
(0) – pubic peduncle much larger than ischial peduncle
(1) – both peduncles approximately the same length
(Li et al., 2009: 292; Brusatte et al., 2010:264)

420. Ilium, cuppedicus fossa ('preacetabular fossa' of Hutchinson, 2001a):
(0) – absent
(1) – present, formed as antiliac shelf anterior to acetabulum, extends posteriorly to above anterior end of acetabulum
(2) – present, posterior end of fossa on anterior end of pubic peduncle, anterior to acetabulum
(Modified from Hutchinson, 2001a; Smith et al., 2007:264; Loewen et al., 2013:420 now run as unordered)

421. Ilium, brevis fossa lateral exposure:
   (0) – entire length of fossa visible in lateral view, including posterior portion
   (1) – only the anterior portion of the fossa is visible, the posterior portion of the fossa is hidden by posterior blade of the ilium
   (2) – nearly completely hidden by posterior blade of the ilium
   (Loewen et al., 2013:421)

Pubis

422. Pubis, orientation:
   (0) – propubic
   (1) – pubis vertical or pubis posteriorly oriented (opisthopubic)
   (Li et al., 2009:177; Brusatte et al., 2010:275)
423. Pubis, shaft curvature:
   (0) – shaft curves posteriorly, anteriorly convex curvature
   (1) – straight
   (2) – distal end curves anteriorly, anterior surface of shaft concave
   (Calvo et al., 2004; Li et al., 2009:180; Carr and Williamson 2010: 252; Brusatte et al., 2010:269)
424. Pubis, pubic boot, posterior expansion:
   (0) – present
   (1) – little or no posterior process
   (Li et al., 2009:178)
425. Pubis, pubic boot, anterior expansion:
   (0) – absent
   (1) – present, short
   (2) – present, long
   (Brusatte et al., 2009:238,239; Loewen et al. 2013:425 now run as unordered)
426. Pubis, pubic boot, anterior process, length relative to posterior process, measured from line down the middle of the shaft:
   (0) – posterior process much longer
   (1) – subequal
   (Carr and Williamson 2010: 256-258; Sereno et al. 2009: 97; Brusatte et al., 2010:273)
427. Pubis, entire pubic boot, anteroposterior length relative to total long axis length of pubis:
   (0) – less than 25%
   (1) – 30-35%
   (2) – 40-55%
   (3) – 60-70%
   (4) – greater than 75%
   (Modified from Brusatte et al., 2010:272)
428. Pubis, ventral margin of boot in lateral view:
   (0) – convex
   (1) – straight
   (Carpenter et al., 2005a; Bursatti et al., 2009:241; Brusatte et al., 2010:274)
429. Pubis, obturator foramen:
   (0) – present
   (1) – open notch present between pubic shaft and ishciadic peduncle
   (2) – neither notch or foramen present
   (Li et al., 2009:273)
430. Pubis, pubic shaft shape:
   (0) – rounded
   (1) –mediolaterally flattened
   (Loewen et al., 2013:430)
431. Pubis, contact between pubes:
   (0) – both pubes meet extensively
   (1) – contact disrupted by a slit-like opening
   (Makovicky et al., 2005; Li et al., 2009:182)

432. Pubis, pubic tubercle on anterior margin just ventral to contact with ilium:
(0) – absent
(1) – present as a convexity on the anterior margin of the pubis
(2) – present as a rugose flange discretely offset from the anterior margin of the pubis and is bordered posteriorly by heavy rugosities on the lateral surface on the obturator region of the pubis
(Brusatte et al., 2009:237; Carr and Williamson 2010: 255; Brusatte et al., 2010:270; Loewen et al. 2013:432 now run as unordered)
433. Pubis, pubic tubercle, position:
(0) – distally positioned, located ventral to the level of the obturator notch
(1) – proximally positioned, located level with or dorsal to the obturator notch
(Brusatte et al., 2010:271)

Ischium

434. Ischium, length compared to the length of the pubis:
(0) – more than two-thirds
(1) – two-thirds or less of pubis length
(Li et al., 2009:173)
435. Ischium, midshaft thickness at compared to pubis midshaft thickness:
(0) – about as thick or thicker than pubic shaft
(1) – thin, half the thickness or less than the pubic shaft
(Modified from Brusatte et al., 2009:247; Sereno et al. 2009: 94; Carr and Williamson 2010: 265; Brusatte et al., 2010:280)
436. Ischium, shaft curvature:
(0) – roughly straight or only gently curved
(1) – curved, ventrally concave
(2) – curved, dorsally concave
(Modified from Makovicky et al., 2005; Li et al., 2009:167)
437. Ischium, ilio-ischiadic contact:
(0) – ischium with slightly concave or flat proximal articular surface that contacts ilium
(1) – with deep socket for reception of peg-like ischial peduncle of ilium
(Li et al., 2009:261)
438. Ischium, transverse cross-section of acetabular rim:
(0) – convex or beveled
(1) – broad shelf with longitudinal sulcus or depression
(Benson, 2008; Li et al., 2009:274)
439. Ischium, ischial boot (expanded distal end relative to midshaft):
(0) – present
(1) – absent
(Li et al., 2009:175; Sereno et al. 2009: 95; Carr and Williamson 2010: 264; Brusatte et al., 2010:279)
440. Ishchium, obturator foramen and flange:
(0) – present
(1) – reduced to notch, flange confluent with pubic peduncle
(2) – notch or foramen absent, U-shaped gap between pubic peduncle and obturator process
(Li et al., 2009:171; Rauhut, 2003; Smith et al., 2007:287)
441. Ischium, position of apex of obturator flange, distance between apex and proximal end of ischium:
(0) – proximal flange, position at less than 20% of ischum length
(1) – flange positioned at approximately 25-30% of ischium length
(2) – flange position at approximately 35% of ischium length
(3) – distal flange, position further distal than 50% of ischium length
(Modified after Brusatte et al., 2010:281)

442. Ischium, broad medially deflected apron from obturator flange to distal end of ischium:
(0) – absent
(1) – present, covers 1/3 to 1/2 the ventral surface between obturator flange to distal end of ischium
(2) – present, covers more than 1/2 the ventral surface between obturator flange to distal end of ischium
(Loewen et al., 2013:442; now run as unordered)

443. Ischium, scar on posterior edge of the proximal end of the ischium:
(0) – absent
(1) – present
(Holtz, 1994; Li et al., 2009:172)

Femur

444. Femur, femoral head orientation:
(0) – directed ventrally
(1) – directed horizontally
(2) – directed dorsally
(Modified from Molnar et al., 1990; Smith et al., 2007:294)

445. Femur, femoral head orientation relative to the distal femoral condyles:
(0) – directed anteromedially
(1) – directed strictly medially
(Holtz, 1994; Smith et al., 2007:293)

446. Femur, fossa on the posterior surface of the femoral head, just lateral to the articular surface:
(0) – absent or shallow
(1) – narrow channel
(2) – deep, wide fossa
(Loewen et al., 2013:446)

447. Femur, anterior trochanter, presence and separation from shaft:
(0) – absent
(1) – separated from greater trochanter by deep cleft
(2) – trochanters separated by small groove
(Modified from Li et al., 2009:184; Loewen et al., 2013:447 now run as unordered)

448. Femur form of anterior trochanter:
(0) – alariform
(1) – cylindrical in cross section
(Li et al., 2009:185)

449. Femur, height of anterior trochanter:
(0) – shorter, terminates further distally ends distal to the height of greater trochanter
(1) – subequal or slightly taller, the two structures extend to approximately the same level proximally
(Li et al., 2009:260; Sereno et al. 2009: 98; Carr and Williamson 2010: 269; Brusatte et al., 2010:284)

450. Femur, vertical ridge on lateral face of anterior trochanter:
(0) – present
(1) – absent
(Norell and Makovicky, 1999; Li et al., 2009:218)

451. Femur, fourth trochanter, position, measurement from proximal margin of head to midpoint of fourth trochanter relative to total length of the femur:
(0) – proximally located, distance 25% or less femoral length
(1) – distally located, distance 30% of greater femoral length
(Modified from Brusatte et al., 2010:288)

452. Femur, circular scar (*M. adductor femoralis 1*) on posterior surface of shaft distal to fourth trochanter, mediolateral position:
(0) – scar absent
(1) – positioned closer to the medial edge of shaft
(2) – positioned closer to the shaft midline
(Modified from Carrano and Hutchinson, 2002; Brusatte et al., 2010:283)

453. Femur, anteromedial crest on anterior surface proximal to medial distal condyle:
(0) – without longitudinal crest
(1) – crest present extending proximally from medial condyle on anterior surface of shaft
(Li et al., 2009:189)

454. Femur, depression (for *M. extensor digitalum longus*) on anterior surface of distal femur.
(0) – absent or extremely shallow, anterior surface relatively flat
(1) – shallow depression present
(2) – deep depression present
(Modified from Molnar et al., 1990; Smith et al., 2007:303; Brusatte et al., 2010:290)

455. Femur, well-developed extensor groove (for *M. extensor digitalum longus*) present on the distal end of anterior surface of the distal femur constricting distal end of the EDL depression:
(0) – groove absent, expressed as a broad concave anterior margin
(1) – groove present and constricted to a wide, deep, U-shaped cleft in distal view
(2) – groove present and constricted to a narrow, deep, U-shaped cleft in distal view and present as an extensive depression on the anterior surface of the femur
(Modified from Molnar et al., 1990; Smith et al., 2007:303; Brusatte et al., 2010:290)

456. Femur, depression between distal condyles in anterior view:
(0) – relatively flat between condyles
(1) – shallow depression between condyles
(2) – deep depression confluent with extensor groove on anterior surface
(Loewen et al., 2013:456)

457. Femur, deep channel between condyles for *M. gastrocnemius* and *flexor digitorum longus* group in distal view:
(0) – no discrete channel present between condyles
(1) – wide channel, wider than deep
(2) – channel deeper than wide
(Loewen et al., 2013:457)

458. Femur, crista tibiofibularis on lateral condyle in distal view:
(0) – no distinct lateral separation in lateral condyle
(1) – distinct lateral constriction into crista tibiofibularis larger than half the mediolateral width of the lateral condyle
(2) – crista tibiofibularis restricted to medial half lateral condyle
(Modified from Brochu, 2002 and Brusatte et al., 2010:289)

Tibia

459. Tibia, length relative to the femur:
(0) – tibia longer (1.05 or greater)
(1) – tibia subequal or shorter (less than 1.00)
(Sereno et al. 2009: 99; Brusatte et al., 2010:292)

460. Tibia, proximal end, anterior process of lateral condyle:
(0) – absent
(1) – present
(Brusatte et al., 2009:252; Carr and Williamson 2010: 270; Brusatte et al., 2010:293)

461. Tibia, lateral ridge on the lateral side of the tibia for connection with fibula:
(0) – extending from the proximal articular surface distally
(1) – clearly separated from proximal articular surface
(Gauthier, 1986 Smith et al., 2007:308)

462. Tibia, lateral condyle on proximal end of tibia:
(0) – confluent with cnemial crest anteriorly in proximal view, or only slightly offset
(1) – strongly offset from cnemial crest
(Rauhut, 2003; Smith et al., 2007:305)

463. Tibia, lateral malleolus, lateral extent:
(0) – limited, mediolateral measure is less than 40% of mediolateral width of adjacent shaft
(1) – extensive, mediolateral measure greater than 40% of mediolateral width of adjacent shaft
(Carr and Williamson 2010: 271; Brusatte et al., 2010:294)

464. Tibia, lateral malleolus, position relative to medial malleolus:
(0) – extend to approximately the same level distally
(1) – lateral malleolus extends significantly further distally than medial malleolus
(Brusatte et al., 2010:295)

Fibula

465. Fibula, excavated medial fossa on the proximal end of the fibula:
(0) – absent
(1) – present, shallow
(2) – present, deep
(Modified from Smith et al., 200; Li et al, 2009:193; Loewen et al., 2013:465 run as unordered)

466. Fibula, position of medial fossa:
(0) – not extensive, proximal border starts about 1/3 the distance between the proximal surface and the iliofibularis tubercle
(1) – fills most of the proximal end of the fibula
(Loewen et al., 2013:466)

467. Fibula, proximodistal cleft in the iliofibularis tubercle:
(0) – absent
(1) – present, resulting in two crests separated by a depressed fossa
(Brochu, 2002; Carr and Williamson 2010: 272; Brusatte et al., 2010:296)

468. Fibula, elongate groove on medial surface distal to iliofibularis turbercle:
(0) – absent
(1) – present, distal fibula medially concave
(Loewen et al., 2013:468)

Astragalus

469. Astragalus, ascending process, form of base:
(0) – confluent with condylar portion
(1) – separated by deep transverse groove
(2) – separated by fossa across base
(Modified after Welles and Long, 1974; Li et al., 2009:197; Brusatte et al., 2010:298)

470. Astragalus, height of ascending process of astragalus (height vs. height of body):
(0) – lower than astragalar body
(1) – higher than the astragalar body, typically covering only lateral half of anterior surface of distal tibia
(2) – more than twice the height of astragalar body
(Welles and Long, 1974; Smith et al., 2007:321; Loewen et al., 2013:470 run as unordered)

471. Astragalus, ascending process height (height vs. width):
(0) – less than half the width of the bone
(1) – half the width of the bone to equal to the width of the bone
(2) – greater than the width of the bone
(Carpenter et al., 2005b; Brusatte et al., 2009:255; Loewen et al., 2013:471 run as unordered)

472. Astragalus, basal extent of ascending process:
(0) – arising out of the lateral part of the astragalar body
(1) – arising out of the complete breadth of the astragalur body
(Welles and Long, 1974; Smith et al., 2007:320; Brusatte et al., 2010:297)

473. Astragaus, anteroposterior thickness of ascending process:
(0) – wedge-shaped/blocky
(1) – plate-like/laminar
(Sereno et al., 1994; Smith et al., 2007:323)

474. Astragalus, ascending process contact with fibula:
(0) – absent or limited
(1) – extensive
(Loewen et al., 2013:474)

475. Astragalus, fibular facet on body of astragalus:
(0) – large and facing partially proximally
(1) – reduced and confined to anterior half of lateral side of astragalus
(2) – strongly reduced, facing laterally or absent
(Holtz, 1994; Carrano et al., 2002; Rauhut, 2003; Smith et al., 2007:319)

476. Astragalar condyles:
(0) – almost entirely below tibia and face distally
(1) – significantly expanded proximally on anterior side of tibia and face anterodistally
(Sereno et al., 1996; Smith et al., 2007:324)

477. Astragalus, horizontal groove across astragalar condyles anteriorly:
(0) – absent
(1) – present
(Welles and Long, 1974; Smith et al., 2007:325)

478. Astragalus, posterior process:
(0) – completely caps distal tibia
(1) – curves only slightly proximally, but not strongly covering the posterior side of the tibia
(2) – significant portion of distal surface exposed on articular facet so that astragalas and cancanium do not cover entire distal tibia
(Allain, 2001; Smith et al., 2007:326)

Calcaneum

479. Calcaneum, articulation with tibia:
(0) – without facet for tibia
(1) – with tibial facet on posteromedial corner or posterior surface
(Sereno et al., 1996; Tykoski, 2005; Smith et al., 2007:328)

Distal tarsals

480. Distal tarsal fusion:
(0) – separate, not fused to metatarsals
(1) – fused, forming metatarsal cap with intercondylar prominence that fuses to metatarsal early in postnatal ontogeny
(Li et al., 2009:199)

Metatarsals

481. Metatarsals, appression of major metatarsal shafts:
(0) – not appressed against each other beyond proximal half of shaft
(1) – appressed throughout most or all of metatarsus, adjacent surfaces of shafts flattened for contact between metatarsals metatarsals closely appressed and distance between II-III and III-IV is approximately equal
(2) –distance between mt III- mt IV greater than that between mt II- mt III
(Modified from Zanno, 2008; Li et al., 2009:287; Brusatte et al., 2010:302)

482. Metatarsals II and IV, relative length when in articulation:
(0) – metatarsals II and IV subequal in length
(1) – mt II longer than mt IV
(2) – mt II shorter than mt IV
(Loewen et al., 2013:482)

483. Metatarsal II, articular scar for metatarsal III on distal portion of lateral surface of shaft, form:
(0) – smooth or subtle texture
(1) – heavily rugose fossa
(Modified from Brusatte et al., 2010:303)

484. Metatarsal II, tear drop shaped articular surface for metatarsal III:
   (0) – absent
   (1) – present
   (Loewen et al., 2013:484)
485. Metatarsal II, relative size of midshaft compared to metatarsal IV:
   (0) – subequal
   (1) – metatarsal II more robust than metatarsal IV
   (Loewen et al., 2013:485)
486. Metatarsal II, lateral surface in proximal view, shape:
   (0) – flat or weakly concave
   (1) – strongly concave
   (Brusatte et al., 2010:304)
487. Metatarsal III, outline of proximal articular surface:
   (0) – rectangular
   (1) – hourglass-shaped
   (2) – wedge-shaped with no exposure on plantar surface
   (Paul, 1984; Smith et al., 2007:335)
488. Metatarsal III, form of midshaft shaft medial border:
   (0) – straight or subtly convex medial border in anterior view
   (1) – medial expansion forming a convexity or bulge along the distal part of the shaft
   (Kobayashi and Barsbold, 2005; Li et al., 2009:289; Brusatte et al., 2010:300)
489. Metatarsal III, proximal shaft exposure:
   (0) – prominently exposed between MT II and MT IV along entire metapodium
   (1) – MT III proximal shaft constricted and much narrower than either II or IV, but still exposed along most of metapodium, subarctometatarsal
   (2) – very pinched, poorly exposed exposed along proximal section of metapodium, arctometatarsal condition, mt III wedge-shaped with proximal end covered anteriorly by contact between metatarsals II and IV
   ORDERED
   (Novas and Pol, 2005: 200; Makovicky et al., 2005; Li et al., 2009:203; Sereno et al. 2009: 100)
490. Metatarsal III, ventral non-articular surface immediately proximal to the distal condyles:
   (0) – concave
   (1) – raised subtriangular platform
   (Sereno et al. 2009: 101; Brusatte et al., 2010:301)
491. Metatarsal IV, proximal end, medial notch for articulation with metatarsal III:
   (0) – absent
   (1) – present, shallow notch
   (2) – present, deep notch
   ORDERED
   (Loewen et al., 2013:491)
492. Metatarsal IV, well-developed posteromedial flange on proximal end for articulation with metatarsal III:
   (0) – absent
   (1) – present
   (Smith et al., 2005:339)
493. Metatarsal IV, midshaft, articular surface for metatarsal III:
   (0) – flat
   (1) – concave
   (Loewen et al., 2013:493)
494. Metatarsal IV, articular surface for metatarsal III tear drop shaped:
   (0) – absent
   (1) – present
   (Loewen et al., 2013:495)

495. Metatarsal IV, scar for the insertion of *M. gastrocnemius lateralis* covering medial third of posterior surface on metatarsal IV:
    (0) – absent or elongate scar
    (1) – narrow oval rugosity
    (Loewen et al., 2013:495)
496. Metatarsal IV, surface between metatarsal III articulation and scar for *M. gastrocnemius lateralis*:
    (0) – flat
    (1) – concave
    (2) – slender plantar ridge between metatarsal III articulation and *M. gastrocnemius lateralis* scar
    (Loewen et al., 2013:496)
497. Metatarsal IV, depth of medial collateral ligament fossa:
    (0) – shallow, not rimmed
    (1) – deep and rimmed
    (Loewen et al., 2013:497)

Pedal Phalanx

498. Pedal phalanges, proximal phalanges of digit II and III, ratio of length to midshaft width:
    (0) – greater than 3.0
    (1) – 2.0 to 3.0
    (2) – less than 2.0
    (Modified from Brusatte et al., 2010:306)
499. Pedal unguals, relative size of unguals II and III:
    (0) – subequal
    (1) – II much larger forming sickle claw
    (Loewen et al., 2013:4)
500. Pedal unguals, lip overhanging proximal articular surface dorsally (on extensor surface):
    (0) – present
    (1) – absent or reduced to a subtle tuber
    (Brusatte et al., 2010:307)
501. Pedal unguals, curvature of flexor surface:
    (0) – curved
    (1) – relatively straight
    (Li et al., 2009:282)

Characters added to Loewen et al. 2013 matrix last scored in Nesbitt et al. 1019

502. Postorbital, anteroposterior width of jugal flange:
    (0) – anteroposteriorly narrow
    (1) – anteroposteriorly expanded to subequal the length of the orbit
    (New Character)
503. Postorbital, posterior extent of cornual boss:
    (0) – extends only to midpoint of ventral process
    (1) – extends well beyond the midpoint of the ventral process
    (New Character)
504. Frontal, scar for posterior process of prefrontal:
    (0) – broad embayment
    (1) – narrow notch, part of frontal wraps lateral to prefrontal
    (New Character)
505. Dentary, shape in dorsal/ventral view:
    (0) – straight
    (1) – concave laterally
    (New Character)
506. Dentary, dorsoventral height of symphysis vs mid element height:
    (0) – subequal in height
    (1) – much deeper symphysis, 125% middentary height
    (New Character)

507. Dentary, dorsoventral minimum thickness of lingual bar in posterior third of tooth row:
(0) – deep; more than half of the height of the lingual bar anteriorly
(1) – shallow, less than half of the height of the lingual bar anterioirly
(New Character)

508. Dentary, step formed dorsal to the anterior end of the lingual bar by the interdentary symphasis:
(0) – absent
(1) – present, diminutive
(2) – large, covering laterally the first alveolus and anterior half of the second alveolus)
(3) – very large, plate-like covers the first and the second alveolus
ORDERED
(New Character)

509. Dentary, number of alveoli:
(0) –14 or more
(1) – 13 or less
(New Character)

510. Dentary, post alveolar dorsal margin:
(0) – straight or gently sloping
(1) – strongly concave and rising up steeply posteriorly
(New Character)

511. Angular, posterior ventral edge:
(0) – straight or convex
(1) – concave
(New Character)
